# Differences in lateral gene transfer in hypersaline versus thermal environments

**DOI:** 10.1186/1471-2148-11-199

**Published:** 2011-07-08

**Authors:** Matthew E Rhodes, John R Spear, Aharon Oren, Christopher H House

**Affiliations:** 1 Penn State Astrobiology Research Center and Department of Geosciences, The Pennsylvania State University, University Park, PA 16802, USA; 2 Division of Environmental Science and Engineering, Colorado School of Mines, Golden, CO 80401, USA; 3 The Institute of Life Sciences and the Moshe Shilo Minerva Center for Marine Biogeochemistry, The Hebrew Institute of Jerusalem, Jerusalem 91904, Israel

**Keywords:** Horizontal Gene Transfer, Transformation, Halophile, Halobacteria, Thermophile, Thermoprotei

## Abstract

**Background:**

The role of lateral gene transfer (LGT) in the evolution of microorganisms is only beginning to be understood. While most LGT events occur between closely related individuals, inter-phylum and inter-domain LGT events are not uncommon. These distant transfer events offer potentially greater fitness advantages and it is for this reason that these "long distance" LGT events may have significantly impacted the evolution of microbes. One mechanism driving distant LGT events is microbial transformation. Theoretically, transformative events can occur between any two species provided that the DNA of one enters the habitat of the other. Two categories of microorganisms that are well-known for LGT are the thermophiles and halophiles.

**Results:**

We identified potential inter-class LGT events into both a thermophilic class of Archaea (Thermoprotei) and a halophilic class of Archaea (Halobacteria). We then categorized these LGT genes as originating in thermophiles and halophiles respectively. While more than 68% of transfer events into Thermoprotei taxa originated in other thermophiles, less than 11% of transfer events into Halobacteria taxa originated in other halophiles.

**Conclusions:**

Our results suggest that there is a fundamental difference between LGT in thermophiles and halophiles. We theorize that the difference lies in the different natures of the environments. While DNA degrades rapidly in thermal environments due to temperature-driven denaturization, hypersaline environments are adept at preserving DNA. Furthermore, most hypersaline environments, as topographical minima, are natural collectors of cellular debris. Thus halophiles would in theory be exposed to a greater diversity and quantity of extracellular DNA than thermophiles.

## Background

The extent and role of lateral gene transfer (LGT) as a force of evolution has only recently become appreciated. Only in the past couple decades has the sequencing of genomes such as that of *Thermotoga maritima *thrust LGT into the limelight [1]. The original estimates suggested that over 20% of *Thermotoga maritima*'s genome was the result of long distance LGT events. This and numerous other results have led to a potential reevaluation of the tree of life and the notion of a Last Universal Common Ancestor [2,3].

LGT itself is driven by a variety of mechanisms including conjugation, or the transfer of genetic material via direct contact [4], transduction, or the viral mediated transfer of DNA [5], and transformation, or the uptake and incorporation of naked DNA from an environment [6]. Conjugative transfers necessitate the cohabitation of the participants and are generally thought to require the participants to be closely related, although inter-class conjugative events have been shown to occur between members of the Proteobacteria [7]. Similarly, while most transductive phages and phage like objects are restricted to infecting members of the same species, phages that infect across classes are known to exist [8]. Finally, transformative events present no definitive phylogenetic barrier. Presumably a microorganism can take up virtually any DNA present in its immediate environment. However the probability of a harvested piece of assembled DNA being incorporated into a genome is partially dependent on sequence similarity between the donor and host DNA and is therefore much greater for closely related individuals [9]. Consequently the vast majority of LGT events are thought to occur between closely related species. Nevertheless inter-phylum and inter-domain transfer events can and do occur [1,10]. These "long range" transfer events are partially the result of transformation events and, while relatively rare, offer a potentially significant evolutionary mechanism.

Species within the domain Archaea and a variety of bacterial phyla are known to be capable of transformation [9]. Preliminary estimates indicate that approximately 1% of bacterial species are naturally able to take up DNA [11]. The frequency of a transformation event is dependent on a number of factors, including but not limited to, the quantity of DNA in an environment, the rate of DNA degradation in an environment, the frequency of DNA uptake by the recipients, the likelihood of incorporation into a genome, and natural selection on the incorporated DNA [9]. These factors in turn are highly specific to individual species and environments. Here, we have used genomic and metagenomic techniques to test mechanisms of LGT into two phylogenetically coherent clades from different extreme environments.

### Halophiles and Thermophiles-

Extremophiles, and in particular thermophiles and halophiles, are well-known for participating in rampant LGT [1,12,13]. It is theorized that the very nature of their extreme environments encourages the exchange of genetic material. Essentially any advantages gained in overcoming the environmental challenges are highly sought after, rapidly exchanged, and potentially accelerate the rate of evolution. In this regard, thermal and saline environments are quite similar. Both offer considerable environmental obstacles to be overcome before life can persist.

The crenarchaeal class, Thermoprotei, consists solely of obligate thermophiles. Similarly, the euryarchaeal class, Halobacteria, consists solely of obligate halophiles. Any LGT event into a member of the Thermoprotei or the Halobacteria necessarily occurred in either a thermal or hypersaline environment respectively. Thus, together these two distinct archaeal lineages offer a naturally occurring evolutionary experiment by which we can study "long range," inter-class and more distant, LGT events in these specific environments.

However, with regard to transformation, there are some significant differences between these two types of extreme environments. For example, high temperatures rapidly degrade unprotected DNA, both intracellularly and extracellularly, thereby preferentially preserving more thermally protected DNA. Fittingly, certain proteins, enzymes, and specifically salts, such as MgCl_2 _and KCl, can help protect DNA from thermal degradation [14,15]. In contrast, high salinities can preserve even naked DNA for exceptionally long periods of time. Borin *et al*. demonstrated that the preservation of naked DNA in deep-sea anoxic hypersaline brines did not depend on the species of origin and that DNA was often capable of participating in natural transformation after weeks of exposure [16]. Another fundamental difference between thermal and saline environments is that saline environments almost as a rule are topographical minima. Saline environments such as the Dead Sea are therefore natural collectors of cellular debris and may therefore contain the DNA of a diversity of contaminant species [17]. Thermal environments, however, may or may not be topographical minima and therefore may or may not be natural collectors of cellular debris. Environmental factors would therefore serve to increase the diversity of extracellular DNA in a typical saline environment relative to the typical thermal environment.

Analyses of halophiles have revealed a number of genomic characteristics common to halophilicity. Foremost amongst these characteristics is a propensity for GC richness possibly to protect against thymine dimerization due to the intense UV radiation often associated with hypersaline environments [18]. The preference for GC nucleotides is present in virtually all known lineages of halophiles and is nearly ubiquitous amongst the Halobacteria, *Haloquadratum walsbyi *being the sole known exception. However interspersed among the GC rich genomes of the Halobacteria are many GC poor regions [18]. The varied composition of the Halobacteria genomes combined with the diversity of metabolic functions and the frequent occurrence of insertion sequence elements suggested to Kennedy *et al*. that the Halobacteria are particularly adept at procuring novel genes and metabolic pathways [18].

Other DNA level propensities include an increased abundance of the dinucleotides 'CG', 'GA/TC', and 'AC/GT' and preferences for specific codons for the amino acids arginine, cysteine, leucine, threonine, and valine, presumably for secondary and tertiary stability in protein folding [19]. Furthermore, halophiles have developed two distinct strategies to overcome the extreme salinities of their native environments. While the "salt-out" halophiles balance the osmotic pressure of their environments with intra-cellular organic solutes such as betaine, the "salt-in" halophiles use KCl. The presence of often multimolar concentrations of K^+ ^ions requires radical alterations of protein chemistry. These alterations in protein chemistry include an overall preference for amino acids with acidic residues relative to amino acids with basic residues [20]. The halophiles of the class Halobacteria are all "salt-in" and they all demonstrate a bias toward amino acids with acidic residues, regardless of their nucleotide composition. Recent metagenomic studies have confirmed this trend on an environmental scale in a number of hypersaline environments [17].

At a salinity of over 340 g/l, the modern surface waters of the Dead Sea represent one of the most saline naturally occurring bodies of water known to harbor life. When combined with a slightly acidic pH (~ 6), near toxic magnesium levels, (currently about 2.0 M Mg^2+^), and dominance of divalent cations over monovalent cations [21], it becomes a truly unique and inhospitable ecosystem. Current cell counts are well below 5 × 10^5 ^mL^-1 ^[22]. A number of species of the Halobacteria have been isolated from the Dead Sea, including *Haloarcula marismortui *[23], *Haloferax volcanii *[24], *Halorubrum sodomense *[25], and *Halobaculum gomorrense *[26]. However recent metagenomic studies have suggested that the dominant microorganism in the modern Dead Sea is most closely related to a member of the neutrophilic, halophilic, euryarchaeal genus *Halobacterium *or the alkaliphilic, halophilic, euryarchaeal genus *Natronomonas *[17,27].

### Identifying LGT events-

Putative LGT events are generally identified using two distinct methods: phylogenetic methods attempt to identify genes associated with LGT events by constructing and analyzing phylogenies in an effort to find genes that do not conform to the group's established taxonomy. Compositional methods, on the other hand, identify LGT events by searching for genes whose DNA or amino acid signatures do not match those of their host organism. The methods are essentially complementary, in that they use unrelated data to obtain similar conclusions. For this reason the two methods often identify entirely different classes of LGT events [28,29]. Here, in an attempt to investigate the drivers of LGT in thermal and hypersaline environments, we have employed a predominantly phylogenetic approach to identify putative LGT events involving a thermophilic class of Archaea, the Thermoprotei, and a halophilic class of Archaea, the Halobacteria. We then seek to confirm our results in a collection of environmental fosmids from the Dead Sea.

## Results

Genomes from all fully sequenced genera of the archaeal classes Thermoprotei (*Acidilobus*, *Aeropyrum*, *Caldivirga*, *Desulfurococcus*, *Hyperthermus*, *Ignicoccus*, *Ignisphaera*, *Metallosphaera*, *Pyrobaculum*, *Staphylothermus*, *Sulfolobus*, *Thermofilum*, *Thermoproteus, Thermosphaera*, and *Vulcanisaeta*) and Halobacteria (*Halalkalicoccus*, *Haloarcula*, *Halobacterium*, *Haloferax*, *Halomicrobium*, *Haloquadratum*, *Halorhabdus*, *Halorubrum*, *Haloterrigena*, *Natrialba*, and *Natronomonas*), were obtained from the NCBI database in November of 2010. These genomes were then compared to the entire collection of fully sequenced microbes using the BLASTP program and default parameters [30]. In cases where the normalized best BLAST score to members of its own class but not within its genus was less than 75% of the normalized best BLAST score to non-members of the Thermoprotei or Halobacteria respectively, the gene was flagged as a probable inter-class LGT event. Overall this method identified 1226 genes from Halobacteria and 1279 genes from Thermoprotei as "long distance" LGT events. To test for the possibility of bias in our downstream analyses associated with the 75% BLAST score cutoff, the procedure was repeated with cutoffs ranging from 90% to 50%.

**Figure 1:**
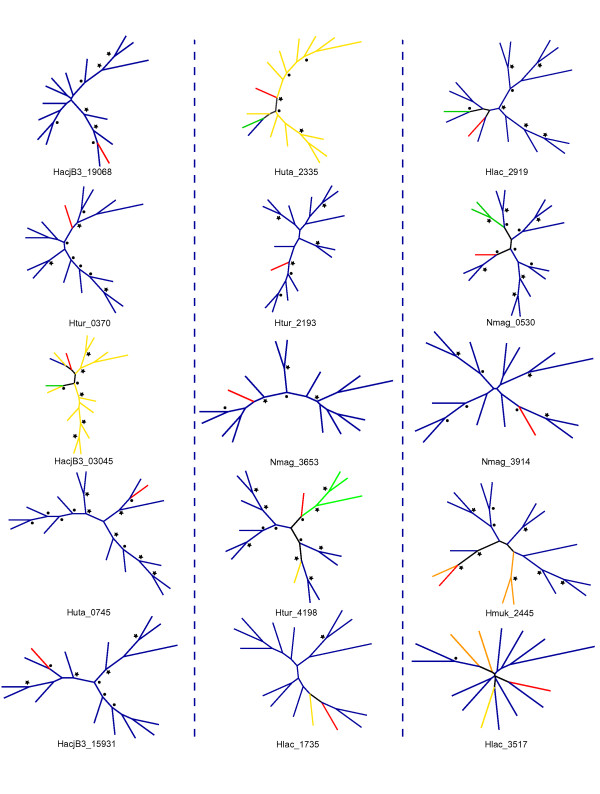
**Phylogenetic trees for Halobacteria LGT genes**. Depiction of 15 randomly selected trees of LGT genes for the Halobacteria. The leftmost column depicts genes from the upper third of BLAST scores, middle column from the middle third, and rightmost column from the bottom third. Target gene is depicted in red and named below the tree, other genes from the same class are shown in orange, genes from the same phylum but not the same class are shown in yellow, genes from the same domain but not the same phylum are shown in green, and genes from a different domain are shown in blue. Circles next to a node indicate a bootstrap value of greater than 50% and stars indicate bootstrap values of greater than 75%.

**Figure 2:**
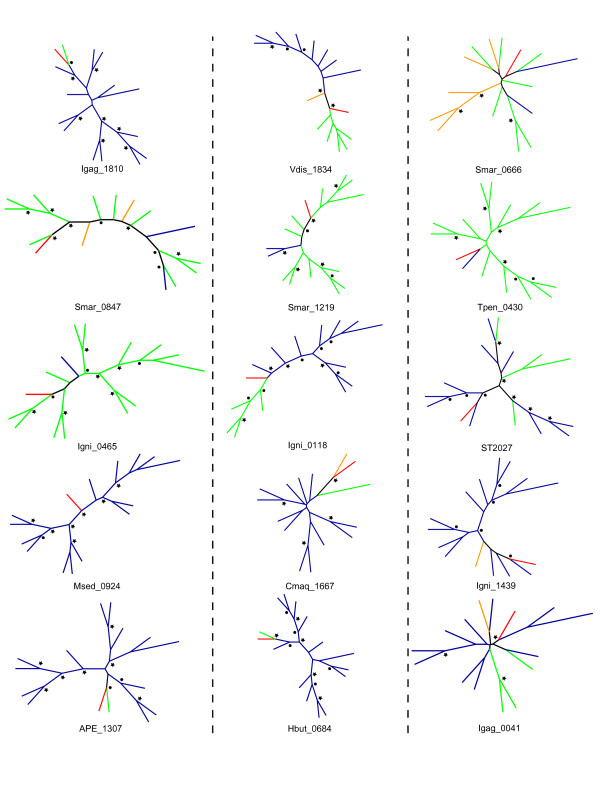
**Phylogenetic trees for Thermoprotei LGT genes**. Depiction of 15 randomly selected trees of LGT genes for the Thermoprotei.

We believe that our algorithm should preferentially identify LGT events into the Halobacteria and Thermoprotei. For the vast majority of LGT events identified the closest homologues were exclusively or almost exclusively from outside of the Halobacteria or Thermoprotei respectively, thereby suggesting that the transfer was into a member of the Halobacteria or Thermoprotei. Nevertheless, to check this we constructed phylogenetic trees for a representative sample of LGT genes from each class using the top homologues in the KEGG database (Figure 1, Figure 2, and SI 1) [31]. As expected, upon inspection, the vast majority of genes showed evidence of having been transferred into the Halobacteria and Thermoprotei. For the remainder, the majority showed phylogenies too disordered to make an accurate statement.

### Assignment to Clusters of Orthologous Groups of proteins (COGs)-

**Figure 3:**
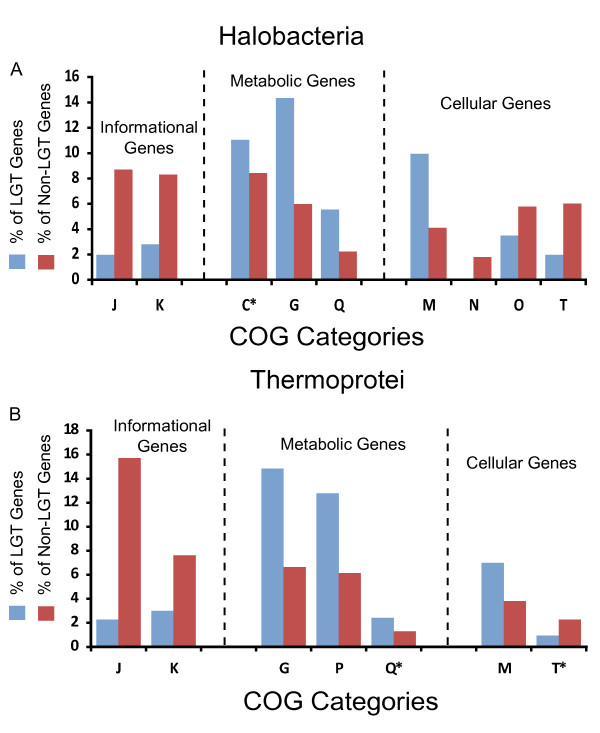
**Assignment of genes to COG categories**. Chart depicting the percentage of genes assigned to various COG categories for both regular genes and genes identified as LGT events into the (a) Halobacteria and (b) Thermoprotei. The COG categories shown demonstrated a statistically significant disparity between LGT genes and non-LGT genes beyond a 95% confidence interval. The categories depicted with an * fell just below the 95% threshold.

Both the genes associated with long distance LGT events and the genes not associated with long distance LGT events were assigned to functional categories according to the classification of the Clusters of Orthologous Groups of proteins (COG) database (Figure 3) [32]. For both the Thermoprotei genomes and the Halobacteria genomes, "Information storage and processing" genes belonging to categories, J (Translation, ribosomal structure and biogenesis) and K (Transcription) are considerably underrepresented amongst genes presumed to have undergone LGT. In a similar vein, "Metabolic" genes belonging to categories C (Energy production and conversion), G (Carbohydrate transport and metabolism), P (Inorganic ion transport and metabolism), and Q (Secondary metabolites biosynthesis, transport and catabolism) are overrepresented amongst genes that have undergone LGT in either the Thermoprotei, the Halobacteria, or both. Genes involved in "Cellular processes and signaling" are inconclusive. While category M (Cell wall/membrane/envelope biogenesis) is overrepresented in genes associate with LGT, categories N (Cell motility), O (Posttranslational modification, protein turnover, chaperones), and T (Signal transduction mechanisms) are underrepresented among genes associated with LGT. As a whole, these results coincide well with the conclusions of Rivera *et al*. [33] that LGT should favor operational genes over informational genes. This is especially true for distantly related LGT events and may offer limited non-phylogenetic evidence that we have in fact identified long distance LGT events [34].

### Homologue Taxonomy

**Figure 4:**
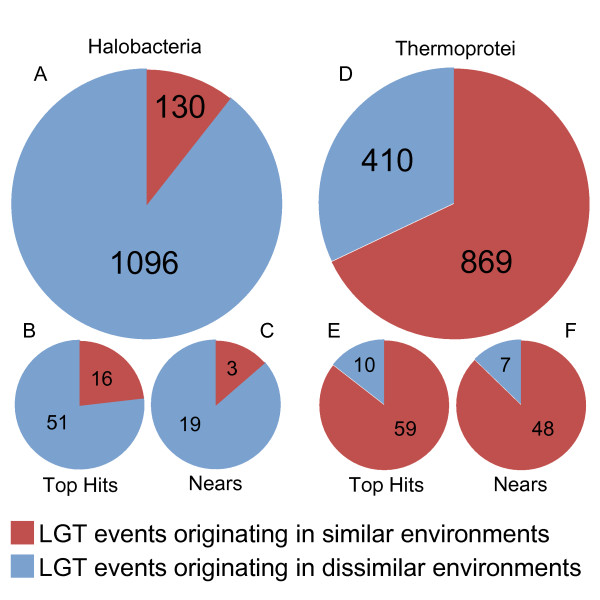
**Origin of LGT genes**. Pie charts depicting the proportion of inter-class LGT events from halophiles into the Halobacteria and from thermophiles into the Thermoprotei for all genes (a and d), only hits with bit scores > 500 (b and e), and only instances where multiple genes were transferred (c and f).

For the genes associated with LGT events, we then identified the species representing the closest homologue to the original archaeal gene as matched by BLAST. We categorized these donor species according to their halophilicity and thermophilicity (Figure 4a-f). As expected, the majority, 68%, of the donor species to the Thermoprotei demonstrate thermophilic character themselves (Figure 4a). Genes originating in thermophiles should be pre-adapted to thermal conditions and therefore should present fewer obstacles to incorporation into a Thermoprotei genome. In stark contrast however, the vast majority, >89%, of donor species to the Halobacteria, were not species with known halophilic character (Figure 4d). This appears to suggest that something other than pre-adaptation to a high salt environment is the determining factor in successful LGT events into Halobacteria and presumably other halophiles. It is worth noting that for both the Thermoprotei and the Halobacteria the proportion of genes identified as intra-environmental LGT events remained consistent regardless of the BLAST cutoff used in the LGT identification step (SI 2).

### Database Bias

There exists a potential bias in our analysis however, in that there are many more fully sequenced thermophiles in the databases than there are halophiles. While it seems unlikely, most if not all of the LGT events from non-halophiles into the Halobacteria could actually originate in heretofore unidentified and/or unsequenced halophiles. The complexity and diversity of hypersaline environments, and for that matter the majority of the microbial world, is poorly constrained [35]. Thus, barring a direct observation of a LGT event from a non-halophile to a member of the Halobacteria, it appears impossible to rule out the possibility that we have not identified the correct donor species. Nevertheless, there are a number of tests that can lend support to our assertion that that the database bias does not account for the vast majority of the discrepancy between LGT into Thermoprotei and Halobacteria. These include:

### 1) Restricting the analysis to only particularly strong matches -

We restricted the analysis to LGT events whose top homologue had a BLAST bit score of greater than 500. Of the 1226 identified LGT events into the Halobacteria, 67 met this criterion. Of these 67, 51 or approximately 76% were to species which do not demonstrate halophilic tendencies (Figure 4b). Thus, as expected we do observe an increase in the proportion of LGT events originating in halophiles relative to non-halophiles. However, the increase only accounts for a small portion of the putative LGT events originating in non-halophiles. Of the top five examples of putative LGT events, only one is to a known halophile, *Salinibacter ruber *(Bacteroidetes). The other four, all with BLAST scores of 963 or better, are to non-halophiles (Table 1). The same analysis was performed on the Thermoprotei LGT genes. Amongst the 1279 identified LGT events into the Thermoprotei, 69 had BLAST scores of greater than 500. Of these 69, only 10, or approximately 14% were to non-thermophilic species (Figure 4e), yielding an even greater increase than for the Halobacteria. Also, all five of the strongest examples of LGT into Thermoprotei originate in thermophilic lineages (Table 1).

**Table 1:** Top scoring LGT genes

Protein Function	Protein ID	Host	Donor	Percent Identical	Score
**Thermoprotei**					

Hypothetical protein	296242776	*Thermosphaera aggregans*	*Thermococcus sibiricus*	75	1667

Cobaltochelatase	146304837	*Metallosphaera sedula*	*Picrophilus torridus*	53	1256

Cellobiose phosphorylase	305664199	*Ignisphaera aggregans*	*Thermotoga maritima*	61	1055

Carbon-monoxide dehydrogenase	229582559	*Sulfolobus islandicus*	*Rhodothermus marinus*	69	1095

Aldehyde dehydrogenase	118431783	*Aeropyrum pernix*	*Thermomicrobium roseum*	62	991

**Halobacteria**					

Homocysteine methyltransferase	110668174	*Haloquadratum walsbyi*	*Salinibacter ruber*	76	1196

Molybdopterin oxidoreductase	284164928	*Haloterrigena turkmenica*	*Hydrogenobacter thermophilus*	46	1054

Hypothetical protein	222481310	*Halorubrum lacusprofundi*	*Hyphomicrobium denitrificans*	44	1029

Alpha amylase catalytic region	257386281	*Halomicrobium mukohataei*	*Thermoanaerobacter mathranii*	64	963

Glycosyltransferase	257052439	*Halorhabdus utahensis*	*Ignisphaera aggregans*	58	963

**Dead Sea Fosmids**					

Acetophenone carboxylase	56476760		*Azoarcus *sp.	45	637

Cytosine Deaminase	221633818		*Thermomicrobium roseum*	47	395

Isopropylaminohydrolase	108803077		*Rubrobacter xylanophilus*	51	375

Alkaline phosphatase	262198659		*Haliangium ochraceum*	40	365

AMP-dependent synthetase	241661738		*Ralstonia pickettii*	39	335

Top five BLAST hits to genes identified as LGT events involving the Halobacteria, Thermoprotei, and a collection of Dead Sea fosmids.

### 2) Restricting the analysis to only LGTs of neighboring gene pairs -

We identified 22 instances within the Halobacteria where adjacent genes or genes separated by a single gene were apparently transferred together and showed conservation not only of gene content, but also of gene order. The inter-class conservation of gene order offers concrete proof that these genes have undergone a LGT event. Of the 22 gene pairings, 19 originated in species with no known halophilic tendencies (Figure 4c), again suggesting that a significant portion of long range LGT events into the Halobacteria did not originate in halophiles. For the Thermoprotei we identified 55 multiple gene transfers, of these, only 7 or 13% originated in non-thermophiles (Figure 4f).

### 3) Looking for the presence of distinctly halophilic traits within the transferred genes-

**Figure 5:**
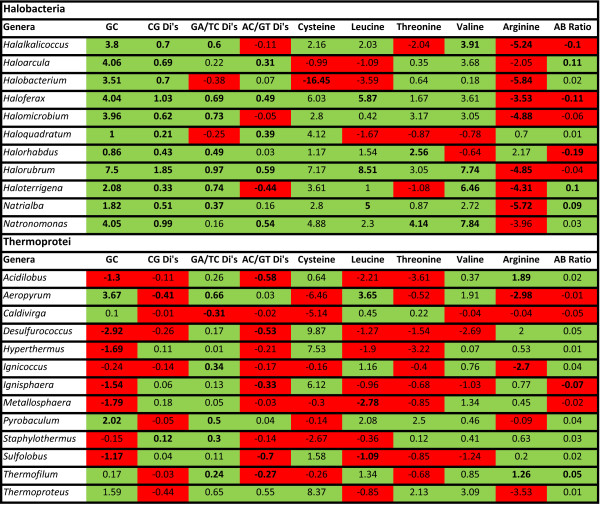
**Genomic halophilicity indicators**. Percent differences for various indicators of halophilicity between LGT genes and non-LGT genes for both the Halobacteria and Thermoprotei. Values for which the LGT genes are less than the non-LGT genes are shaded in green. Values for which the LGT genes are greater than the non-LGT genes are shaded in red. All values that exhibited greater than 95% confidence are shown in bold font. The indicators are: GC - GC content of the gene pools. CG Di's, GA/TC Di's, AC/GT Di's- Difference in preference for 'CG', 'GA/TC', and 'AC/GT' dinucleotides given nucleotide frequencies. Arginine (CGA and CGG), Cysteine (UGU), Leucine (CUC), Threonine (ACG), and Valine (GUC) - Difference in preference for respective codons given nucleotide abundances. AA Bias - Ratio of aspartic acid and glutamic acid to arginine, lysine, and histidine.

As mentioned above, there have been a number of reported genomic indicators of halophilicity. These indicators include genome wide GC content, a preference for the dinucleotides 'CG', 'GA/TC', and 'AC/GT', a number of codon preferences, and an overall bias toward amino acids with acidic residues [19,20]. If a significant portion of the LGT genes did originate in non-halophiles, then values for these indicators would be expected to be higher in the non-LGT halobacterial genes than in the LGT halobacterial genes. These trends should not necessarily be observed for the Thermoprotei. Figure 5 shows a genus by genus breakdown of all values in LGT genes and non-LGT genes for both the Halobacteria and the Thermoprotei. The most robust trends are observed for the GC content and 'CG' dinucleotide preference. All 11 Halobacteria genera demonstrate a statistically significant increase in both GC content and 'CG' dinucleotide preference for the non-LGT genes relative to the LGT genes. This includes the genus *Haloquadratum *which has an anomalously low genomic GC content of 48%. However, as the Thermoprotei do not demonstrate a similar trend, the lowered GC content does not appear to be inherent to LGT events, nor can it be attributed to an artifact of our algorithm.

Both 'GA/TC' and 'AC/GT', also demonstrate a trend toward an increase among the non-LGT genes for the Halobacteria. The trends while not quite as strong, are still apparent. The Thermoprotei show a similar trend for 'GA/TC' and a reverse trend for 'AC/GT'. Amongst the codon biases, cysteine, leucine, threonine, and valine all show a trend toward an increased preference in the halobacterial non-LGT genes. Only arginine demonstrates a reverse trend. Meanwhile, the Thermoprotei do not appear to exhibit any particular trends. Finally, neither the Halobacteria nor the Thermoprotei show a particularly strong trend between LGT genes and non-LGT genes for the amino acid bias.

### Environmental halophiles examples

Twenty-five 40 kb fosmids from the surface water of the 2006 Dead Sea were sequenced on a quarter plate of a 454FLX sequencer. The sequencing run produced a total of 90,479 reads with an average read length of 237 base pairs, for a total of approximately 21 million base pairs of sequence. The sequences were then assembled and a total of 95 contigs with greater than 2,000 base pairs were produced. These contigs were compared to the collection of fully sequenced genomes using a BLASTX search. The contigs were then scanned for the presence of Halobacteria genes, and all contigs without a majority of Halobacteria genes were discarded. The remaining contigs were searched for the presence of genes whose top normalized BLAST score to a member of the Halobacteria was less than 75% of the top normalized BLAST score to any non-member of the Halobacteria. Twenty-two putative "long distance" LGT genes were identified in this manner of which only two were from known halophiles (SI 3). The top five instances are provided in Table 1.

## Discussion

Using a homology-based approach we identified 1,226 putative inter-class LGT events involving members of the obligatory halophilic archaeal class Halobacteria and 1,269 putative inter-class LGT events involving members of the obligatory thermophilic archaeal class Thermoprotei. The vast majority of these LGT events consisted of gene transfers into the Halobacteria and Thermoprotei. Furthermore, the phylogenetic distance between the donor species and the recipient species suggests that the majority of these LGT events were the result of natural transformation. As the Halobacteria are all obligate halophiles and the Thermoprotei are all obligate thermophiles the transformative events must have occurred in saline and thermal environments respectively.

Conventional thinking would suggest that the Halobacteria would be exposed to naked DNA from predominately other halophiles and that the Thermoprotei would be exposed to naked DNA from predominately other thermophiles. Additionally, genes originating in other halophiles and thermophiles would be preadapted to the particular environmental conditions and would therefore be more likely to be successfully transferred. Thus we would expect the majority of LGT events into the Thermoprotei to originate in other thermophiles and the majority of LGT events into the Halobacteria to originate in other halophiles. However, we found that while the majority of these transformational events into the Thermoprotei did in fact originate in other thermophiles, the majority of these transformational events into the Halobacteria did not originate in other known halophiles. This suggests that there is something fundamentally different between LGT in thermophiles and LGT in halophiles.

Unfortunately, as with all studies relying on genomic databases, there is the potential for distortion from database bias. In our study we face three disparate database issues. First, there is always the possibility that we may have misidentified LGT events. Second, while the fully sequenced Thermoprotei originate from a number of distinct orders, the fully sequenced Halobacteria all belong to a single family, the Halobacteriaceae. It is unclear how the reduced phylogenetic diversity of the Halobacteria would affect our analysis. Finally, the relative paucity of fully sequenced halophiles outside the Halobcateria as compared to thermophiles outside the Thermoprotei may explain our observation that more Halobacteria donors are non-halophiles than Thermoprotei donors are non-thermophiles. Nevertheless, a number of halophiles have been sequenced in non-Halobacterial lineages including *Bacillus halodurans *of the bacterial class Bacilli, *Methanohalophilus mahii *and *Methanohalobium evestigatum *of the euryarchaeal class Methanomicrobia, and *Chromohalobacter salexigens *of the bacterial class Gammaproteobacteria. In all four of these cases there remain numerous LGT events apparently originating in close non-halophilic relatives. At the same time we also went to great lengths to seek out additional lines of evidence that would help confirm our findings.

We restricted the analysis to LGT genes with especially strong matches and to instances where multiple genes were transferred together and gene order was conserved. Restricting the analysis to especially strong matches increases the likelihood of having identified both an LGT event and the correct donor species. Restricting the analysis to multiple gene transfers virtually guarantees that a LGT event took place. In both cases we achieved similar results to our initial analysis.

We then investigated independent genomic indicators of halophilicity for each genus of the Halobacteria and Thermoprotei. These indicators consisted of GC content, 'CG', 'GA/TC', and 'AC/GT' dinucleotide content, codon preferences, and amino acid preferences. If the LGT genes into the Halobacteria did in fact originate in non-halophiles, some residual signature of non-halophilicity could remain. Essentially this amounts to an independent assessment of LGT events specifically targeting halophiles. Of the ten indicators investigated for the Halobacteria, eight supported our assertion, one did not indicate a clear trend, and only the codon preference for arginine refuted our conclusion.

The strongest support came from genomic GC content and 'CG' dinucleotide content. Both indicators showed a statistically significant increase from non-LGT genes to LGT genes for every genus and represent strong support for the correct identification of LGT genes and for the non-halophile origin of the majority of them. This includes the genus *Haloquadratum *which is unique among the Halobacteria for having a relatively low genomic GC content of approximately 48%. It therefore might appear that there is something inherent to genes associated with LGT that accounts for the differences in GC content. However, for the Thermoprotei there was no clear trend for the indicators as a whole and for genomic GC content there appeared to be a decrease in genomic GC content from LGT genes to non-LGT genes.

Finally we sought confirmation of our genome based results from within metagenomic samples. We used fully and partially assembled fosmid inserts from the surface waters of the Dead Sea, a highly saline environment, to identify inter-class LGT events in environmental halophiles. Once again the vast majority of the donor species were non-halophiles.

## Conclusions

In this study we provide a number of lines of evidence that suggest that the mechanisms and origins of "long distance" LGT events into the Thermoprotei and Halobacteria are different. We theorize that the difference in the origin of LGT genes lies in the differing natures of hypersaline and thermal environments with respect to naked DNA. Hypersaline environments are often adept at preserving both naked DNA and intact microorganisms. There have even been claims of intact DNA and viable bacteria preserved in 200 million year old salt crystals [36-38]. In contrast, thermal environments rapidly degrade DNA. Thermophilic organisms, therefore, must go to great lengths to protect and stabilize their DNA from the environment. Thus intracellular degradation of DNA would be expected to be greater for non-thermophiles, and thermally stable DNA would be in better condition upon release to the environment. Furthermore various mechanisms of DNA protection, such as association with DNA binding proteins, may provide transient protection extracellularly. The net effect of these and other protective methods would lead to an increase in intact thermophilic DNA in a thermal environment relative to non-thermophilic DNA.

In a related vein, hypersaline environments generally occupy topographic minima. This makes hypersaline environments such as the deep Mediterranean basins and the Dead Sea natural collectors of debris, cellular and otherwise [16,17]. Thus the average halophilic microorganism should be exposed to a much greater diversity of DNA than an average thermophilic microbe. Together these facts suggest that halophilic microorganisms are exposed to a greater proportion of intact non-halophilic DNA than thermophiles are exposed to intact non-thermophilic DNA. This suggestion combined with the relatively large genome size, diverse genomic composition, and broad range of metabolic capabilities of the Halobacteria paint the picture of the Halobacteria potentially acting as the consummate opportunists, incorporating and utilizing genes from a great variety of organisms. However, in order to better identify and understand LGT events amongst halophiles many more halophiles must be sequenced and the dynamics of naked DNA in a variety of naturally occurring settings must be studied.

## Methods

### DNA extraction and fosmid preparation

The Dead Sea environmental sample was collected and processed in 2007 by the Béjà lab group (Technion, Haifa, Israel) according to the protocol of Bodaker *et al*. [27]. The fosmid inserts were then shipped frozen to Penn State. The inserts were run on a 1% low melting point agarose gel to remove residual contamination and the 40 kb band was extracted and digested with the Gelase enzyme (Epicentre). The fosmids were then sequenced on a GS FLX sequencer (454 Life Sciences) on one quarter of a pico-titre plate.

### Fosmid analysis

The fosmid sequences were assembled using the 454 assembler program. All contigs of greater than 2,000 base pairs were compared to the collection of fully sequenced Bacteria and Archaea using the BLASTX program, an e-value of 10^-5^, and default parameters. The contigs were then spliced according to gene location and another identical BLASTX comparison was conducted on each gene. Each gene whose top hit was not to a member of the Halobacteria, had a normalized bit score (BLAST bit score to homologue divided by BLAST bit score to self) more than 25% greater than the best hit to a Halobacteria gene, and had a bit score greater than 67 was flagged as a putative inter-class LGT event. Then all contigs were scanned for genes belonging to the Halobacteria, and contigs without a majority of genes assigned to Halobacteria species were discarded. Finally, a number of genes demonstrated near perfect, upwards of 95%, identity to likely laboratory contaminants such as *Escherichia coli*. These genes were also removed from the analysis. The remaining 22 genes were considered LGT events and the donor species were assigned according to the best hit as matched by BLAST. A web search was then conducted to identify whether the donor species was a known halophile.

### Genome Analysis

All fully sequenced Thermoprotei and Halobacteria genomes were compared to the collection of all fully sequenced Bacteria and Archaea using BLASTP, an e-value of 10^-5^, and default parameters. Each gene whose top non-identical hit was not to a member of the Halobacteria or Thermoprotei respectively, had a normalized bit score more than 25% greater than the best non-identical hit to a member of the Halobacteria or Thermoprotei, and had a bit score greater than 67 was flagged as an inter-class LGT event. In cases such as *Pyrobaculum *where more than one species has been sequenced, the analysis was conducted on the species with the most genes and all hits to members of its genus were masked out. The remaining species were subjected to the usual analysis and any additional LGT genes were included in the analysis of the genus. In cases such as *Sulfolobus islandicus *where multiple strains have been sequenced a similar masking was performed. The donor species were assigned according to the best hit as matched by BLAST and a web search was then conducted to identify whether the donor species was a known halophile or thermophile respectively. Genes were assigned to COGs based upon the NCBI annotation. All additional analysis was performed using home-written scripts in Perl and/or Python. The scripts are available upon request. For the purposes of our statistical analysis the values of the non-LGT gene pool were taken as representative of the taxon as a whole. We then used a chi square test to assess the likelihood of the LGT genes originating in the same population.

### Phylogenetic Tree Building

The phylogenetic trees included in the supplemental material were constructed using the online tools available in association with the KEGG database. For each gene with a BLAST score of over 500, its twenty closest homologues were selected. The CLUSTALW tool was then used to create an alignment, and an unrooted neighbor joining tree was constructed. The phylogenetic trees were inspected manually for indicators of LGT directionality. For the phylogenetic trees depicted in Figures 1 and 2, LGT genes from both the Thermoprotei and Halobacteria were pooled into three pools depending on BLAST bit score. Five genes at random were chosen from each pool and the top 14 homologues from distinct genera were selected from the KEGG database. The CLUSTALW tool was again used to create an alignment. The alignments were then loaded into PHYLIP and trees were constructed with 100 bootstraps and the mean-least-squared method [39].

## Description of Additional Data Files

The following additional data is available with the online version of this paper. Additional file 1 is a collection of phylogenetic trees representing all LGT events with BLAST scores greater than 500 for both the Thermoprotei and the Halobacteria. Additional file 2 is a table listing the percentage of LGT events that are intra-environmental given various BLAST cutoff values. Additional data file 3 is a table listing the 22 "long distance" LGT events identified in the assembled fosmid sequences.

## Abbreviations

LGT: Lateral Gene Transfer; COGs: Clusters of Orthologous Groups of proteins.

## Authors' contributions

MR performed the bioinformatic analysis and laboratory work and drafted the manuscript. JS and CH jointly oversaw this study and aided in the revising process. AO provided expert guidance and also assisted in the editing of this manuscript. All authors have read and approved of the manuscript.

## Supplementary Material

Additional file 1 **Collection of phylogenetic trees for Thermoprotei and Halobacteria LGT genes with strong matches**. Trees for all LGT genes with BLAST scores greater than 500 in both the Thermoprotei and Halobacteria. The KEGG database three letter genome code is given before the colon and can be found here http://www.genome.jp/kegg/catalog/org_list.html. The corresponding gene locus tags are provided after the colon. Click here for file

Additional file 2 **Table of intra-environmental LGT proportions**. Table providing the proportion of LGT events originating from a donor inhabiting a similar environment for a spectrum of BLAST cutoff percentages. Click here for file

Additional file 3 **Fosmid LGT genes**. Table showing the 22 LGT events identified within environmental fosmid clones of the Dead Sea. Click here for file
