# Supplementary figures and images for: Differences in lateral gene transfer in hypersaline versus thermal environments
Source: BMC Evol Biol. 2011 Jul 8;11:199. doi: 10.1186/1471-2148-11-199 (PMC3236060; doi:10.1186/1471-2148-11-199)

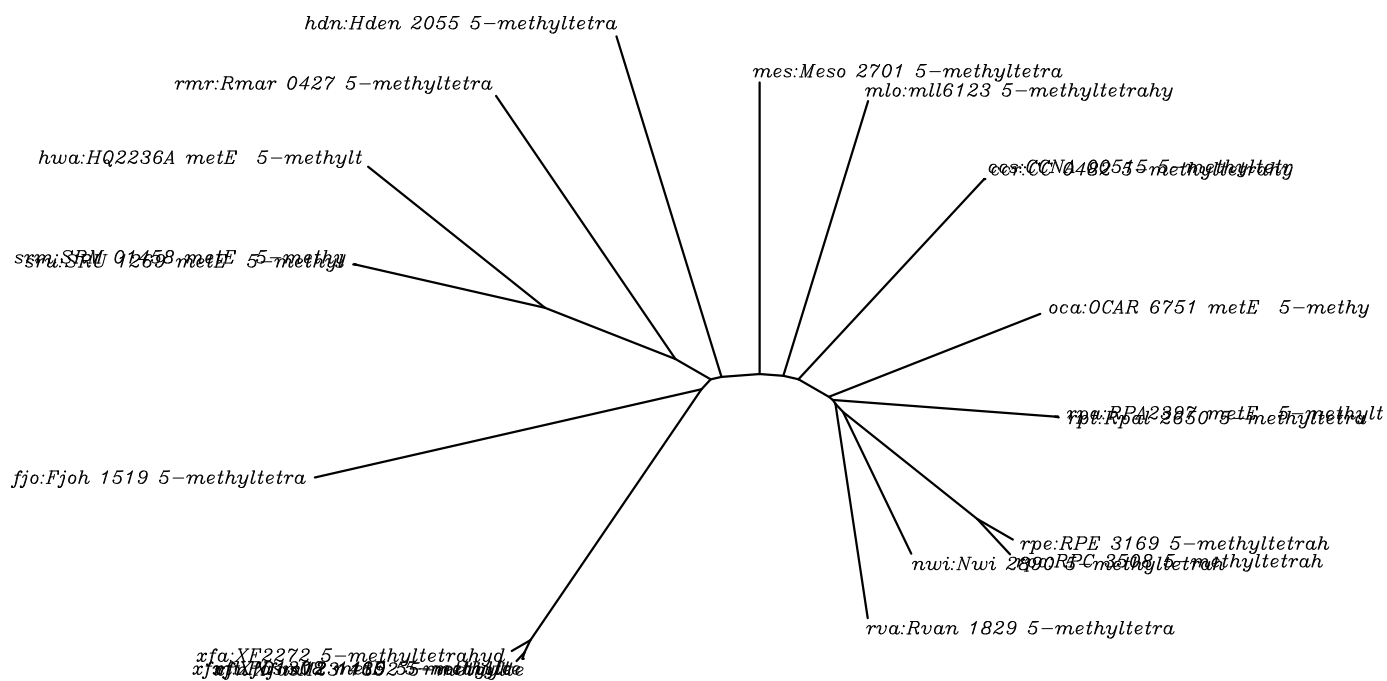

Supplement: Additional file 1 — Collection of phylogenetic trees for Thermoprotei and Halobacteria LGT genes with strong matches. Trees for all LGT genes with BLAST scores greater than 500 in both the Thermoprotei and Halobacteria. The KEGG database three letter genome code is given before the colon and can be found here http://www.genome.jp/kegg/catalog/org_list.html. The corresponding gene locus tags are provided after the colon. [file 1471-2148-11-199-S1.GZ › Trees/Htree1.pdf]

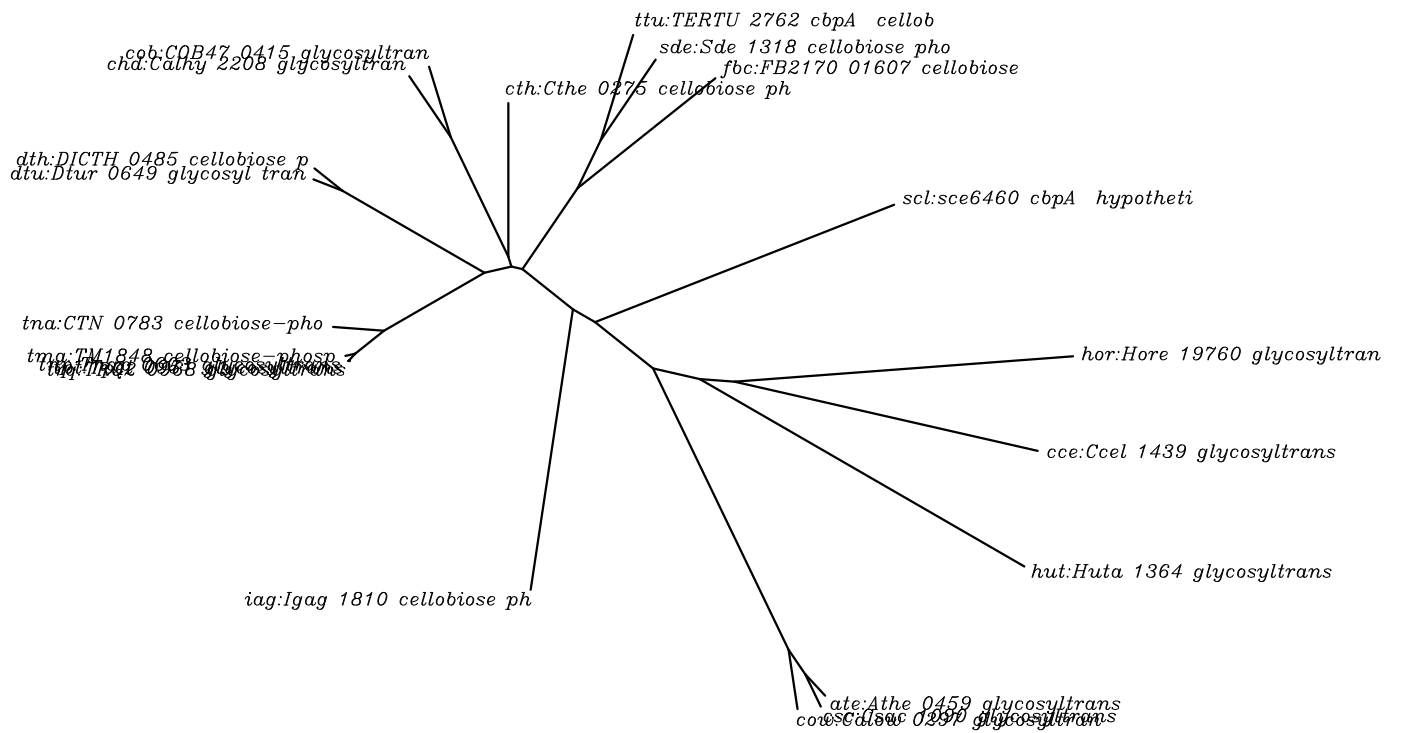

Supplement: Additional file 1 — Collection of phylogenetic trees for Thermoprotei and Halobacteria LGT genes with strong matches. Trees for all LGT genes with BLAST scores greater than 500 in both the Thermoprotei and Halobacteria. The KEGG database three letter genome code is given before the colon and can be found here http://www.genome.jp/kegg/catalog/org_list.html. The corresponding gene locus tags are provided after the colon. [file 1471-2148-11-199-S1.GZ › Trees/Htree10.pdf]

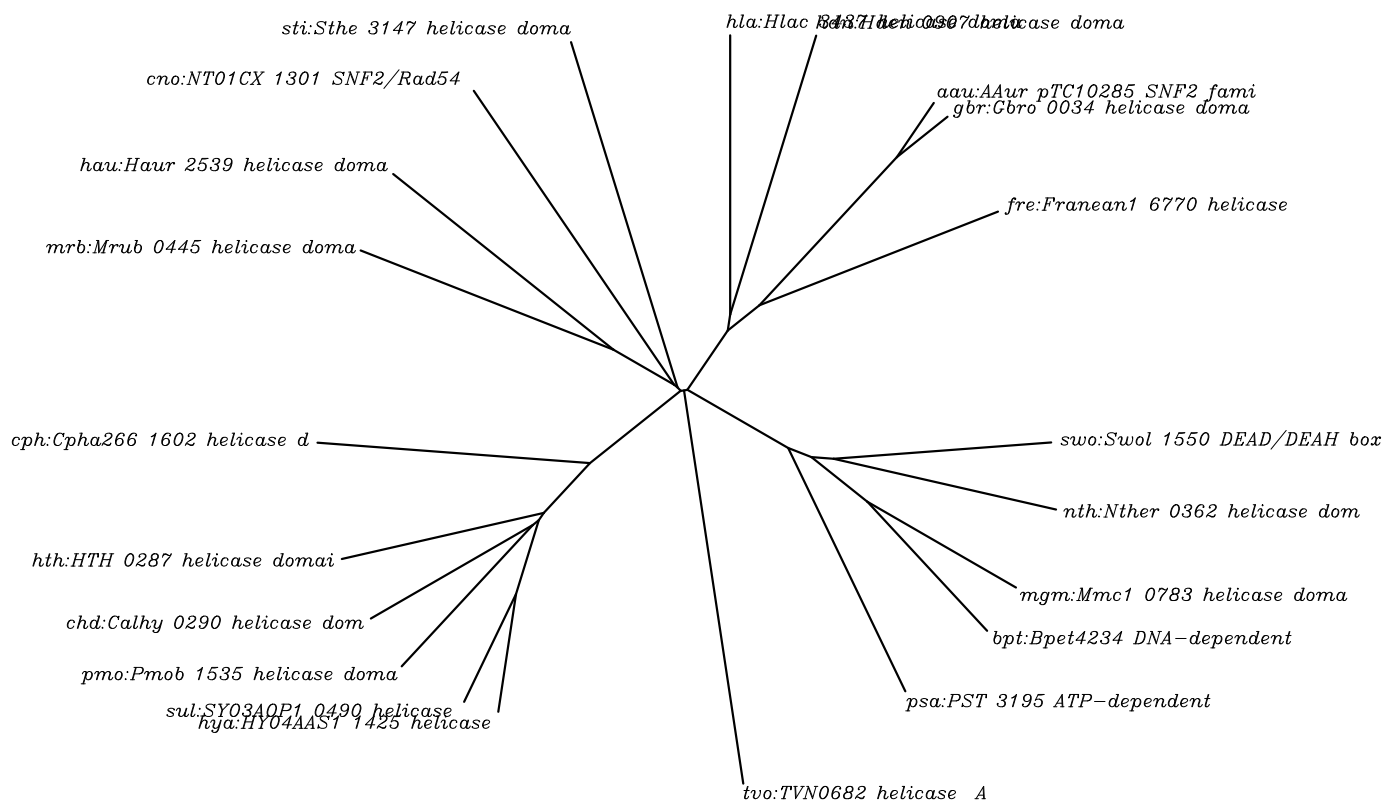

Supplement: Additional file 1 — Collection of phylogenetic trees for Thermoprotei and Halobacteria LGT genes with strong matches. Trees for all LGT genes with BLAST scores greater than 500 in both the Thermoprotei and Halobacteria. The KEGG database three letter genome code is given before the colon and can be found here http://www.genome.jp/kegg/catalog/org_list.html. The corresponding gene locus tags are provided after the colon. [file 1471-2148-11-199-S1.GZ › Trees/Htree11.pdf]

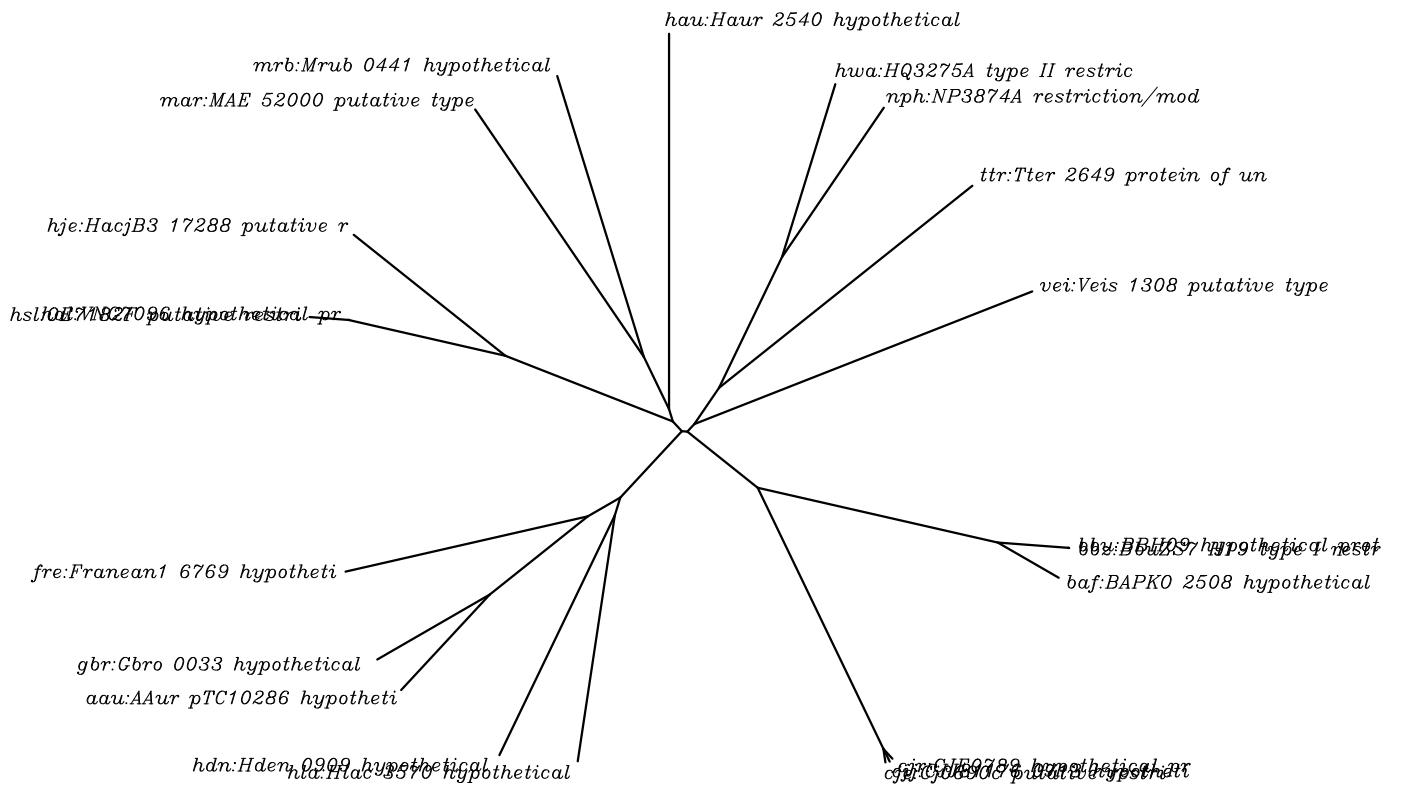

Supplement: Additional file 1 — Collection of phylogenetic trees for Thermoprotei and Halobacteria LGT genes with strong matches. Trees for all LGT genes with BLAST scores greater than 500 in both the Thermoprotei and Halobacteria. The KEGG database three letter genome code is given before the colon and can be found here http://www.genome.jp/kegg/catalog/org_list.html. The corresponding gene locus tags are provided after the colon. [file 1471-2148-11-199-S1.GZ › Trees/Htree12.pdf]

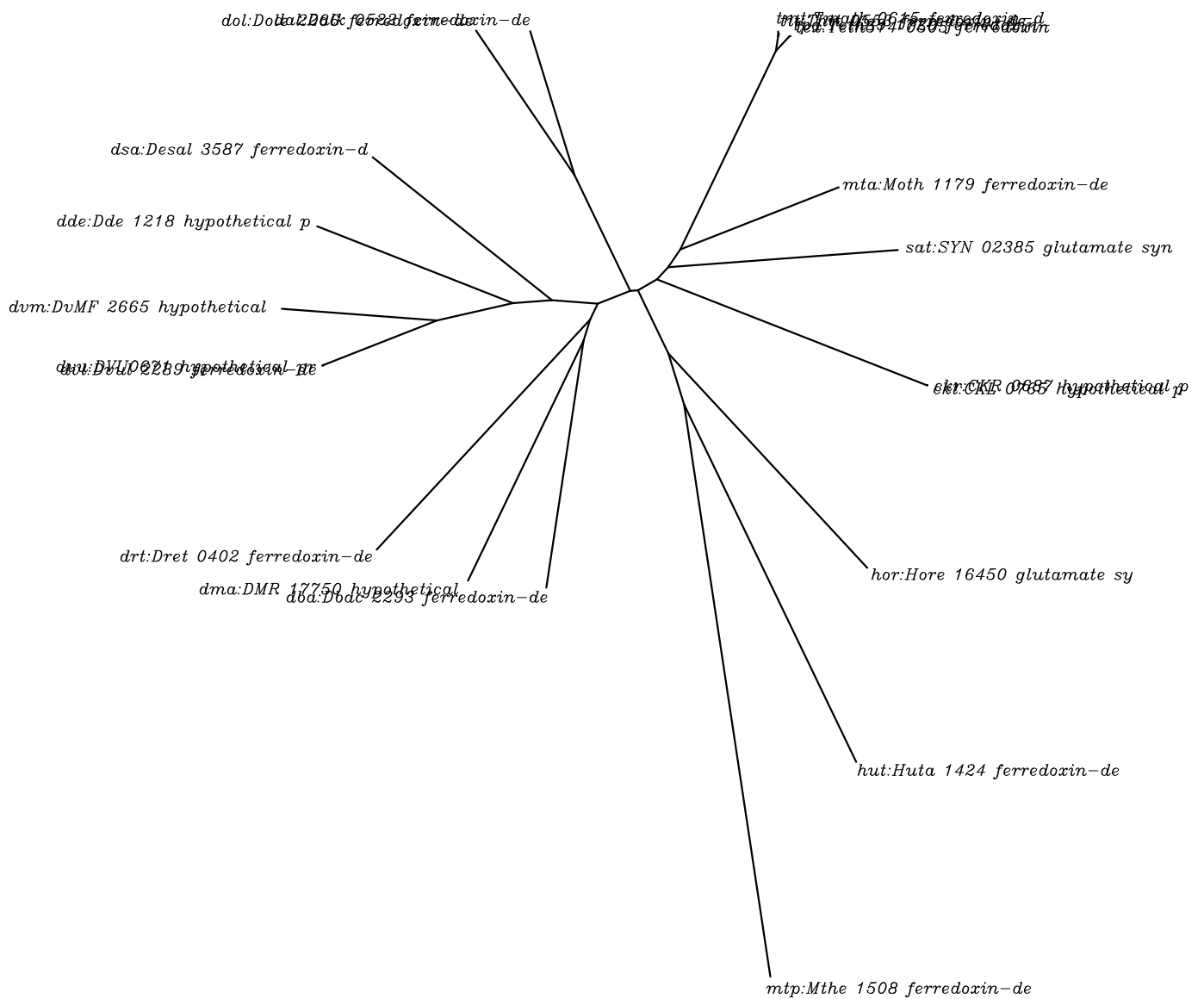

Supplement: Additional file 1 — Collection of phylogenetic trees for Thermoprotei and Halobacteria LGT genes with strong matches. Trees for all LGT genes with BLAST scores greater than 500 in both the Thermoprotei and Halobacteria. The KEGG database three letter genome code is given before the colon and can be found here http://www.genome.jp/kegg/catalog/org_list.html. The corresponding gene locus tags are provided after the colon. [file 1471-2148-11-199-S1.GZ › Trees/Htree13.pdf]

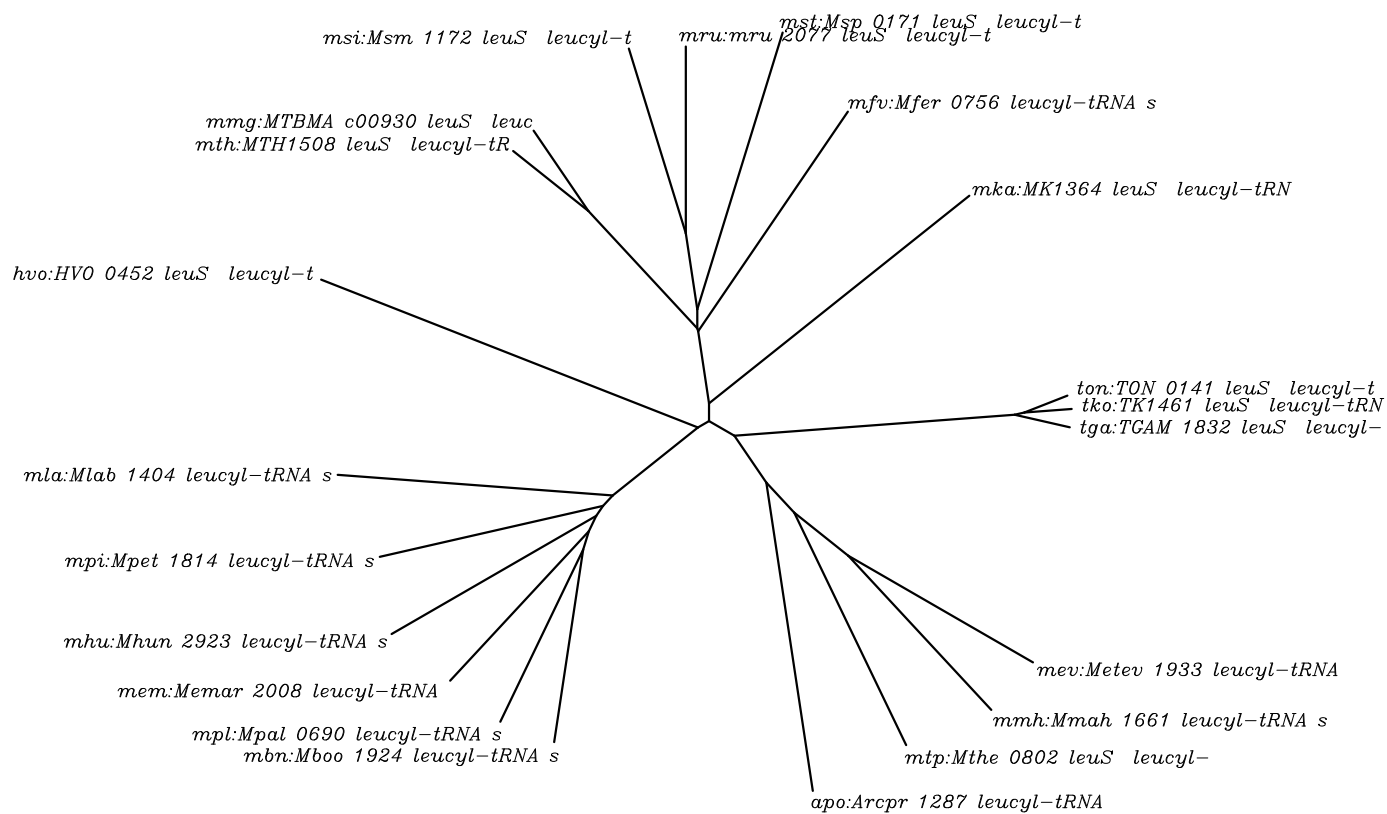

Supplement: Additional file 1 — Collection of phylogenetic trees for Thermoprotei and Halobacteria LGT genes with strong matches. Trees for all LGT genes with BLAST scores greater than 500 in both the Thermoprotei and Halobacteria. The KEGG database three letter genome code is given before the colon and can be found here http://www.genome.jp/kegg/catalog/org_list.html. The corresponding gene locus tags are provided after the colon. [file 1471-2148-11-199-S1.GZ › Trees/Htree14.pdf]

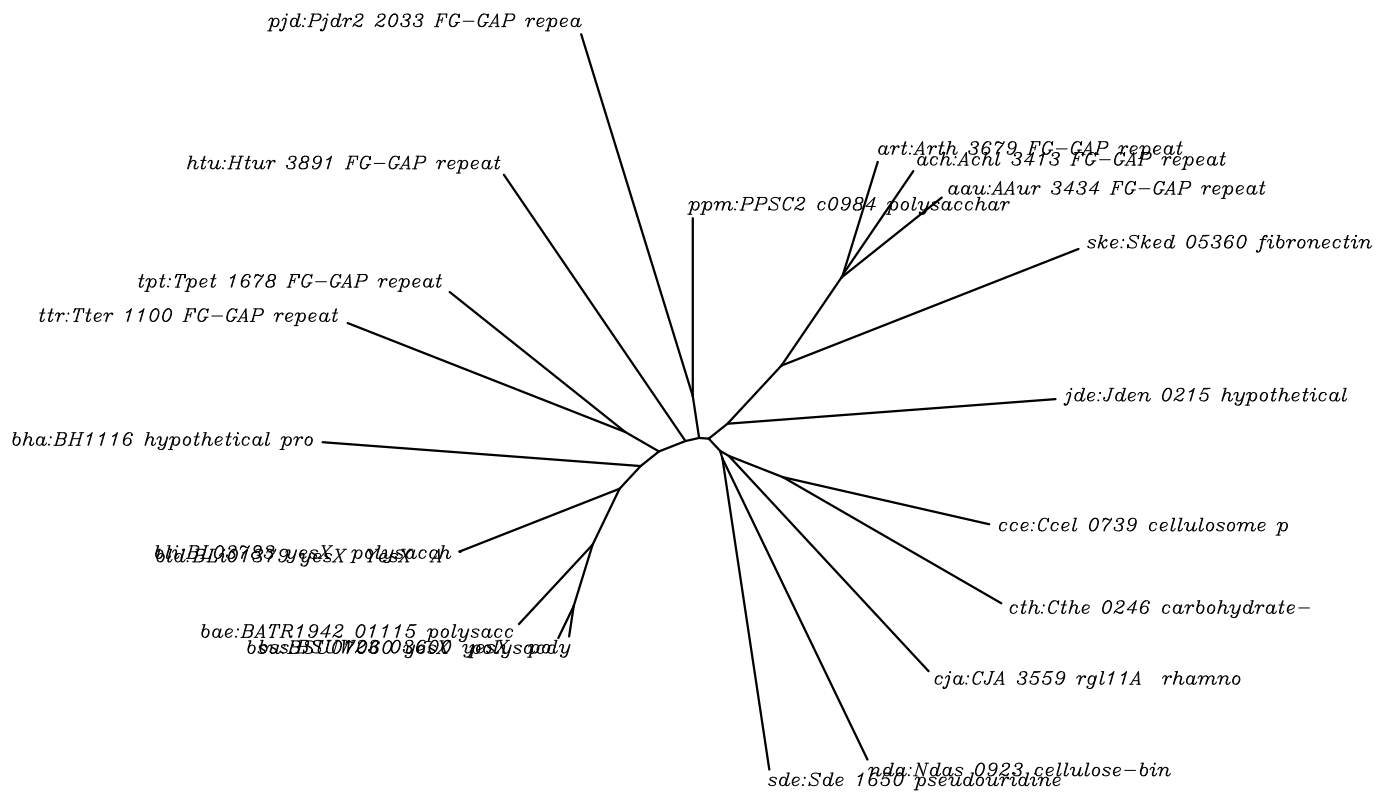

Supplement: Additional file 1 — Collection of phylogenetic trees for Thermoprotei and Halobacteria LGT genes with strong matches. Trees for all LGT genes with BLAST scores greater than 500 in both the Thermoprotei and Halobacteria. The KEGG database three letter genome code is given before the colon and can be found here http://www.genome.jp/kegg/catalog/org_list.html. The corresponding gene locus tags are provided after the colon. [file 1471-2148-11-199-S1.GZ › Trees/Htree15.pdf]

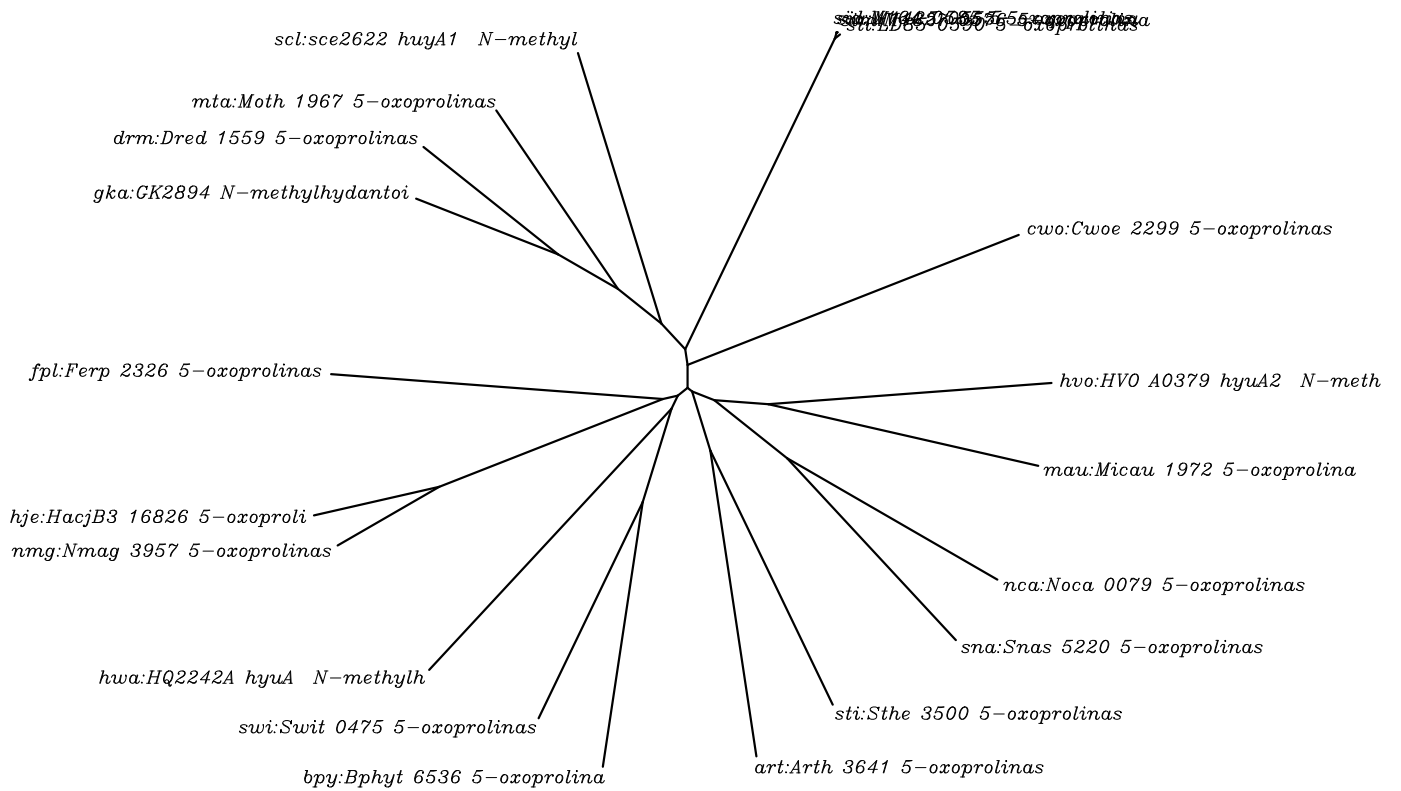

Supplement: Additional file 1 — Collection of phylogenetic trees for Thermoprotei and Halobacteria LGT genes with strong matches. Trees for all LGT genes with BLAST scores greater than 500 in both the Thermoprotei and Halobacteria. The KEGG database three letter genome code is given before the colon and can be found here http://www.genome.jp/kegg/catalog/org_list.html. The corresponding gene locus tags are provided after the colon. [file 1471-2148-11-199-S1.GZ › Trees/Htree16.pdf]

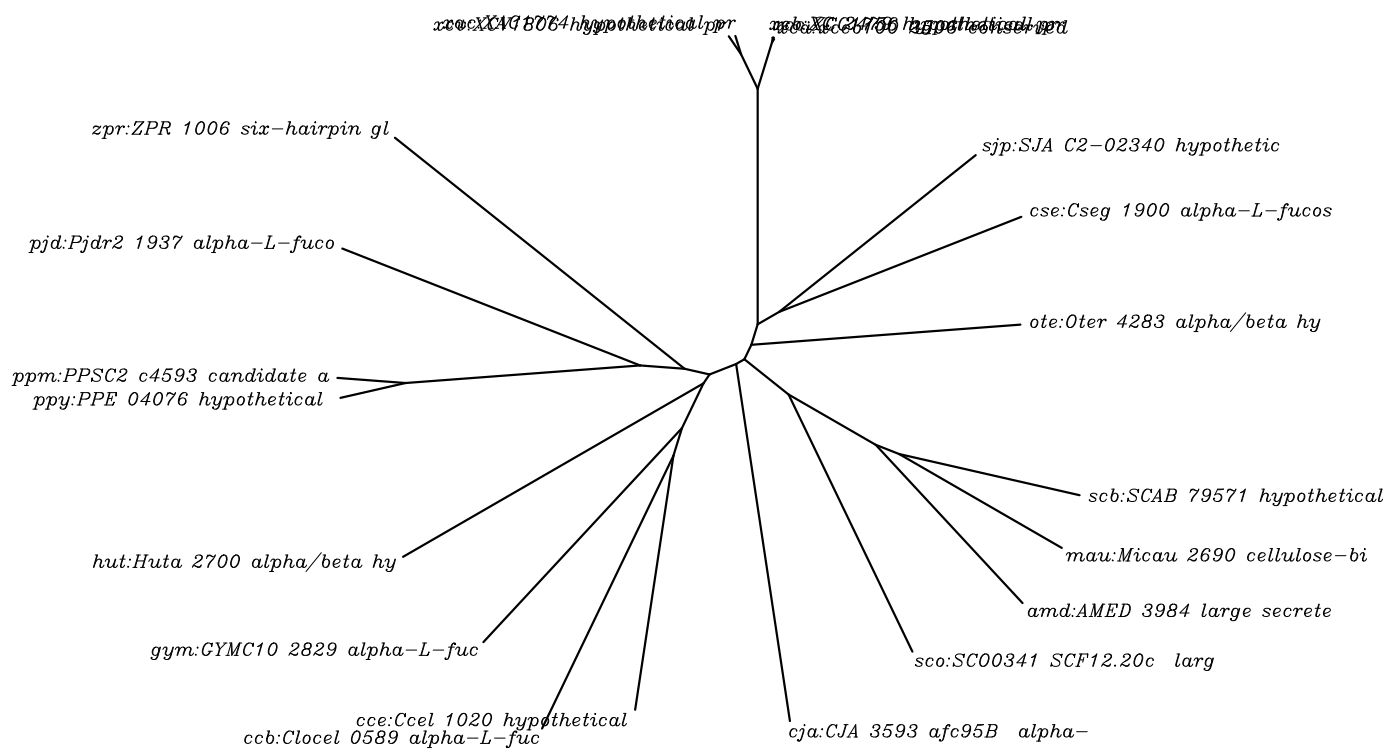

Supplement: Additional file 1 — Collection of phylogenetic trees for Thermoprotei and Halobacteria LGT genes with strong matches. Trees for all LGT genes with BLAST scores greater than 500 in both the Thermoprotei and Halobacteria. The KEGG database three letter genome code is given before the colon and can be found here http://www.genome.jp/kegg/catalog/org_list.html. The corresponding gene locus tags are provided after the colon. [file 1471-2148-11-199-S1.GZ › Trees/Htree17.pdf]

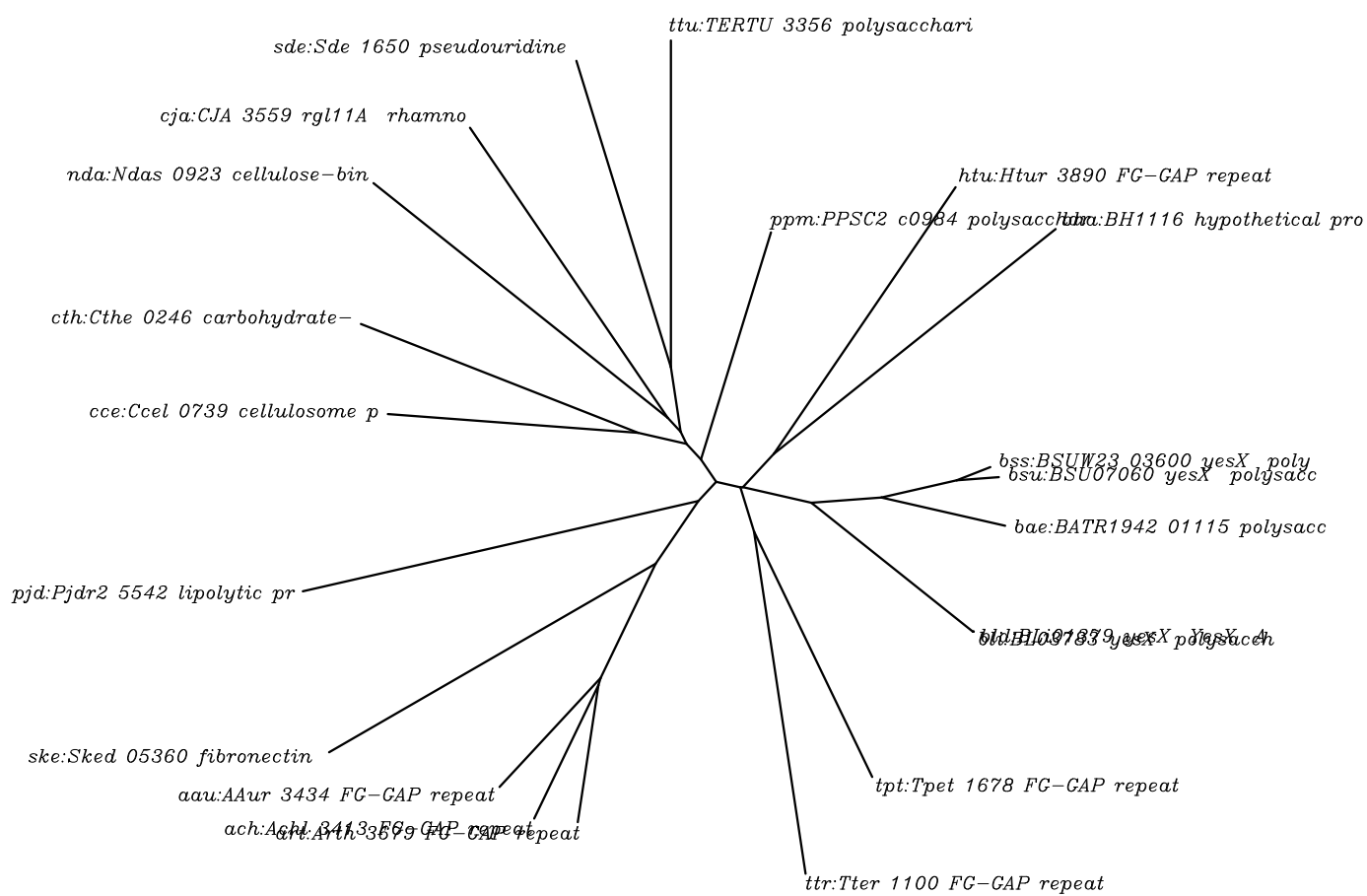

Supplement: Additional file 1 — Collection of phylogenetic trees for Thermoprotei and Halobacteria LGT genes with strong matches. Trees for all LGT genes with BLAST scores greater than 500 in both the Thermoprotei and Halobacteria. The KEGG database three letter genome code is given before the colon and can be found here http://www.genome.jp/kegg/catalog/org_list.html. The corresponding gene locus tags are provided after the colon. [file 1471-2148-11-199-S1.GZ › Trees/Htree18.pdf]

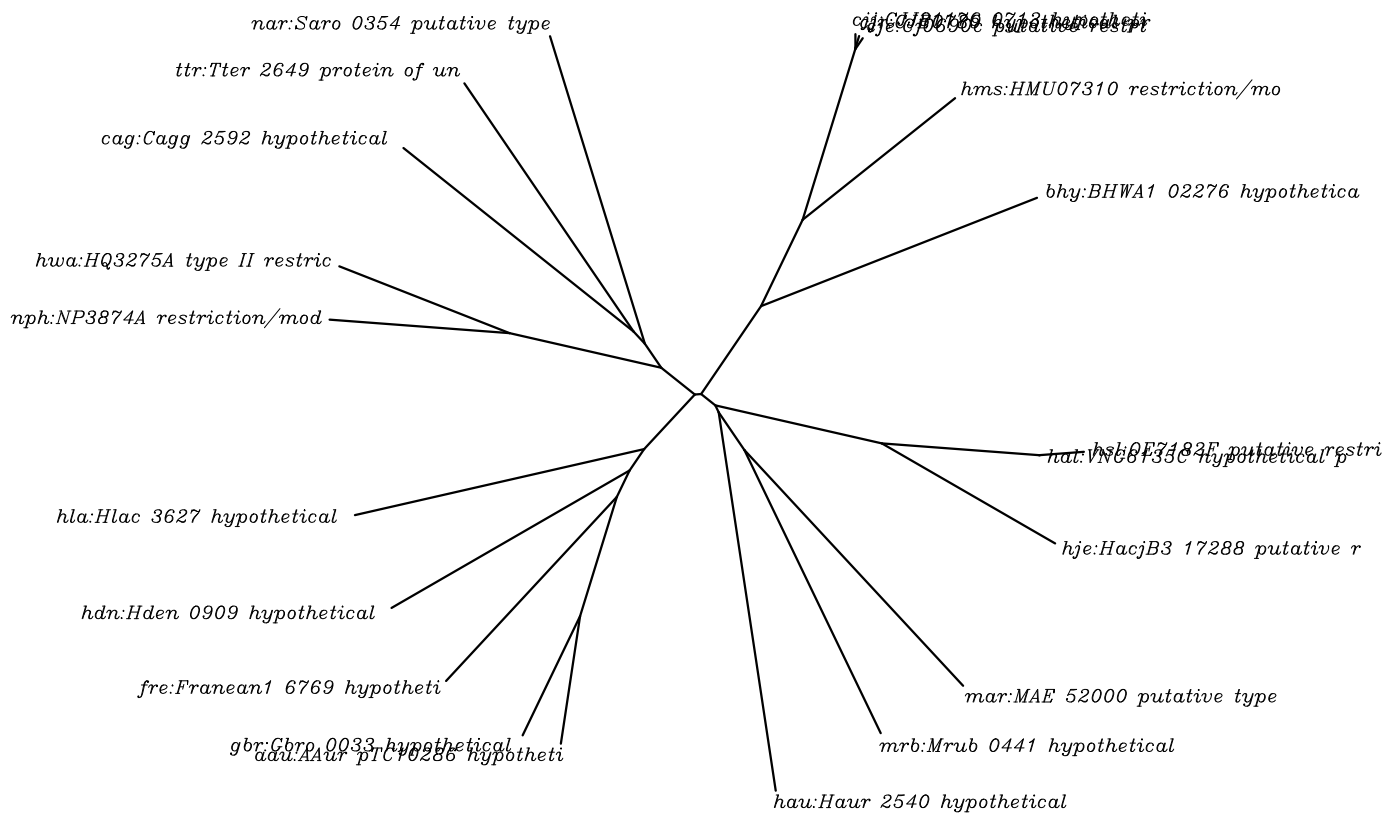

Supplement: Additional file 1 — Collection of phylogenetic trees for Thermoprotei and Halobacteria LGT genes with strong matches. Trees for all LGT genes with BLAST scores greater than 500 in both the Thermoprotei and Halobacteria. The KEGG database three letter genome code is given before the colon and can be found here http://www.genome.jp/kegg/catalog/org_list.html. The corresponding gene locus tags are provided after the colon. [file 1471-2148-11-199-S1.GZ › Trees/Htree19.pdf]

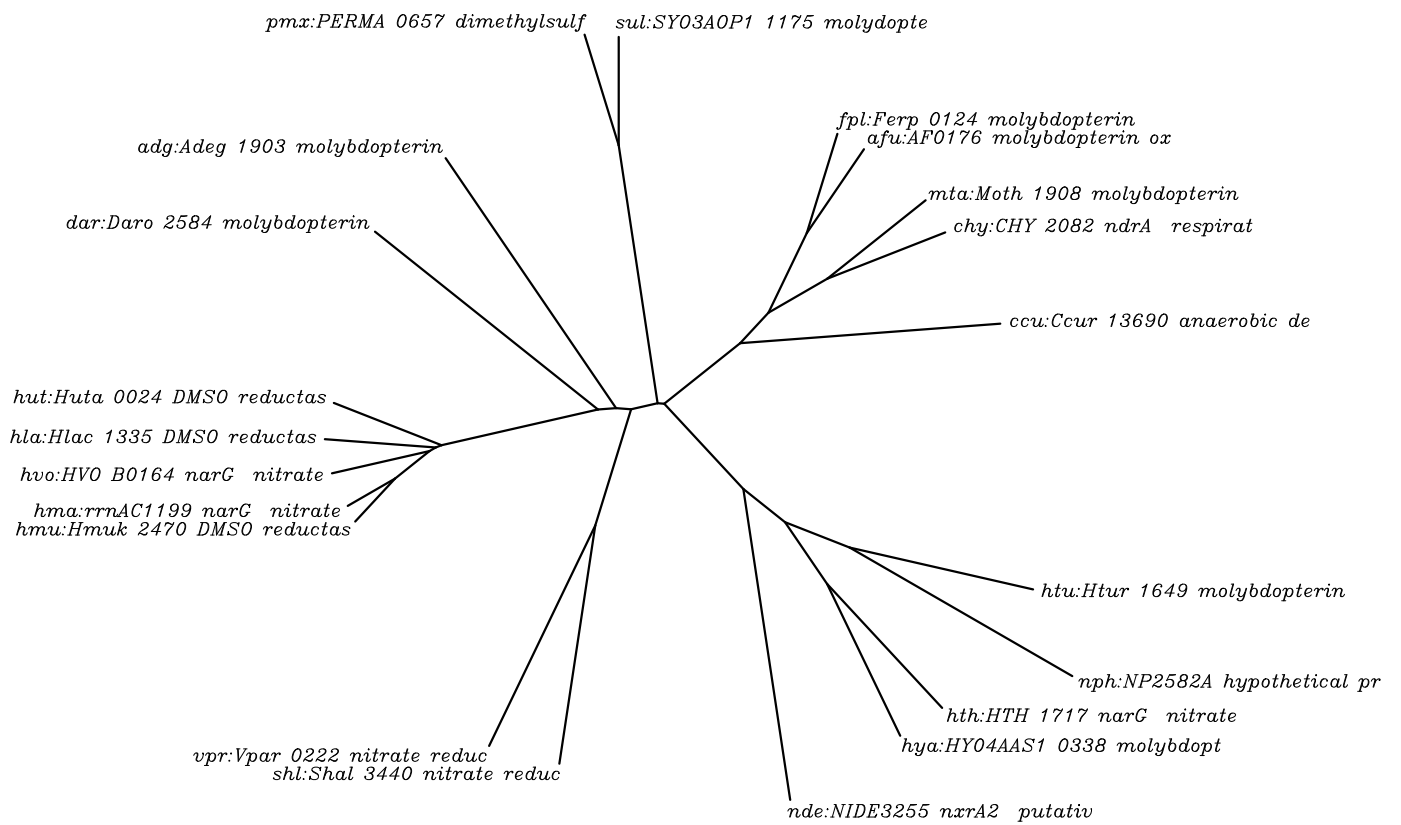

Supplement: Additional file 1 — Collection of phylogenetic trees for Thermoprotei and Halobacteria LGT genes with strong matches. Trees for all LGT genes with BLAST scores greater than 500 in both the Thermoprotei and Halobacteria. The KEGG database three letter genome code is given before the colon and can be found here http://www.genome.jp/kegg/catalog/org_list.html. The corresponding gene locus tags are provided after the colon. [file 1471-2148-11-199-S1.GZ › Trees/Htree2.pdf]

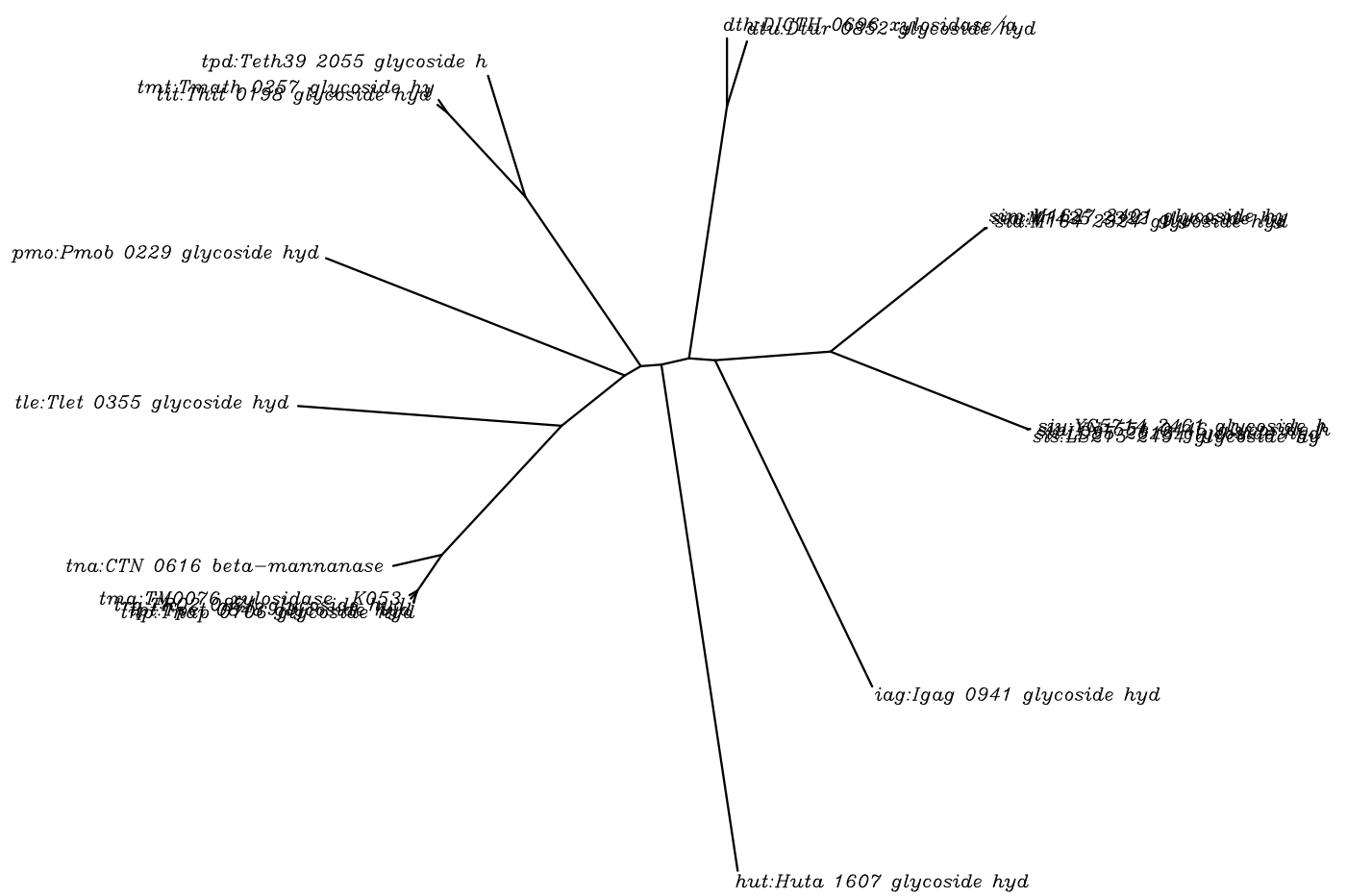

Supplement: Additional file 1 — Collection of phylogenetic trees for Thermoprotei and Halobacteria LGT genes with strong matches. Trees for all LGT genes with BLAST scores greater than 500 in both the Thermoprotei and Halobacteria. The KEGG database three letter genome code is given before the colon and can be found here http://www.genome.jp/kegg/catalog/org_list.html. The corresponding gene locus tags are provided after the colon. [file 1471-2148-11-199-S1.GZ › Trees/Htree20.pdf]

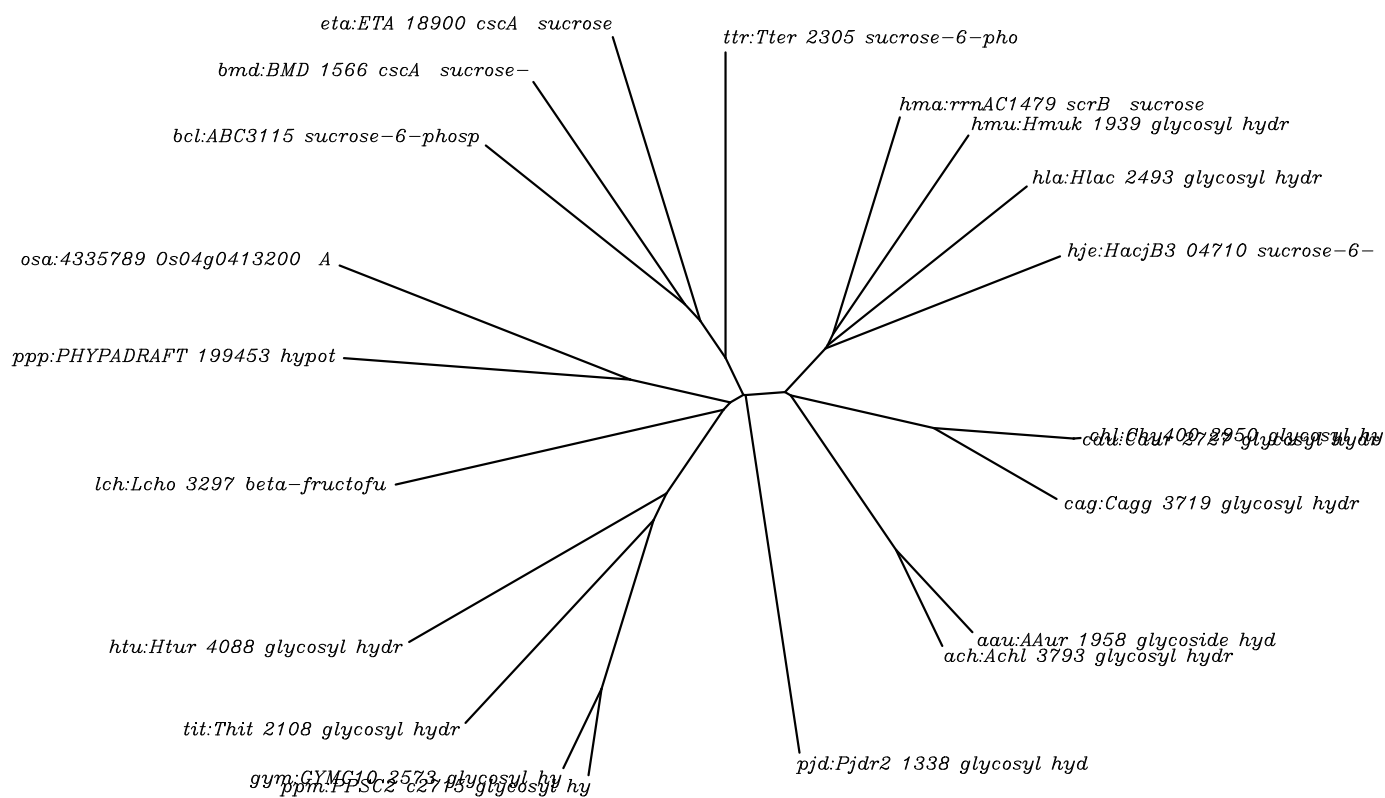

Supplement: Additional file 1 — Collection of phylogenetic trees for Thermoprotei and Halobacteria LGT genes with strong matches. Trees for all LGT genes with BLAST scores greater than 500 in both the Thermoprotei and Halobacteria. The KEGG database three letter genome code is given before the colon and can be found here http://www.genome.jp/kegg/catalog/org_list.html. The corresponding gene locus tags are provided after the colon. [file 1471-2148-11-199-S1.GZ › Trees/Htree21.pdf]

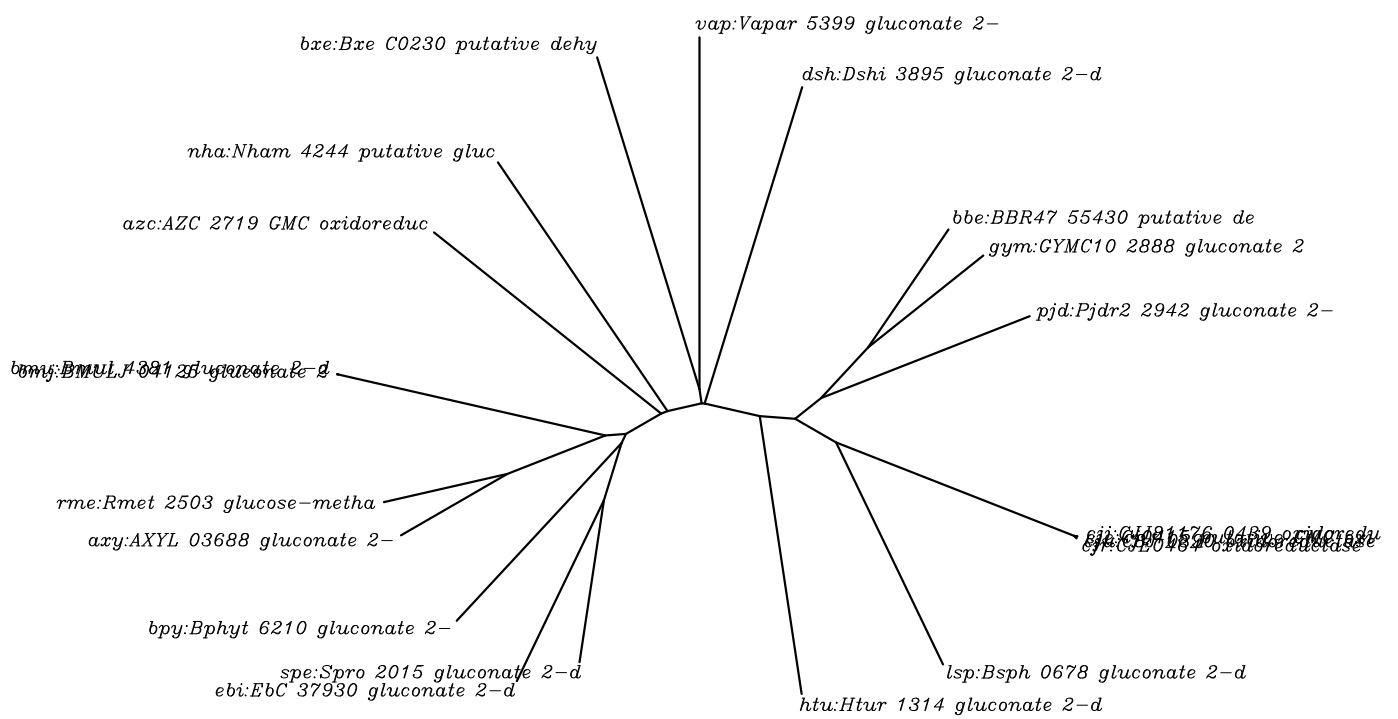

Supplement: Additional file 1 — Collection of phylogenetic trees for Thermoprotei and Halobacteria LGT genes with strong matches. Trees for all LGT genes with BLAST scores greater than 500 in both the Thermoprotei and Halobacteria. The KEGG database three letter genome code is given before the colon and can be found here http://www.genome.jp/kegg/catalog/org_list.html. The corresponding gene locus tags are provided after the colon. [file 1471-2148-11-199-S1.GZ › Trees/Htree22.pdf]

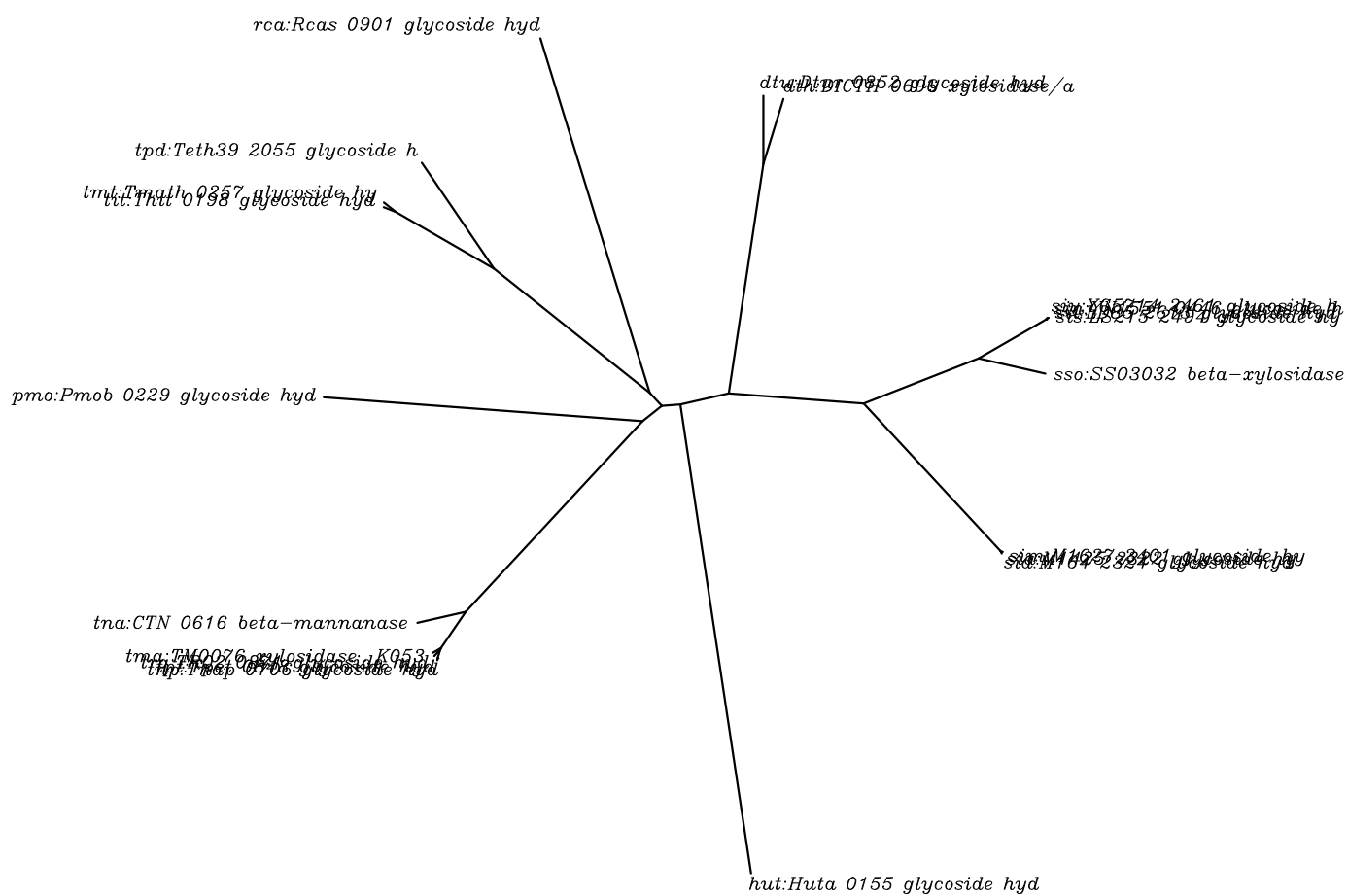

Supplement: Additional file 1 — Collection of phylogenetic trees for Thermoprotei and Halobacteria LGT genes with strong matches. Trees for all LGT genes with BLAST scores greater than 500 in both the Thermoprotei and Halobacteria. The KEGG database three letter genome code is given before the colon and can be found here http://www.genome.jp/kegg/catalog/org_list.html. The corresponding gene locus tags are provided after the colon. [file 1471-2148-11-199-S1.GZ › Trees/Htree23.pdf]

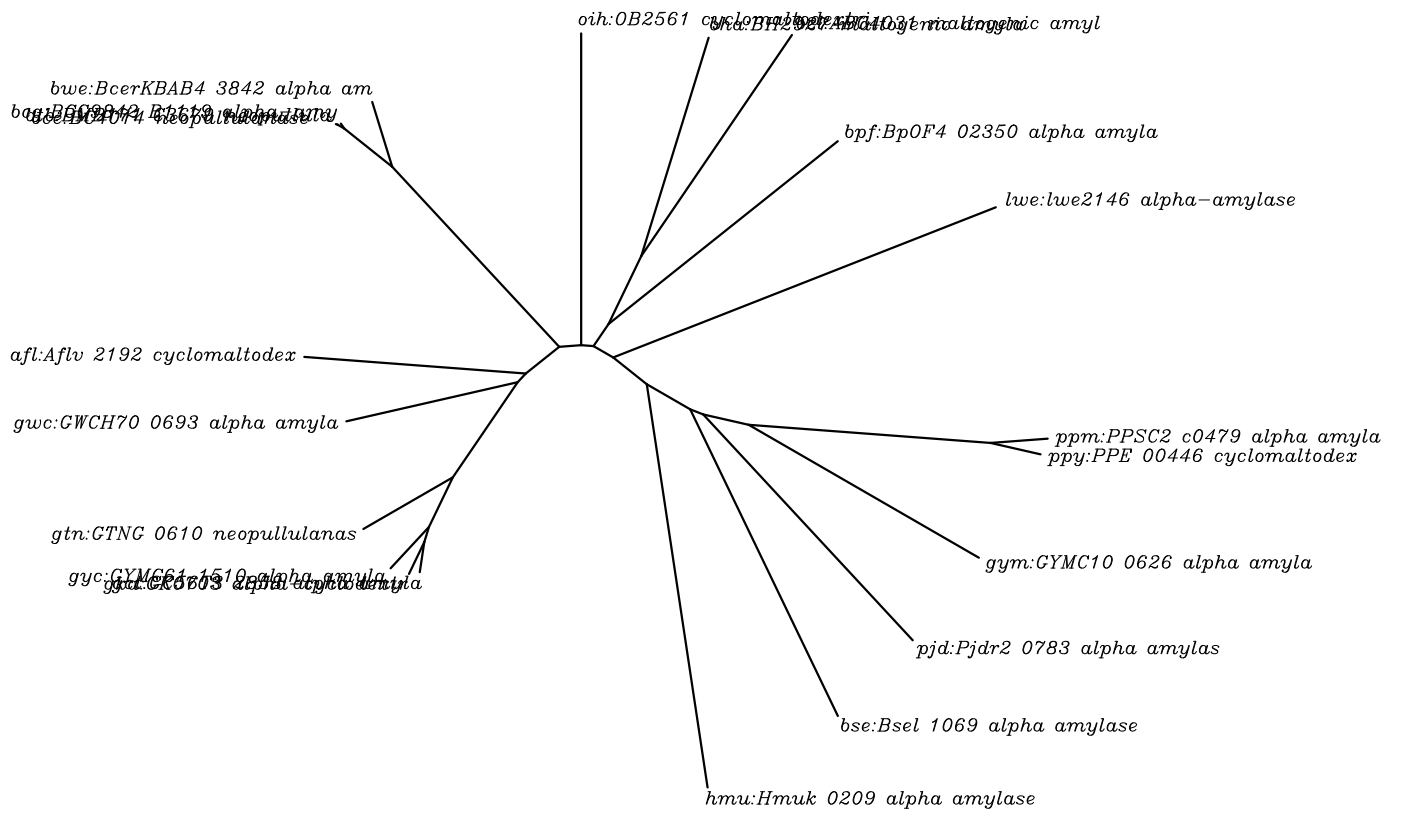

Supplement: Additional file 1 — Collection of phylogenetic trees for Thermoprotei and Halobacteria LGT genes with strong matches. Trees for all LGT genes with BLAST scores greater than 500 in both the Thermoprotei and Halobacteria. The KEGG database three letter genome code is given before the colon and can be found here http://www.genome.jp/kegg/catalog/org_list.html. The corresponding gene locus tags are provided after the colon. [file 1471-2148-11-199-S1.GZ › Trees/Htree24.pdf]

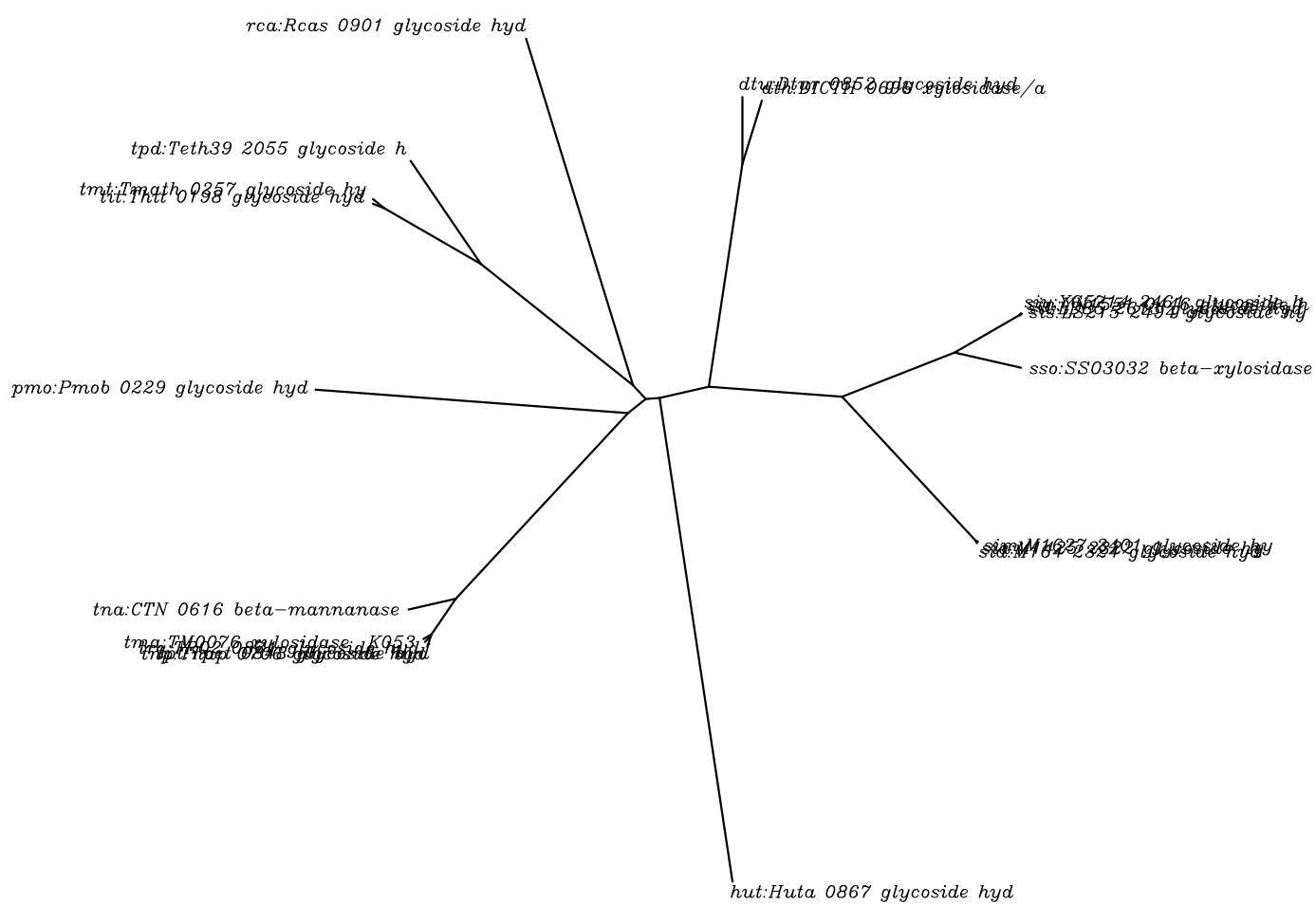

Supplement: Additional file 1 — Collection of phylogenetic trees for Thermoprotei and Halobacteria LGT genes with strong matches. Trees for all LGT genes with BLAST scores greater than 500 in both the Thermoprotei and Halobacteria. The KEGG database three letter genome code is given before the colon and can be found here http://www.genome.jp/kegg/catalog/org_list.html. The corresponding gene locus tags are provided after the colon. [file 1471-2148-11-199-S1.GZ › Trees/htree25.pdf]

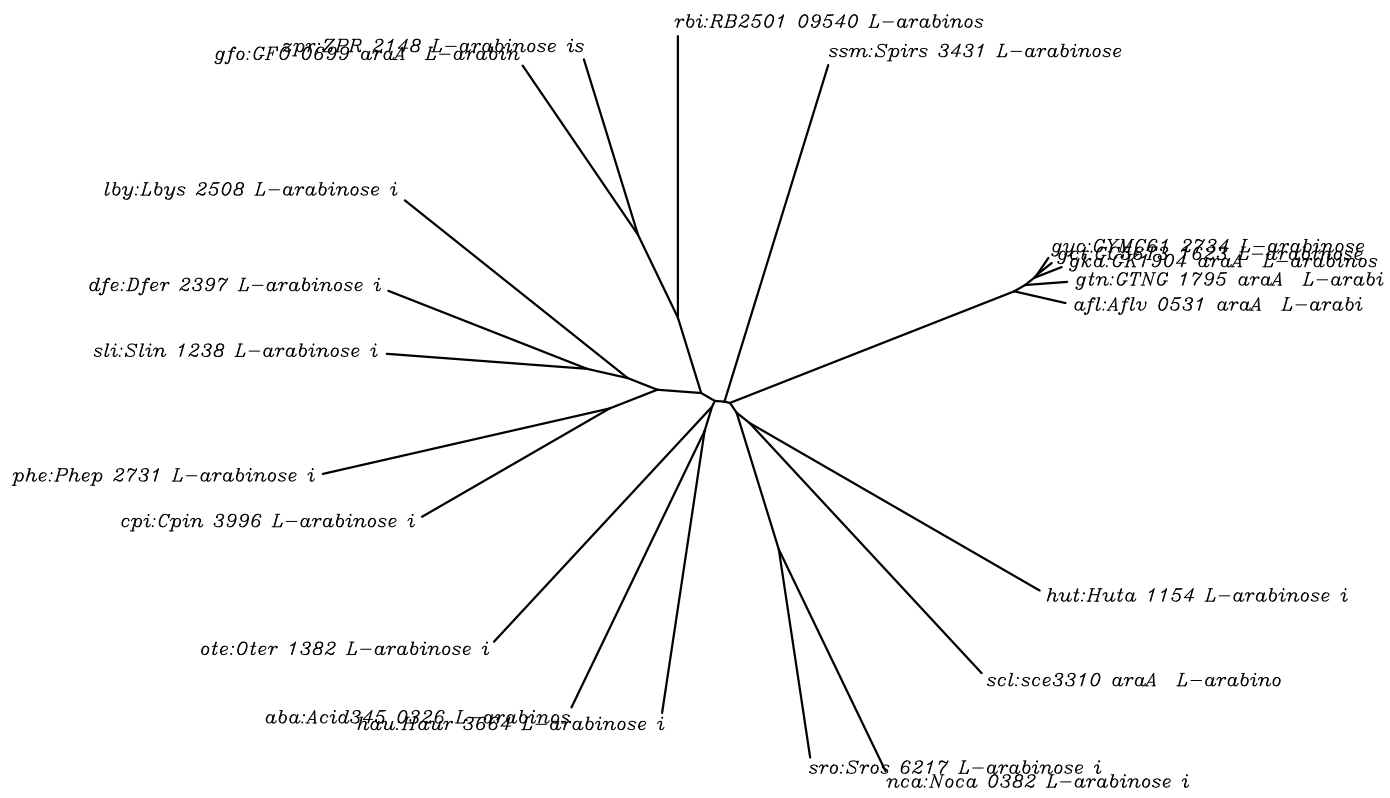

Supplement: Additional file 1 — Collection of phylogenetic trees for Thermoprotei and Halobacteria LGT genes with strong matches. Trees for all LGT genes with BLAST scores greater than 500 in both the Thermoprotei and Halobacteria. The KEGG database three letter genome code is given before the colon and can be found here http://www.genome.jp/kegg/catalog/org_list.html. The corresponding gene locus tags are provided after the colon. [file 1471-2148-11-199-S1.GZ › Trees/Htree26.pdf]

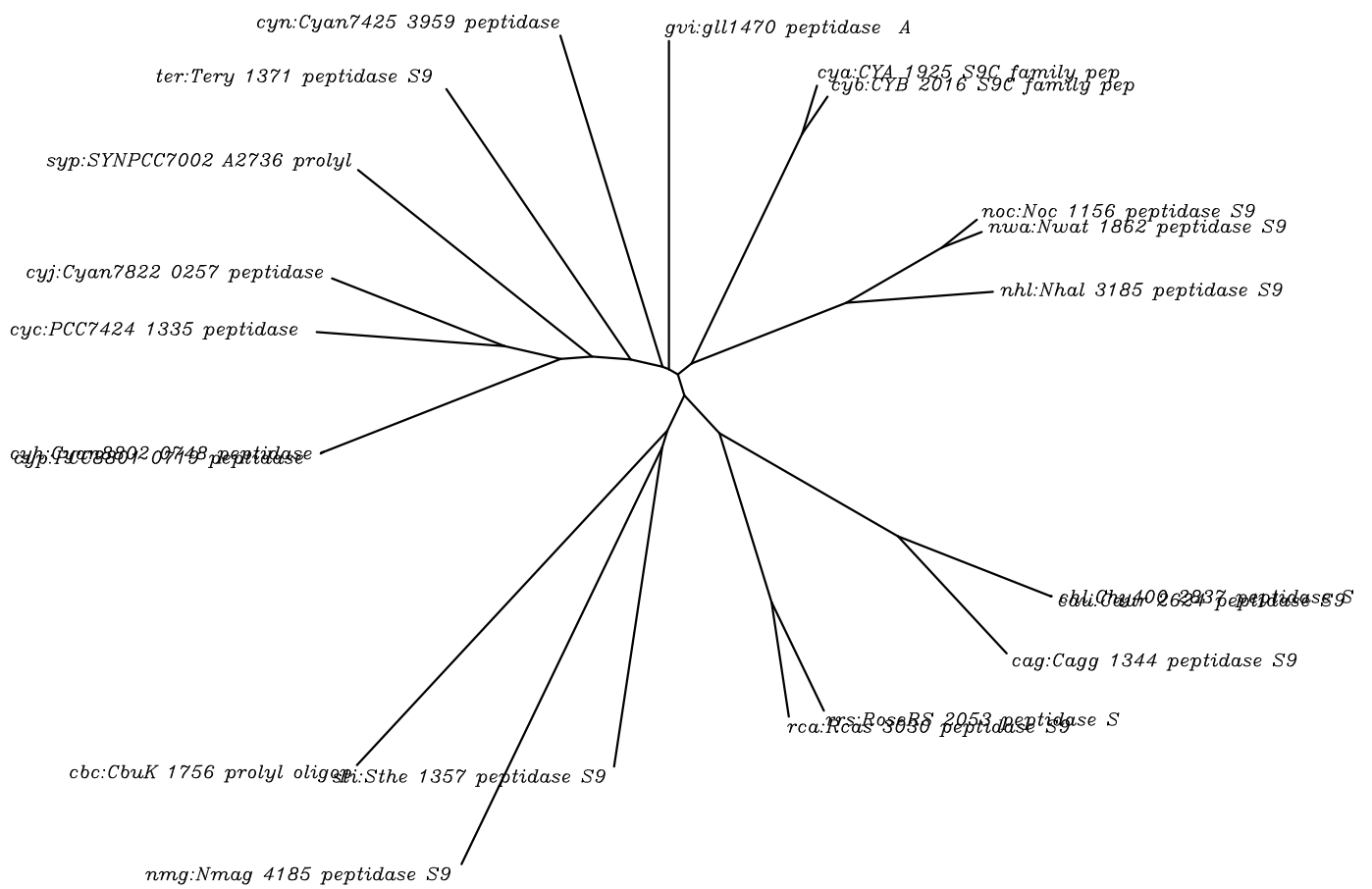

Supplement: Additional file 1 — Collection of phylogenetic trees for Thermoprotei and Halobacteria LGT genes with strong matches. Trees for all LGT genes with BLAST scores greater than 500 in both the Thermoprotei and Halobacteria. The KEGG database three letter genome code is given before the colon and can be found here http://www.genome.jp/kegg/catalog/org_list.html. The corresponding gene locus tags are provided after the colon. [file 1471-2148-11-199-S1.GZ › Trees/Htree27.pdf]

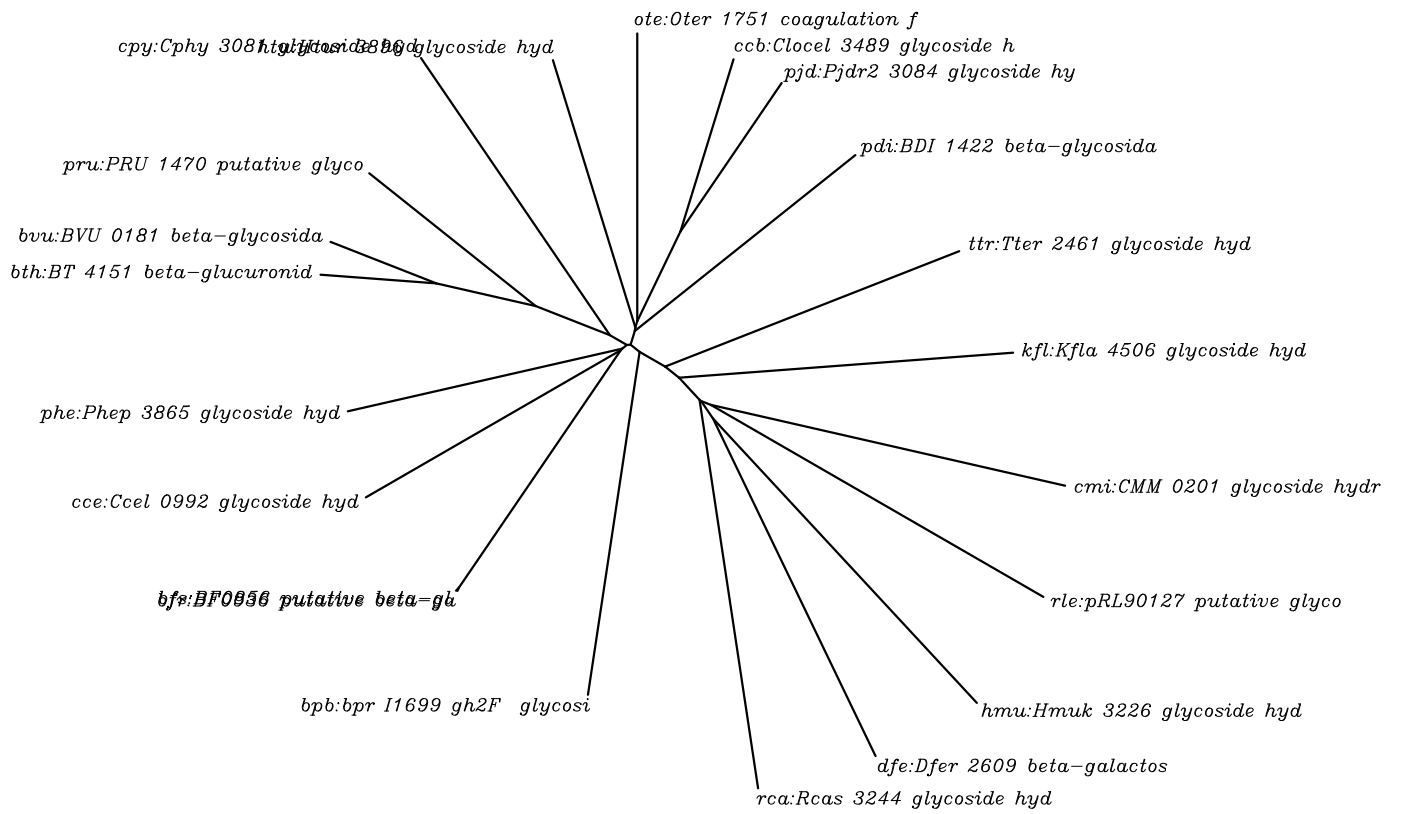

Supplement: Additional file 1 — Collection of phylogenetic trees for Thermoprotei and Halobacteria LGT genes with strong matches. Trees for all LGT genes with BLAST scores greater than 500 in both the Thermoprotei and Halobacteria. The KEGG database three letter genome code is given before the colon and can be found here http://www.genome.jp/kegg/catalog/org_list.html. The corresponding gene locus tags are provided after the colon. [file 1471-2148-11-199-S1.GZ › Trees/Htree28.pdf]

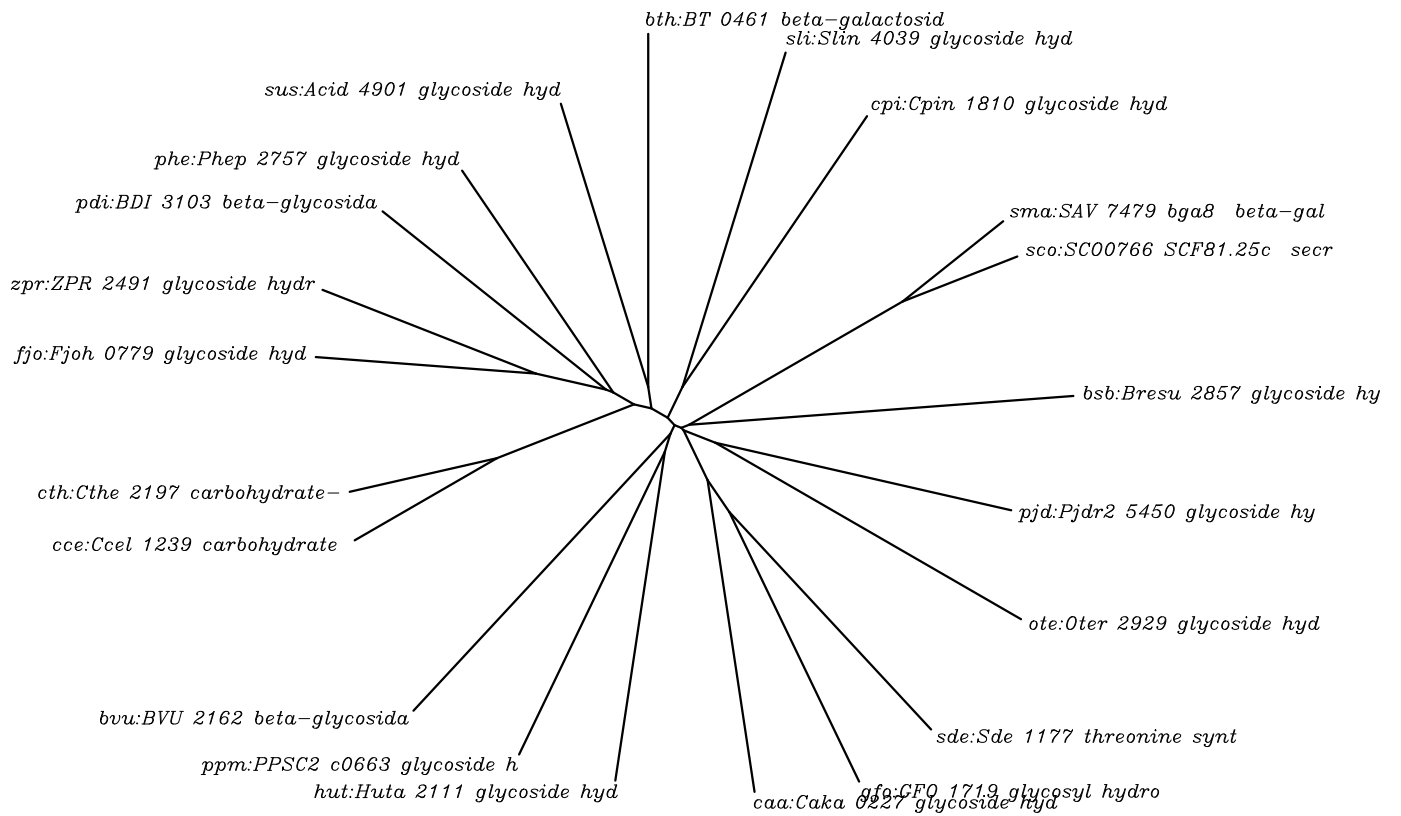

Supplement: Additional file 1 — Collection of phylogenetic trees for Thermoprotei and Halobacteria LGT genes with strong matches. Trees for all LGT genes with BLAST scores greater than 500 in both the Thermoprotei and Halobacteria. The KEGG database three letter genome code is given before the colon and can be found here http://www.genome.jp/kegg/catalog/org_list.html. The corresponding gene locus tags are provided after the colon. [file 1471-2148-11-199-S1.GZ › Trees/Htree29.pdf]

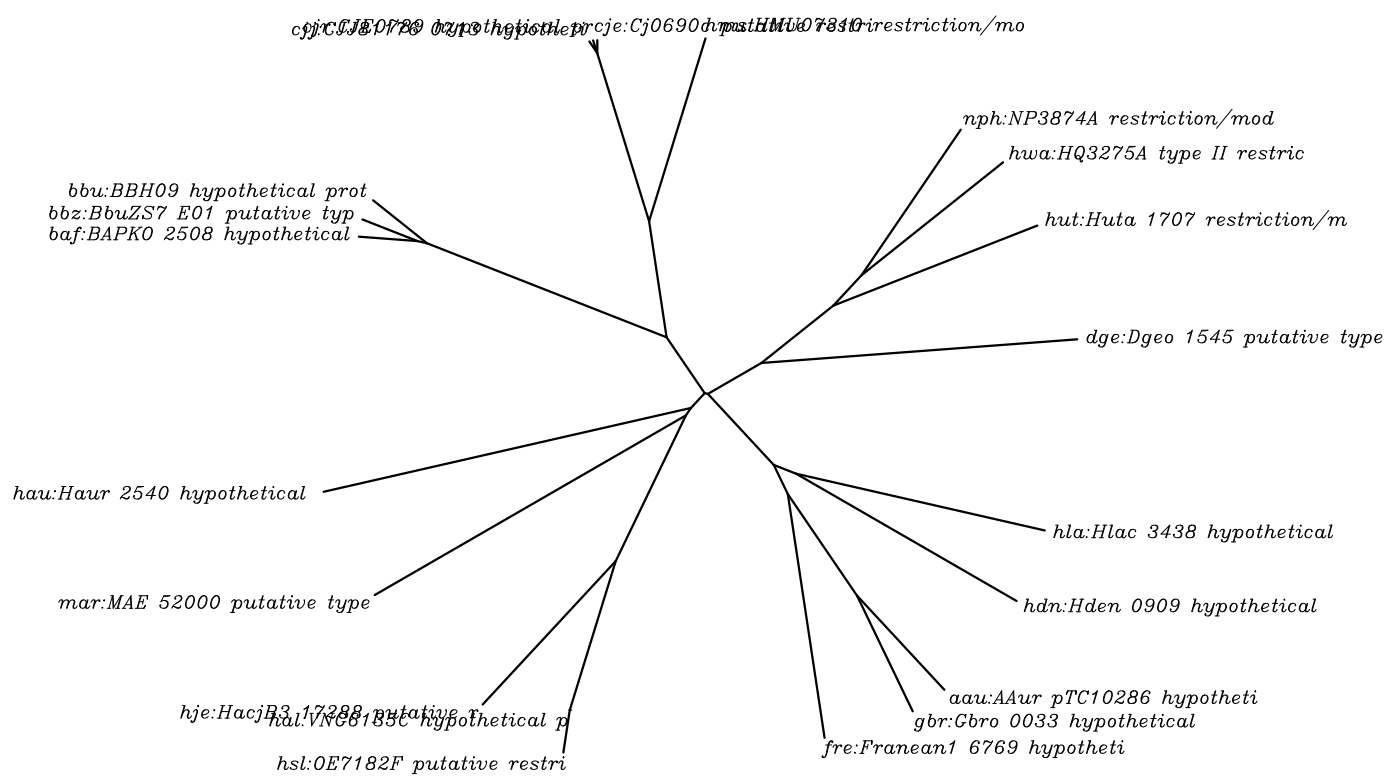

Supplement: Additional file 1 — Collection of phylogenetic trees for Thermoprotei and Halobacteria LGT genes with strong matches. Trees for all LGT genes with BLAST scores greater than 500 in both the Thermoprotei and Halobacteria. The KEGG database three letter genome code is given before the colon and can be found here http://www.genome.jp/kegg/catalog/org_list.html. The corresponding gene locus tags are provided after the colon. [file 1471-2148-11-199-S1.GZ › Trees/Htree3.pdf]

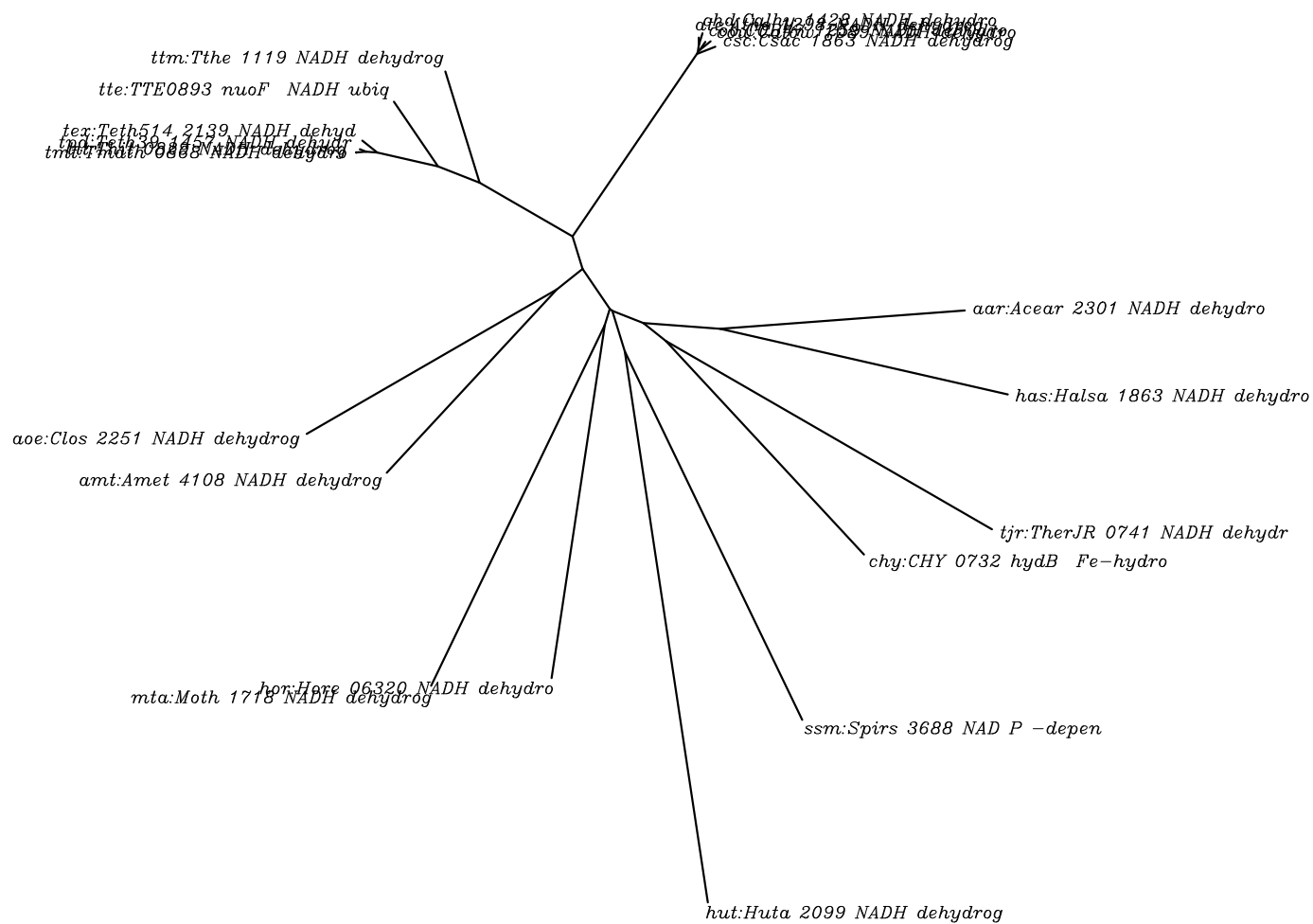

Supplement: Additional file 1 — Collection of phylogenetic trees for Thermoprotei and Halobacteria LGT genes with strong matches. Trees for all LGT genes with BLAST scores greater than 500 in both the Thermoprotei and Halobacteria. The KEGG database three letter genome code is given before the colon and can be found here http://www.genome.jp/kegg/catalog/org_list.html. The corresponding gene locus tags are provided after the colon. [file 1471-2148-11-199-S1.GZ › Trees/Htree30.pdf]

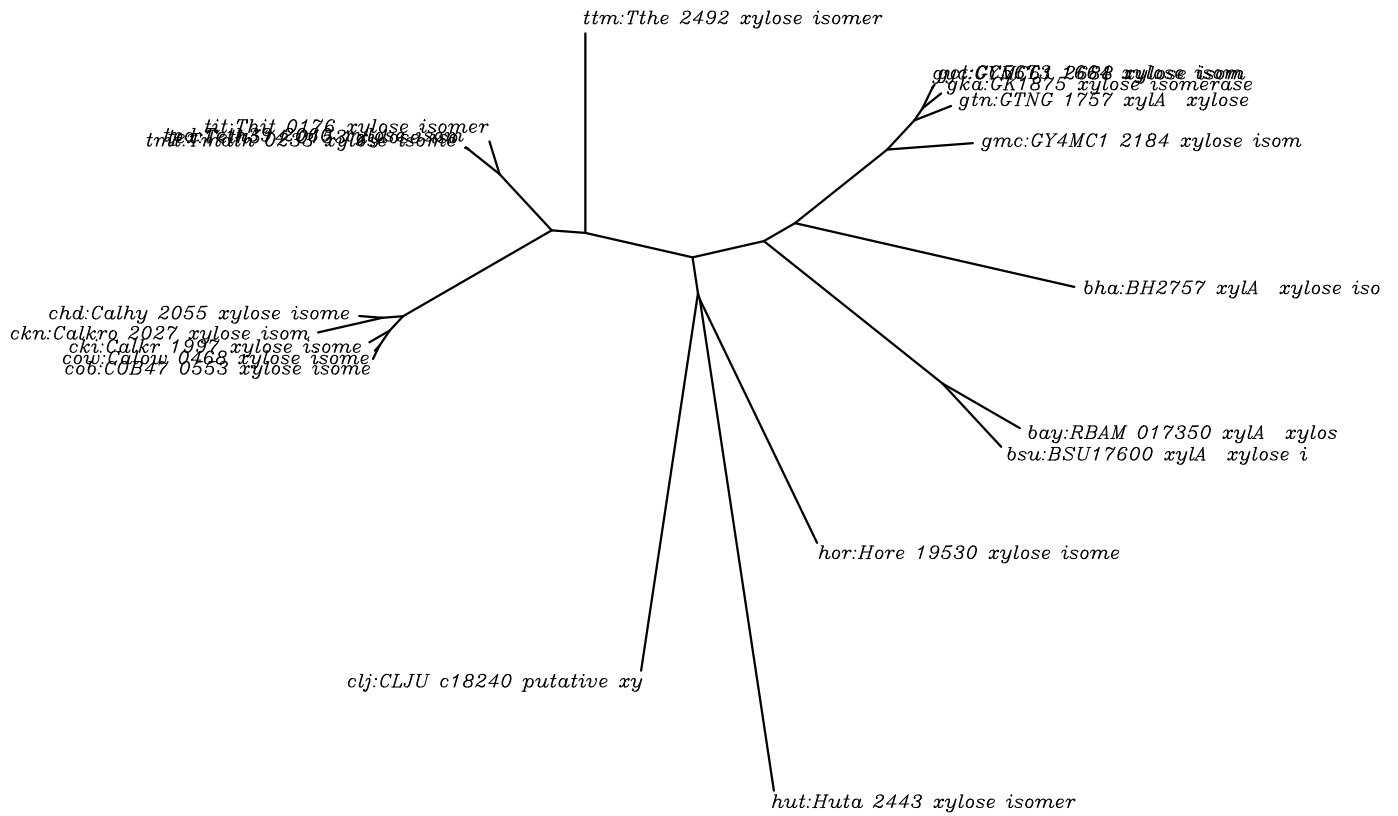

Supplement: Additional file 1 — Collection of phylogenetic trees for Thermoprotei and Halobacteria LGT genes with strong matches. Trees for all LGT genes with BLAST scores greater than 500 in both the Thermoprotei and Halobacteria. The KEGG database three letter genome code is given before the colon and can be found here http://www.genome.jp/kegg/catalog/org_list.html. The corresponding gene locus tags are provided after the colon. [file 1471-2148-11-199-S1.GZ › Trees/Htree31.pdf]

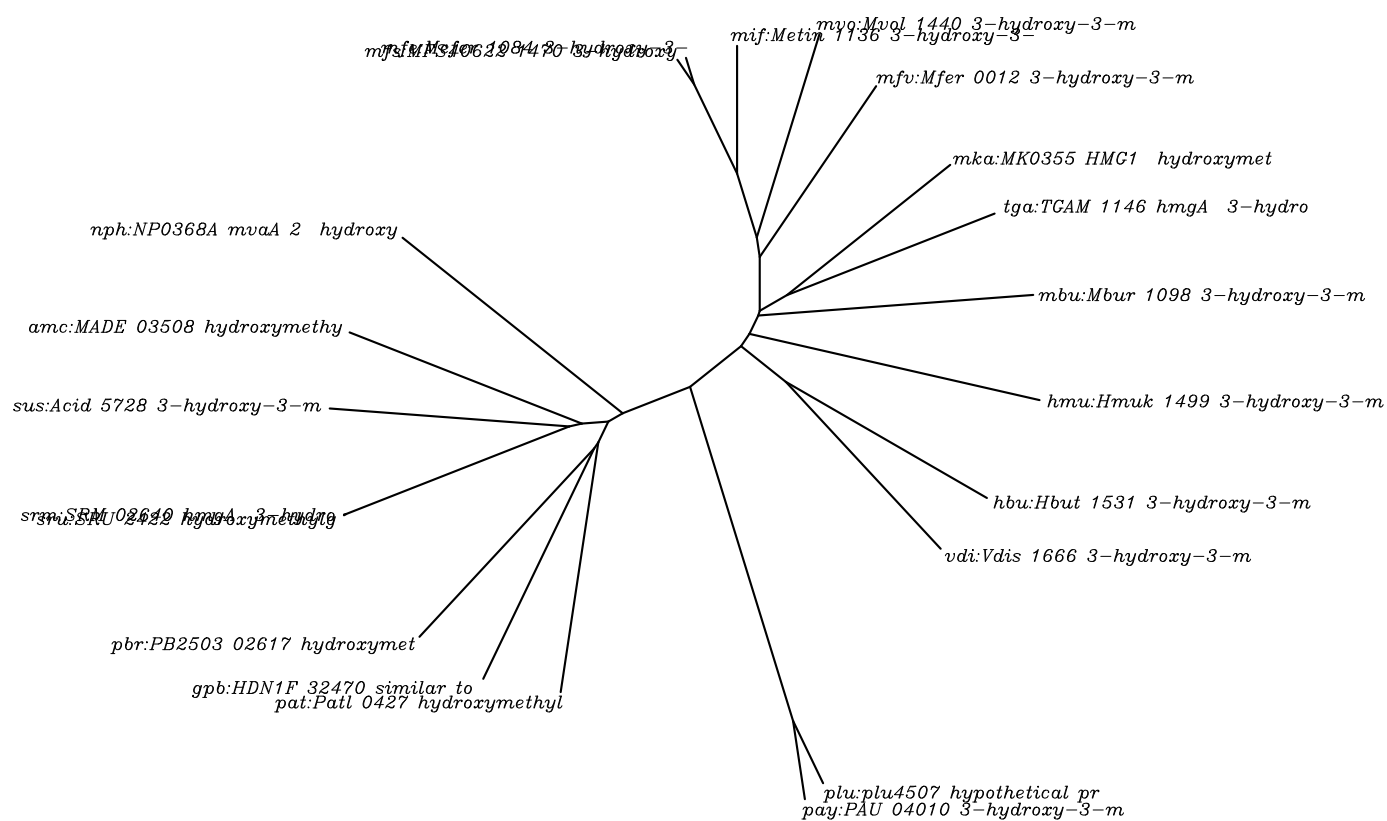

Supplement: Additional file 1 — Collection of phylogenetic trees for Thermoprotei and Halobacteria LGT genes with strong matches. Trees for all LGT genes with BLAST scores greater than 500 in both the Thermoprotei and Halobacteria. The KEGG database three letter genome code is given before the colon and can be found here http://www.genome.jp/kegg/catalog/org_list.html. The corresponding gene locus tags are provided after the colon. [file 1471-2148-11-199-S1.GZ › Trees/Htree32.pdf]

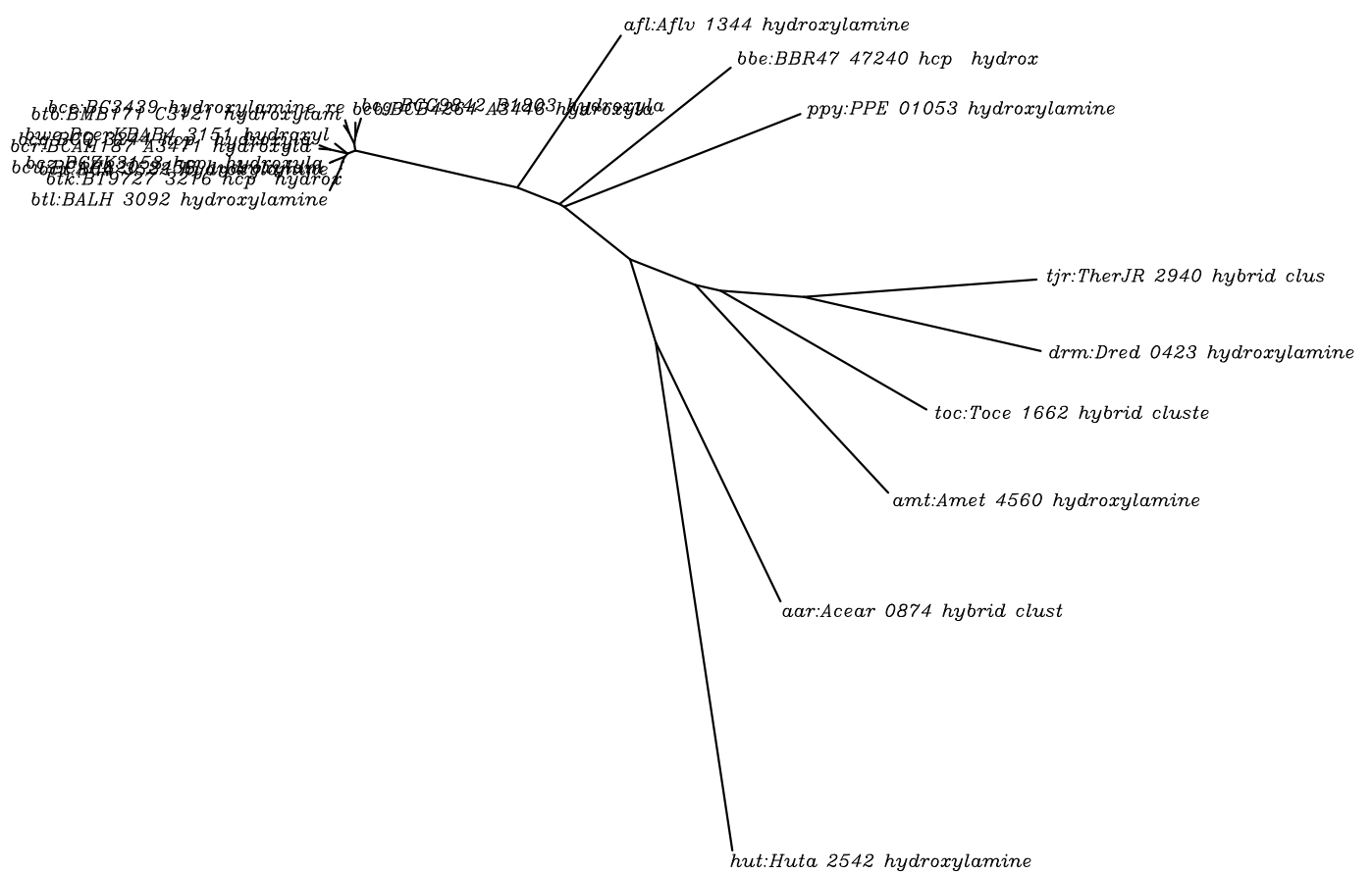

Supplement: Additional file 1 — Collection of phylogenetic trees for Thermoprotei and Halobacteria LGT genes with strong matches. Trees for all LGT genes with BLAST scores greater than 500 in both the Thermoprotei and Halobacteria. The KEGG database three letter genome code is given before the colon and can be found here http://www.genome.jp/kegg/catalog/org_list.html. The corresponding gene locus tags are provided after the colon. [file 1471-2148-11-199-S1.GZ › Trees/Htree33.pdf]

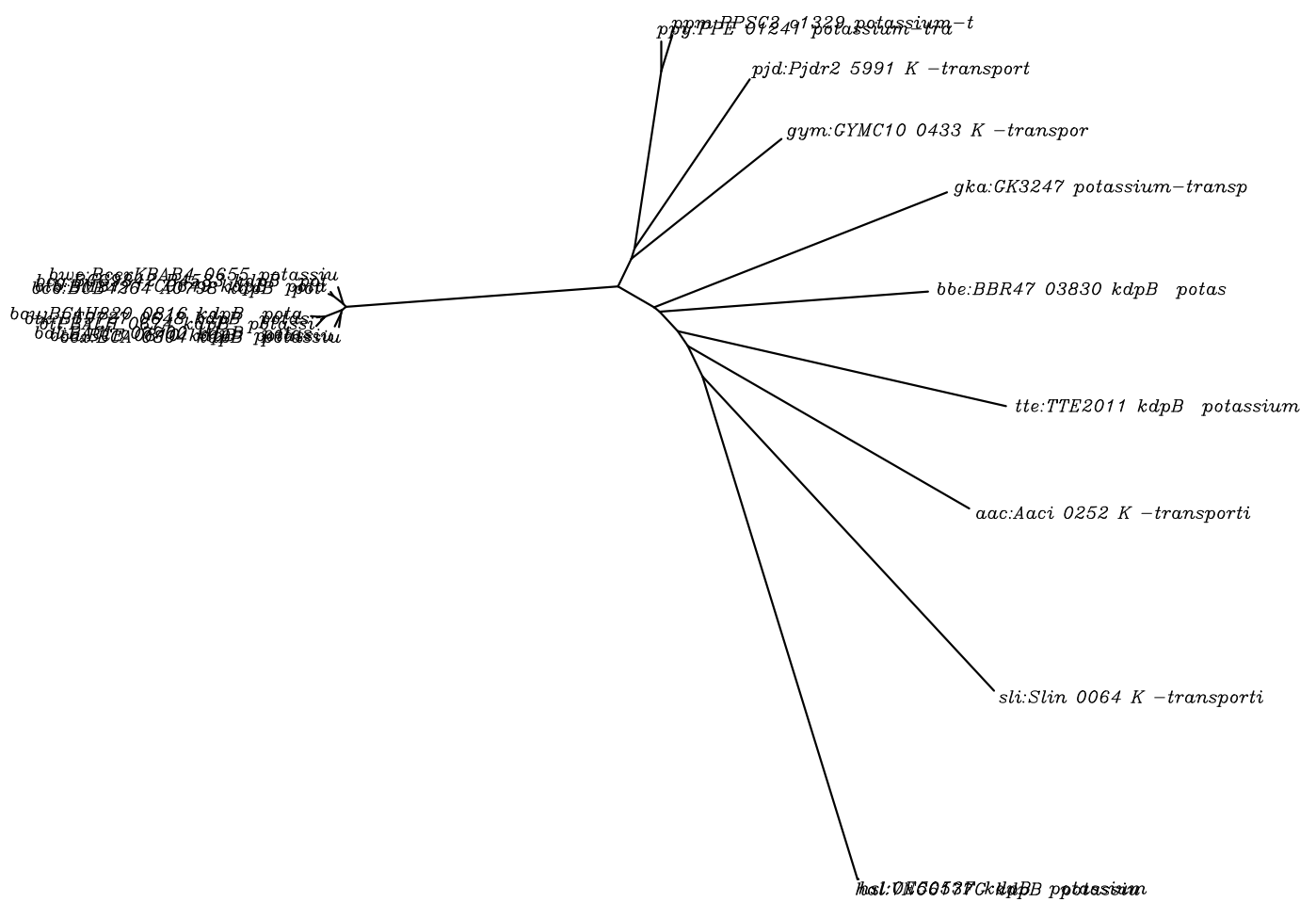

Supplement: Additional file 1 — Collection of phylogenetic trees for Thermoprotei and Halobacteria LGT genes with strong matches. Trees for all LGT genes with BLAST scores greater than 500 in both the Thermoprotei and Halobacteria. The KEGG database three letter genome code is given before the colon and can be found here http://www.genome.jp/kegg/catalog/org_list.html. The corresponding gene locus tags are provided after the colon. [file 1471-2148-11-199-S1.GZ › Trees/Htree34.pdf]

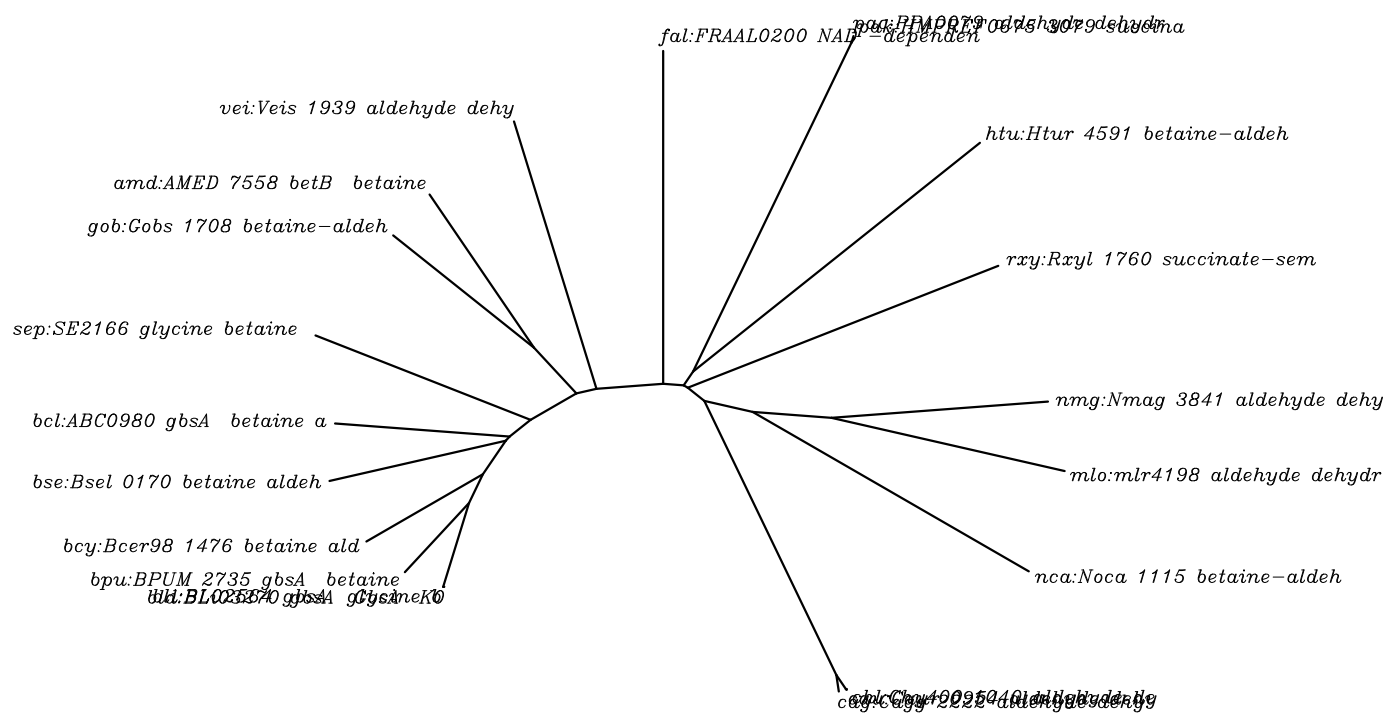

Supplement: Additional file 1 — Collection of phylogenetic trees for Thermoprotei and Halobacteria LGT genes with strong matches. Trees for all LGT genes with BLAST scores greater than 500 in both the Thermoprotei and Halobacteria. The KEGG database three letter genome code is given before the colon and can be found here http://www.genome.jp/kegg/catalog/org_list.html. The corresponding gene locus tags are provided after the colon. [file 1471-2148-11-199-S1.GZ › Trees/Htree35.pdf]

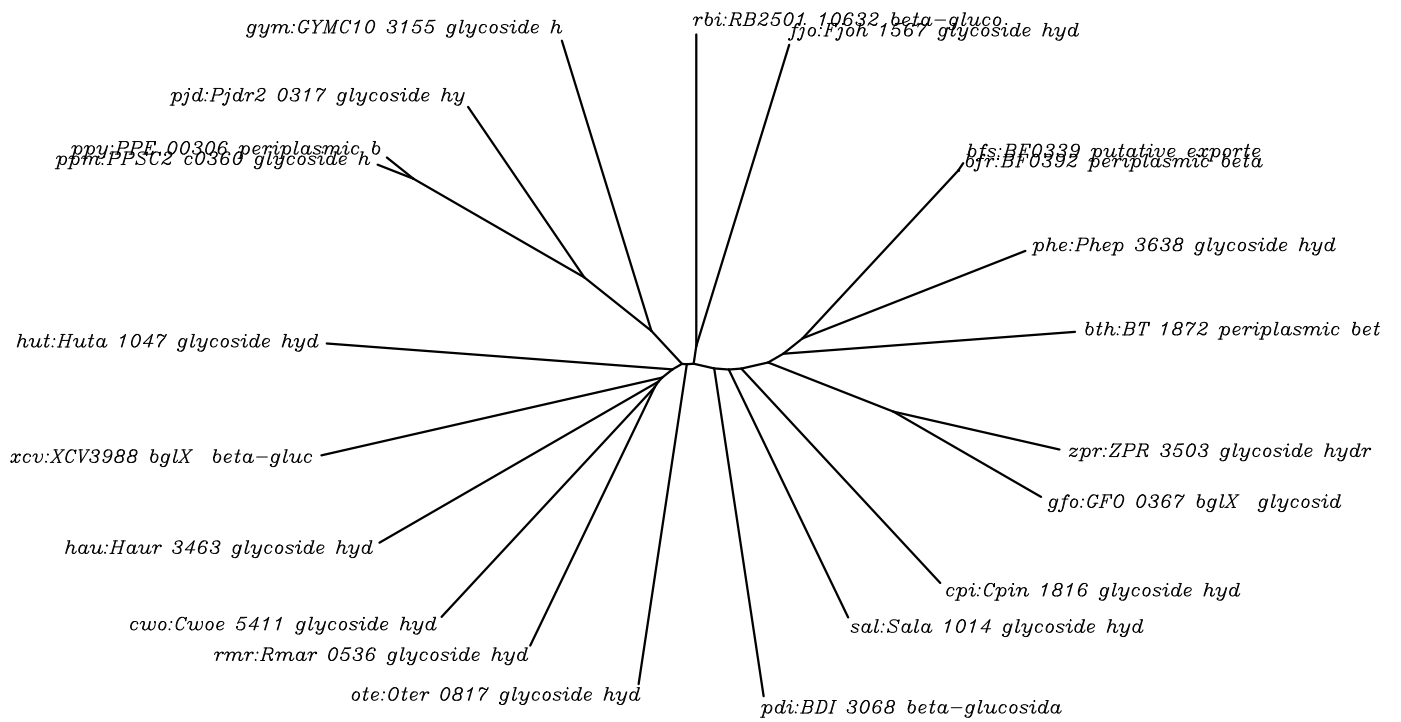

Supplement: Additional file 1 — Collection of phylogenetic trees for Thermoprotei and Halobacteria LGT genes with strong matches. Trees for all LGT genes with BLAST scores greater than 500 in both the Thermoprotei and Halobacteria. The KEGG database three letter genome code is given before the colon and can be found here http://www.genome.jp/kegg/catalog/org_list.html. The corresponding gene locus tags are provided after the colon. [file 1471-2148-11-199-S1.GZ › Trees/Htree36.pdf]

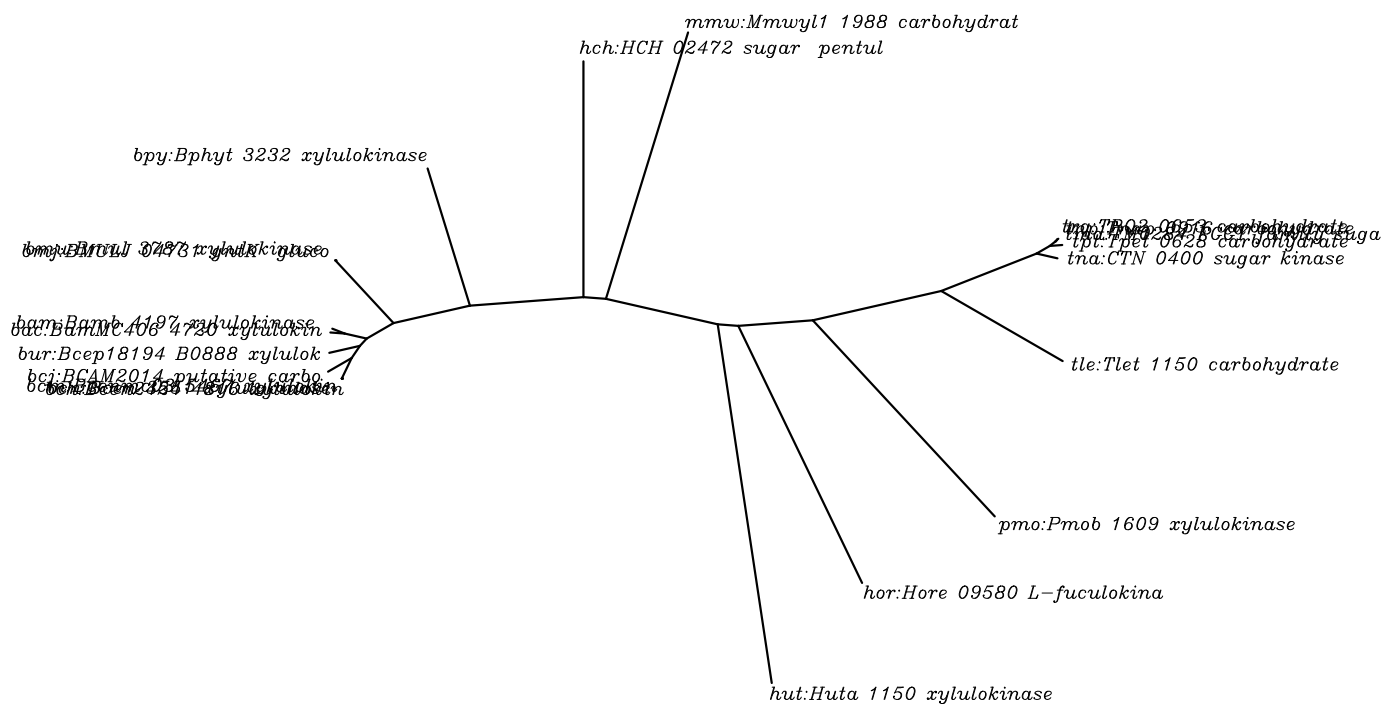

Supplement: Additional file 1 — Collection of phylogenetic trees for Thermoprotei and Halobacteria LGT genes with strong matches. Trees for all LGT genes with BLAST scores greater than 500 in both the Thermoprotei and Halobacteria. The KEGG database three letter genome code is given before the colon and can be found here http://www.genome.jp/kegg/catalog/org_list.html. The corresponding gene locus tags are provided after the colon. [file 1471-2148-11-199-S1.GZ › Trees/Htree37.pdf]

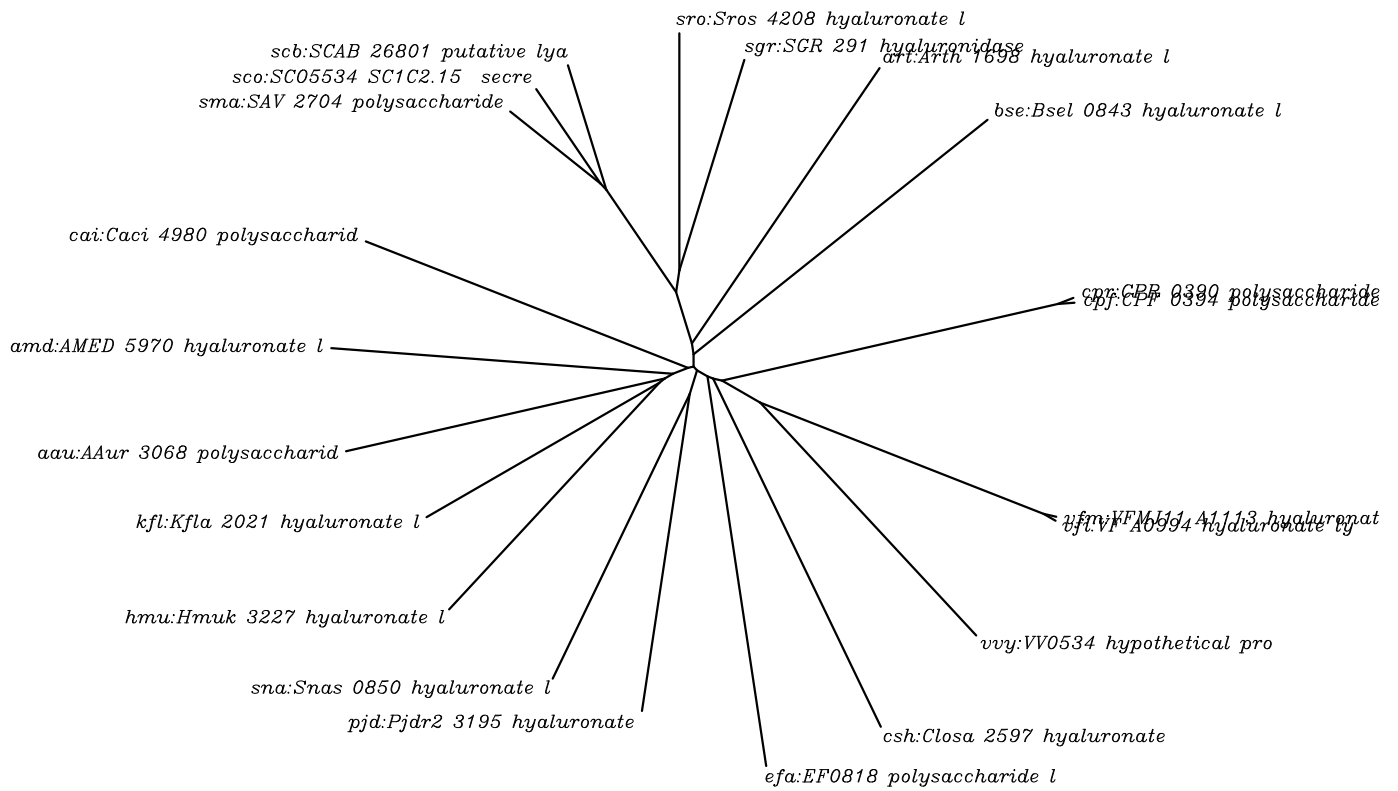

Supplement: Additional file 1 — Collection of phylogenetic trees for Thermoprotei and Halobacteria LGT genes with strong matches. Trees for all LGT genes with BLAST scores greater than 500 in both the Thermoprotei and Halobacteria. The KEGG database three letter genome code is given before the colon and can be found here http://www.genome.jp/kegg/catalog/org_list.html. The corresponding gene locus tags are provided after the colon. [file 1471-2148-11-199-S1.GZ › Trees/Htree38.pdf]

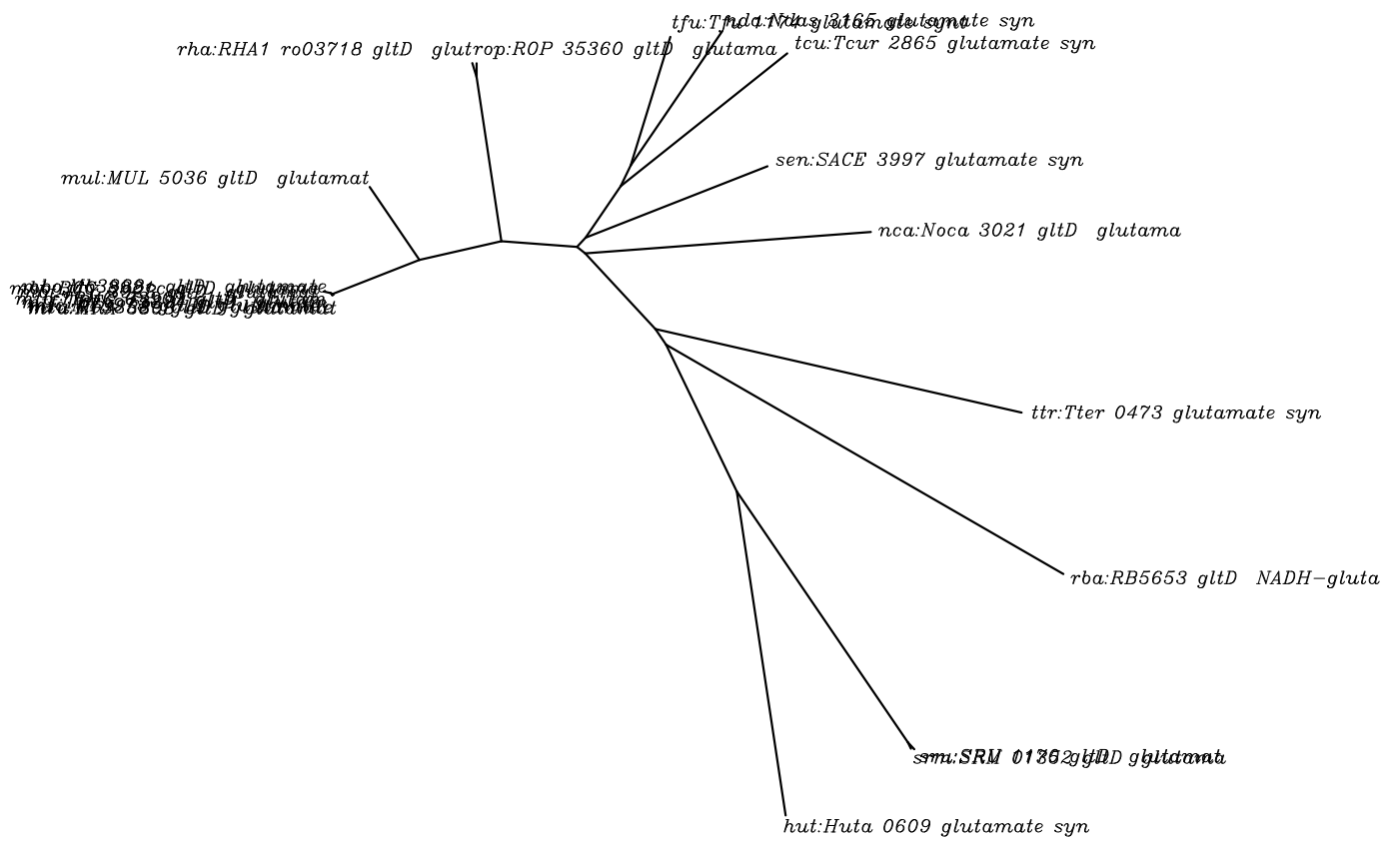

Supplement: Additional file 1 — Collection of phylogenetic trees for Thermoprotei and Halobacteria LGT genes with strong matches. Trees for all LGT genes with BLAST scores greater than 500 in both the Thermoprotei and Halobacteria. The KEGG database three letter genome code is given before the colon and can be found here http://www.genome.jp/kegg/catalog/org_list.html. The corresponding gene locus tags are provided after the colon. [file 1471-2148-11-199-S1.GZ › Trees/Htree39.pdf]

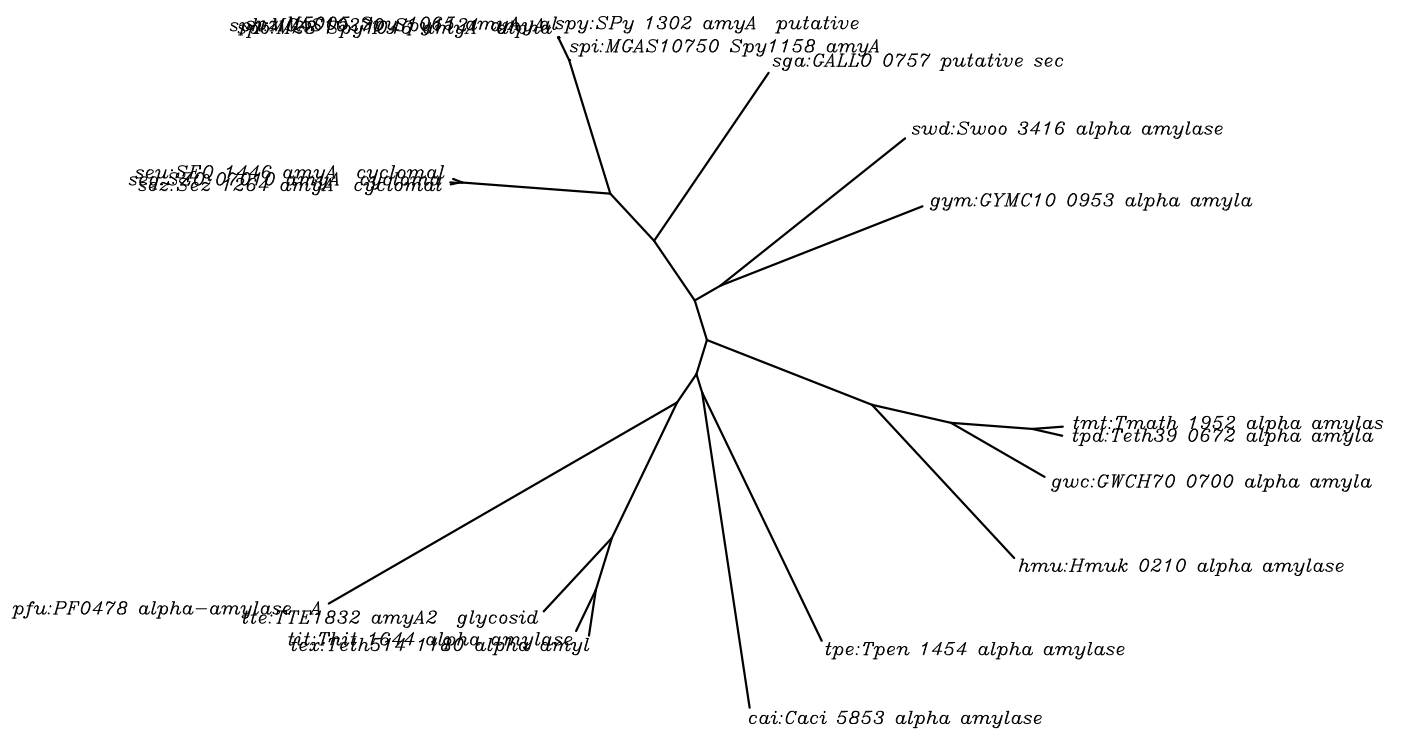

Supplement: Additional file 1 — Collection of phylogenetic trees for Thermoprotei and Halobacteria LGT genes with strong matches. Trees for all LGT genes with BLAST scores greater than 500 in both the Thermoprotei and Halobacteria. The KEGG database three letter genome code is given before the colon and can be found here http://www.genome.jp/kegg/catalog/org_list.html. The corresponding gene locus tags are provided after the colon. [file 1471-2148-11-199-S1.GZ › Trees/Htree4.pdf]

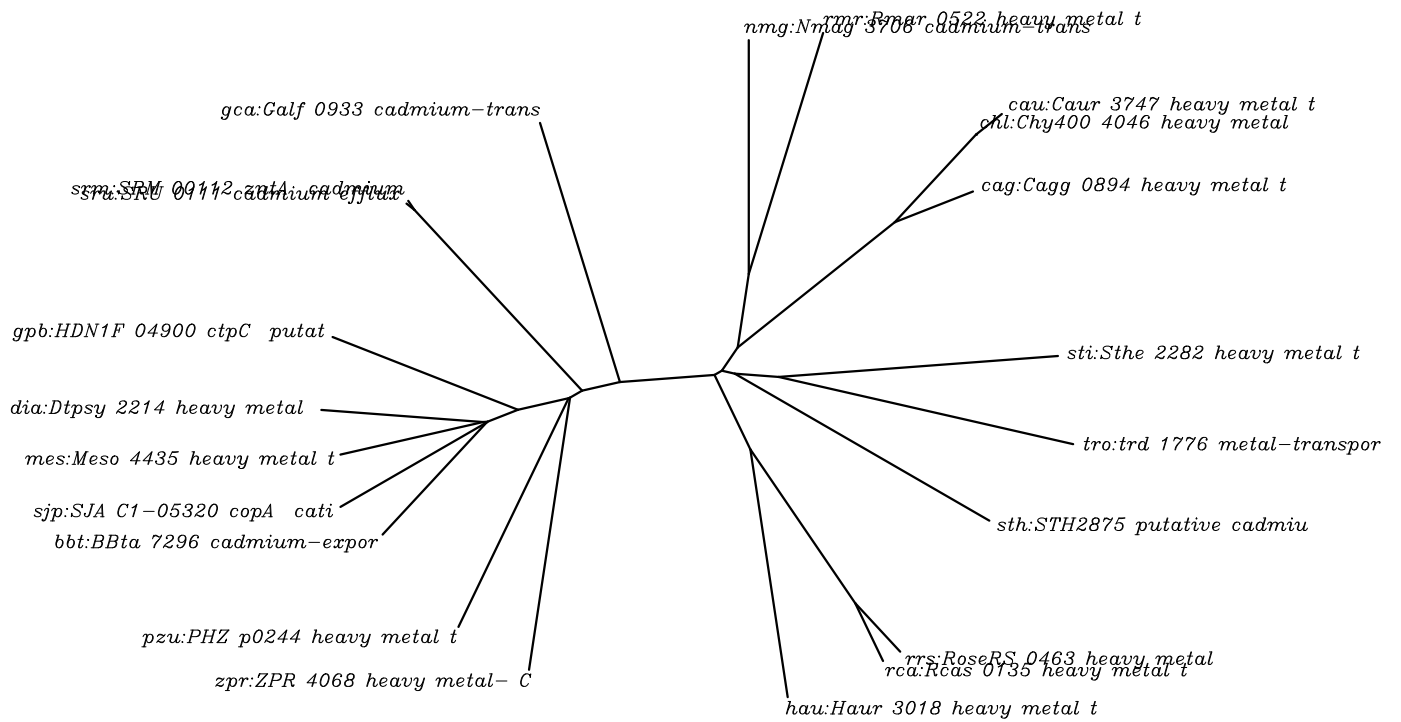

Supplement: Additional file 1 — Collection of phylogenetic trees for Thermoprotei and Halobacteria LGT genes with strong matches. Trees for all LGT genes with BLAST scores greater than 500 in both the Thermoprotei and Halobacteria. The KEGG database three letter genome code is given before the colon and can be found here http://www.genome.jp/kegg/catalog/org_list.html. The corresponding gene locus tags are provided after the colon. [file 1471-2148-11-199-S1.GZ › Trees/Htree40.pdf]

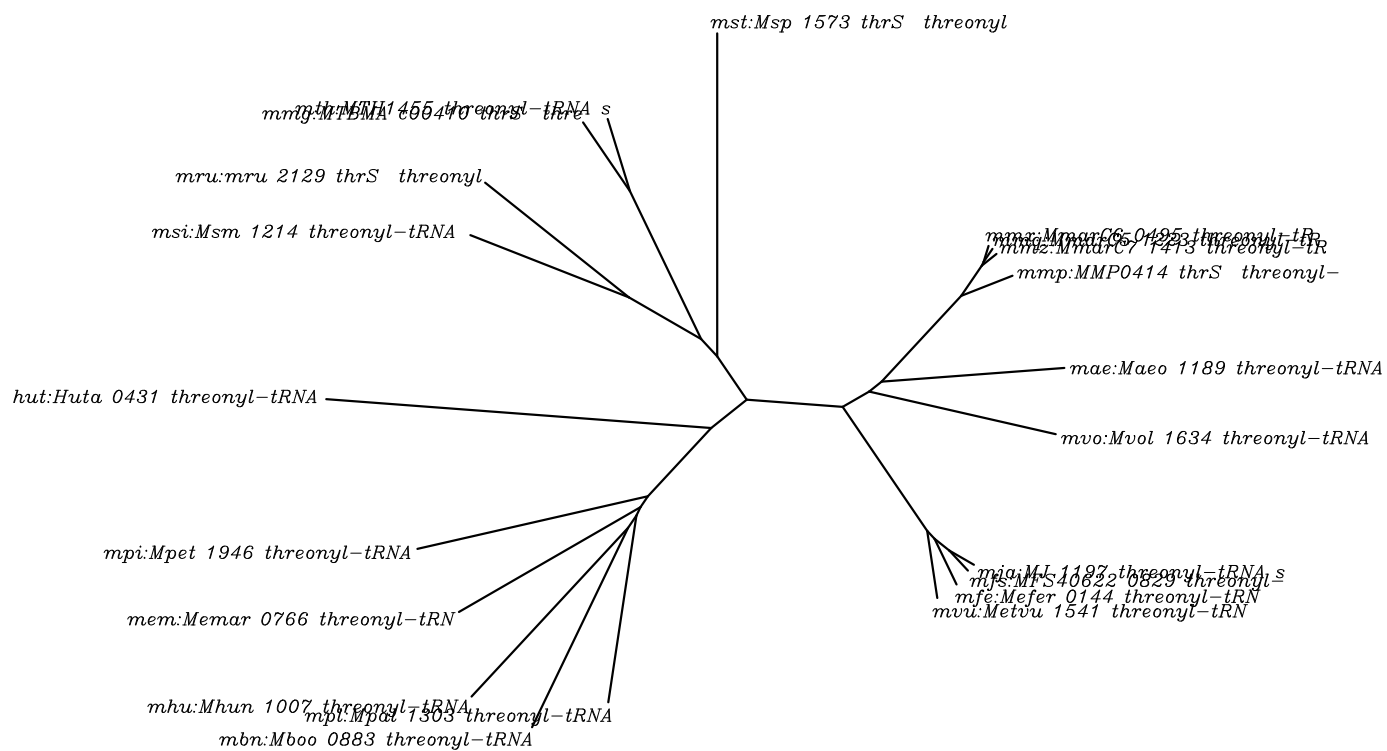

Supplement: Additional file 1 — Collection of phylogenetic trees for Thermoprotei and Halobacteria LGT genes with strong matches. Trees for all LGT genes with BLAST scores greater than 500 in both the Thermoprotei and Halobacteria. The KEGG database three letter genome code is given before the colon and can be found here http://www.genome.jp/kegg/catalog/org_list.html. The corresponding gene locus tags are provided after the colon. [file 1471-2148-11-199-S1.GZ › Trees/Htree41.pdf]

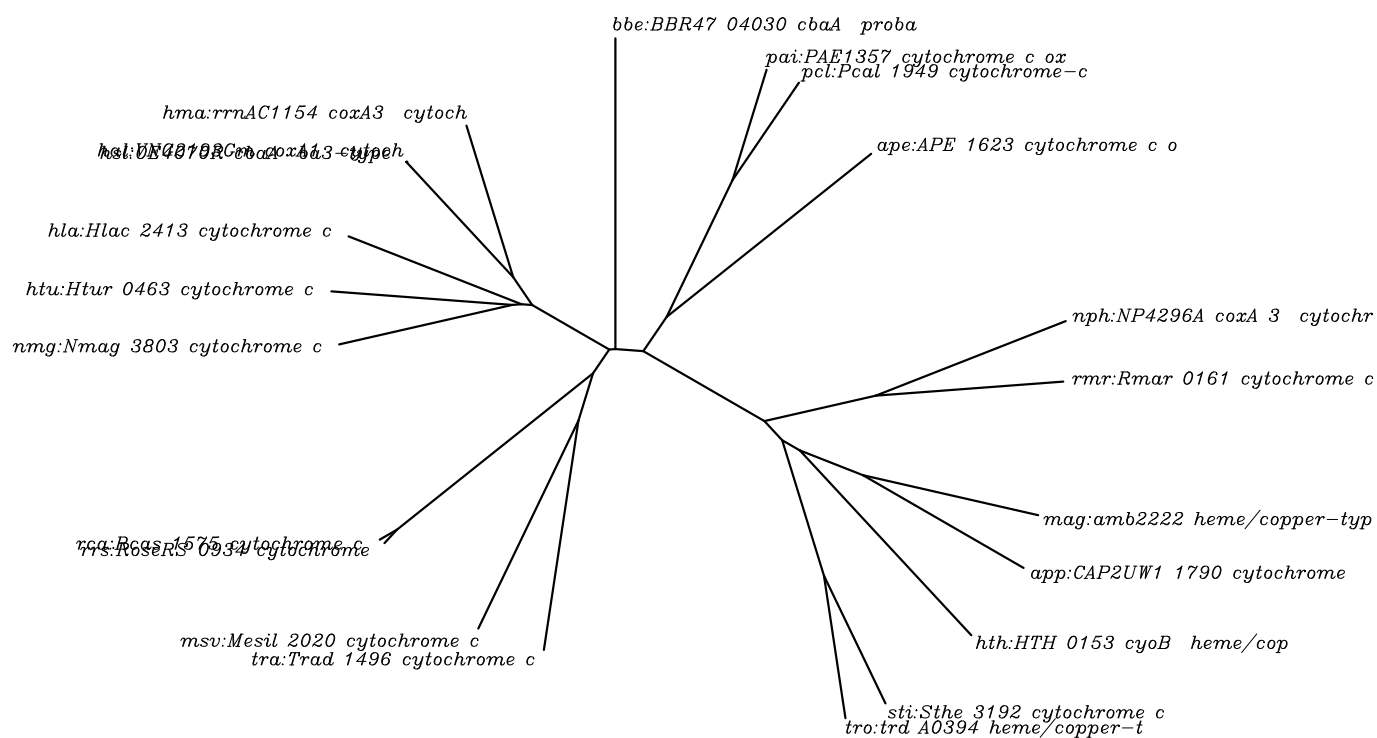

Supplement: Additional file 1 — Collection of phylogenetic trees for Thermoprotei and Halobacteria LGT genes with strong matches. Trees for all LGT genes with BLAST scores greater than 500 in both the Thermoprotei and Halobacteria. The KEGG database three letter genome code is given before the colon and can be found here http://www.genome.jp/kegg/catalog/org_list.html. The corresponding gene locus tags are provided after the colon. [file 1471-2148-11-199-S1.GZ › Trees/Htree42.pdf]

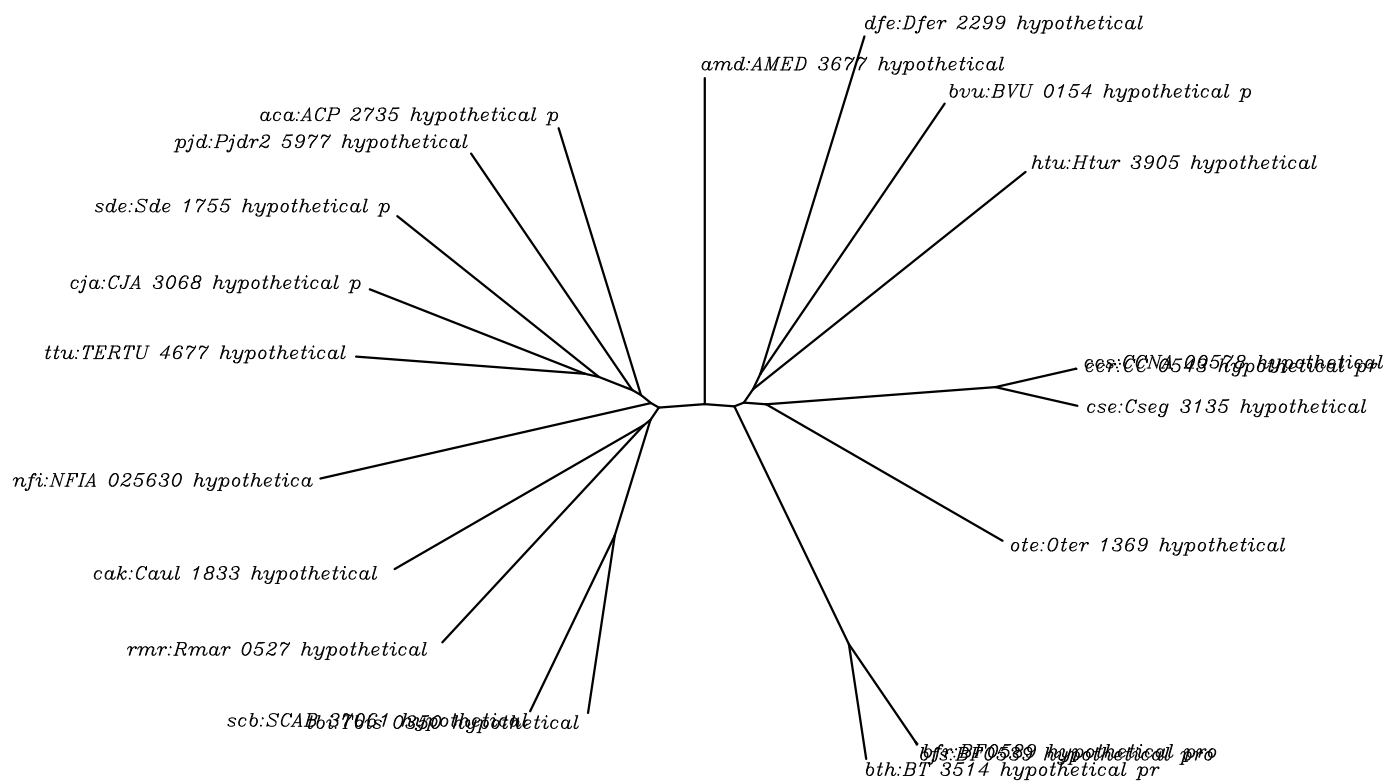

Supplement: Additional file 1 — Collection of phylogenetic trees for Thermoprotei and Halobacteria LGT genes with strong matches. Trees for all LGT genes with BLAST scores greater than 500 in both the Thermoprotei and Halobacteria. The KEGG database three letter genome code is given before the colon and can be found here http://www.genome.jp/kegg/catalog/org_list.html. The corresponding gene locus tags are provided after the colon. [file 1471-2148-11-199-S1.GZ › Trees/Htree43.pdf]

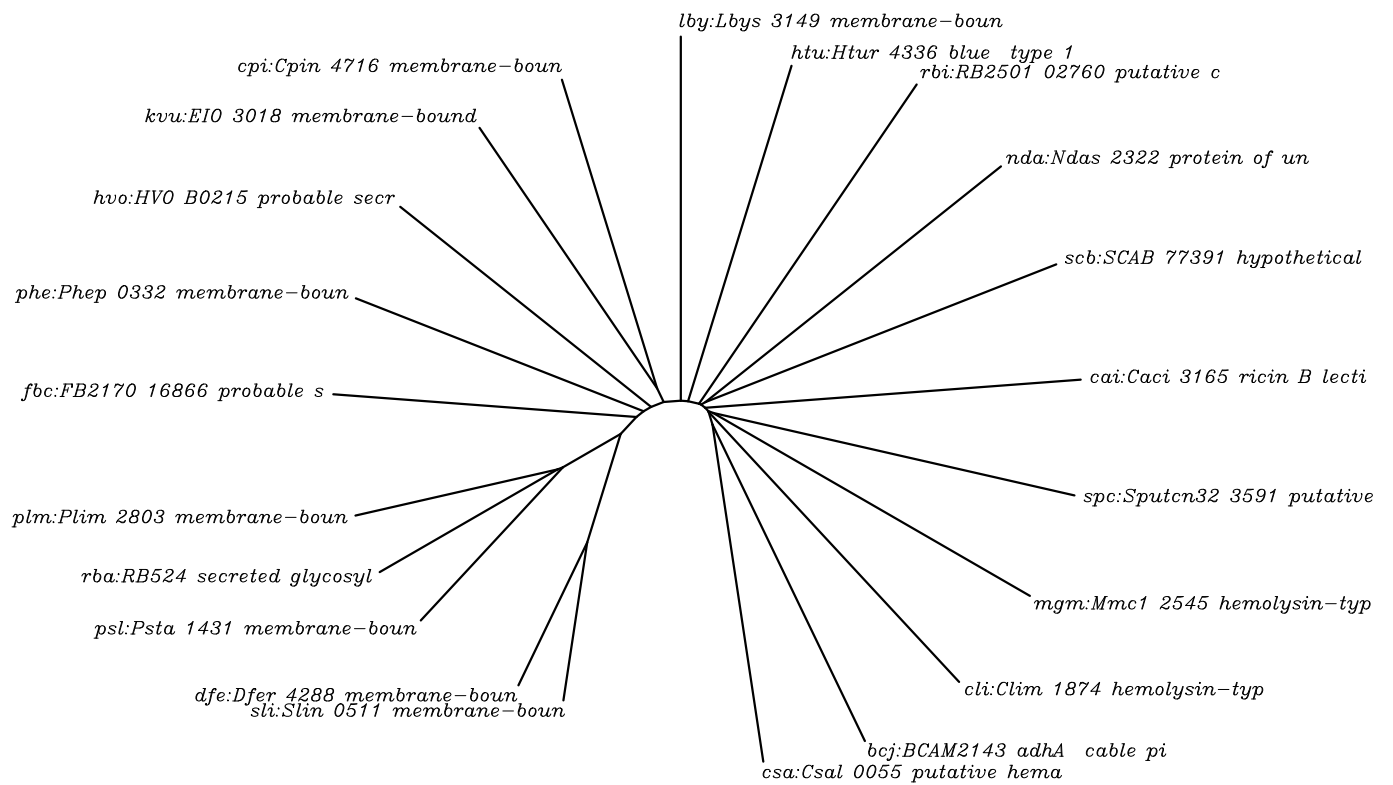

Supplement: Additional file 1 — Collection of phylogenetic trees for Thermoprotei and Halobacteria LGT genes with strong matches. Trees for all LGT genes with BLAST scores greater than 500 in both the Thermoprotei and Halobacteria. The KEGG database three letter genome code is given before the colon and can be found here http://www.genome.jp/kegg/catalog/org_list.html. The corresponding gene locus tags are provided after the colon. [file 1471-2148-11-199-S1.GZ › Trees/Htree44.pdf]

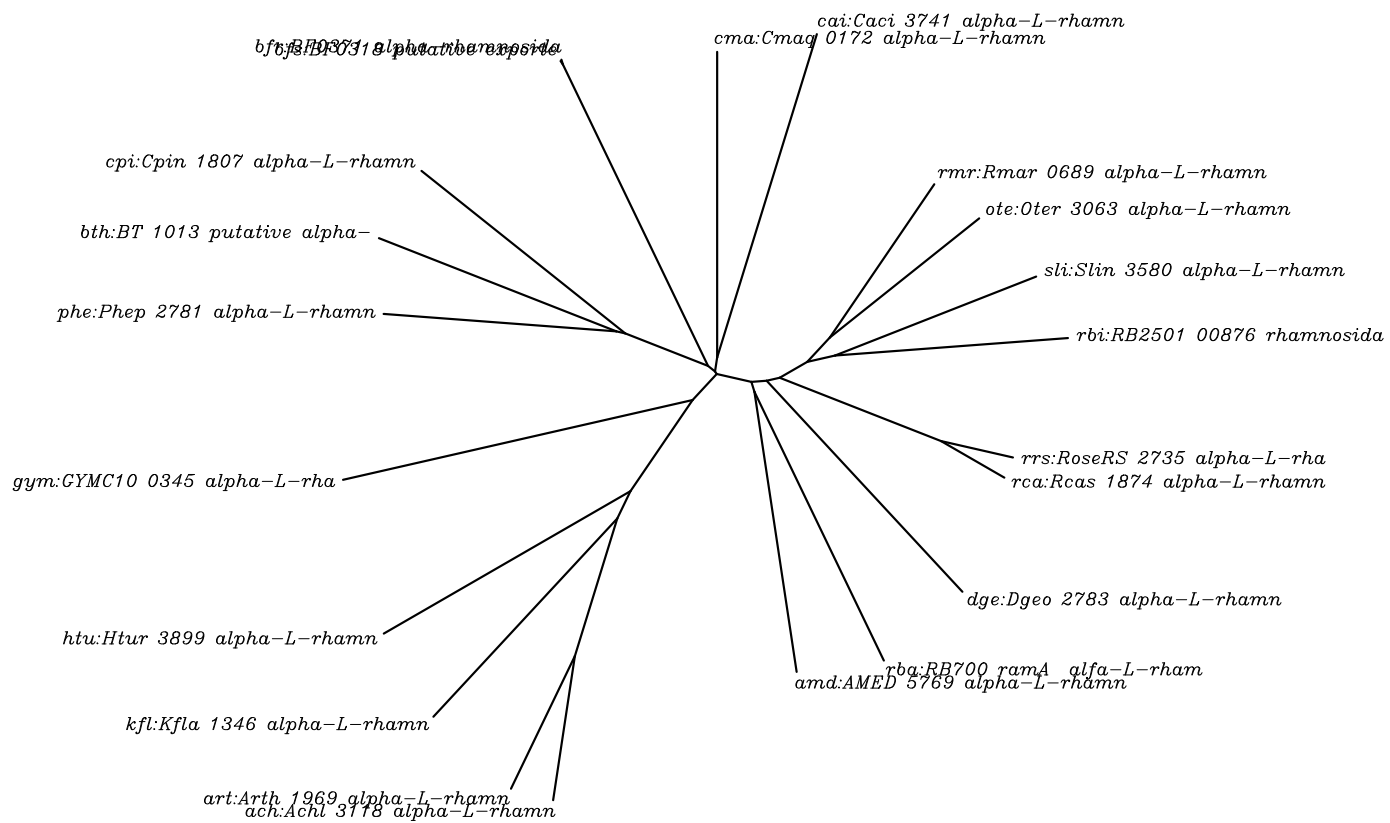

Supplement: Additional file 1 — Collection of phylogenetic trees for Thermoprotei and Halobacteria LGT genes with strong matches. Trees for all LGT genes with BLAST scores greater than 500 in both the Thermoprotei and Halobacteria. The KEGG database three letter genome code is given before the colon and can be found here http://www.genome.jp/kegg/catalog/org_list.html. The corresponding gene locus tags are provided after the colon. [file 1471-2148-11-199-S1.GZ › Trees/Htree45.pdf]

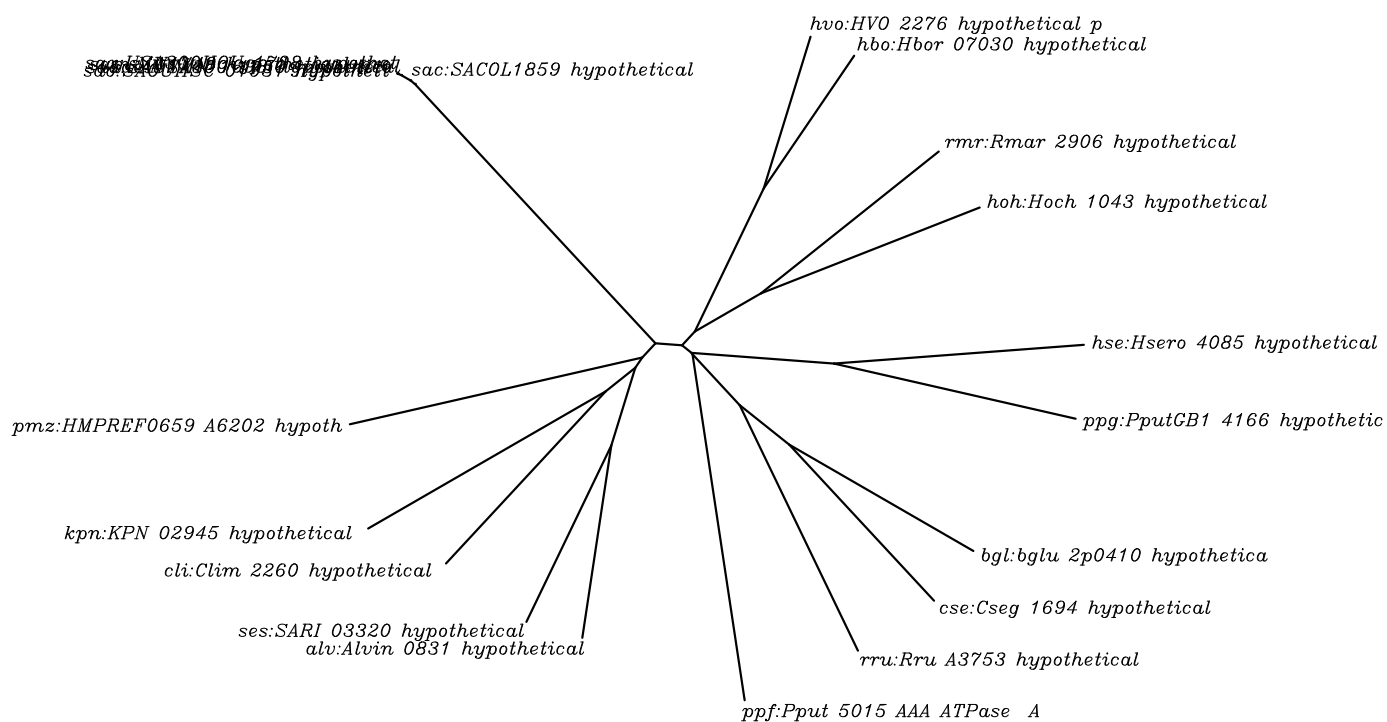

Supplement: Additional file 1 — Collection of phylogenetic trees for Thermoprotei and Halobacteria LGT genes with strong matches. Trees for all LGT genes with BLAST scores greater than 500 in both the Thermoprotei and Halobacteria. The KEGG database three letter genome code is given before the colon and can be found here http://www.genome.jp/kegg/catalog/org_list.html. The corresponding gene locus tags are provided after the colon. [file 1471-2148-11-199-S1.GZ › Trees/Htree46.pdf]

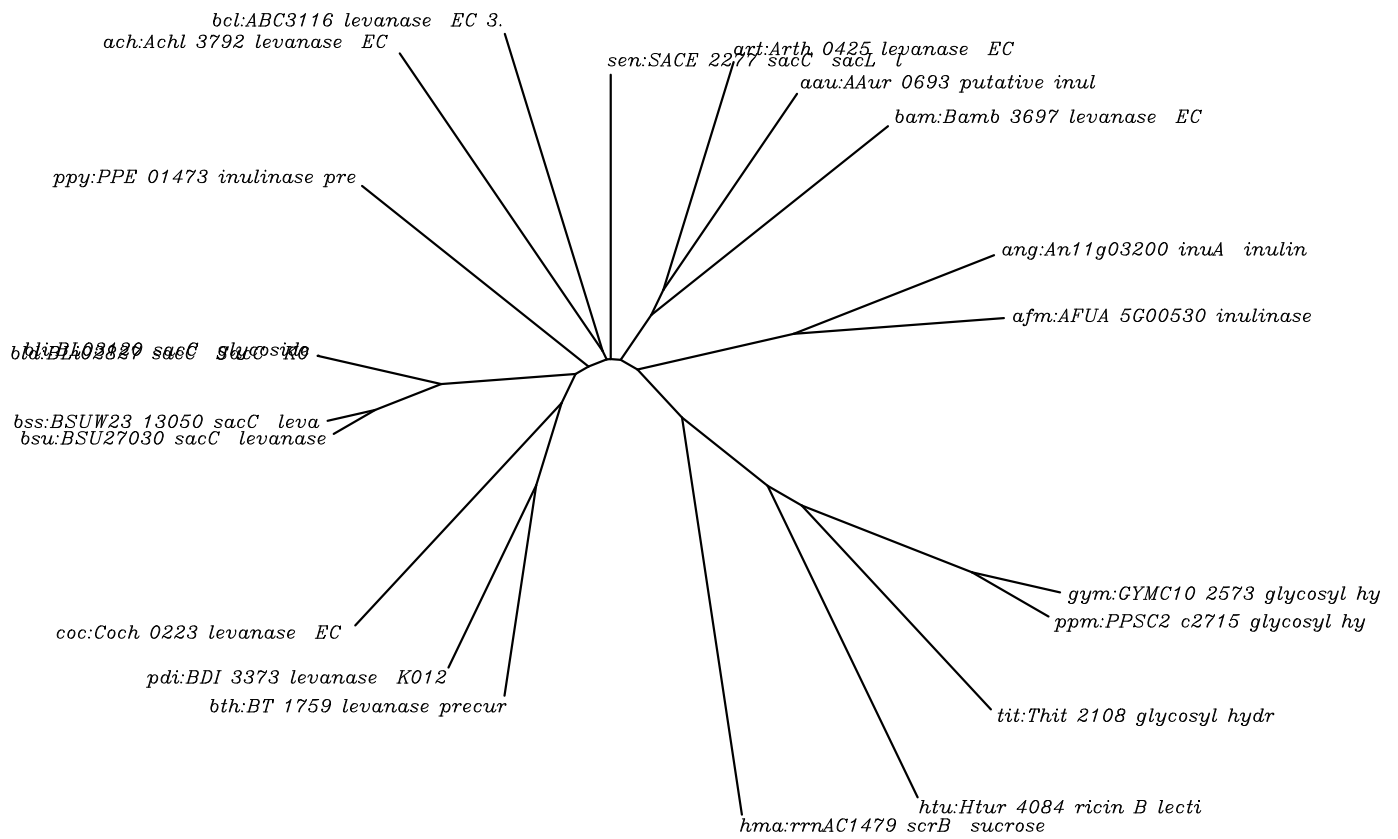

Supplement: Additional file 1 — Collection of phylogenetic trees for Thermoprotei and Halobacteria LGT genes with strong matches. Trees for all LGT genes with BLAST scores greater than 500 in both the Thermoprotei and Halobacteria. The KEGG database three letter genome code is given before the colon and can be found here http://www.genome.jp/kegg/catalog/org_list.html. The corresponding gene locus tags are provided after the colon. [file 1471-2148-11-199-S1.GZ › Trees/Htree47.pdf]

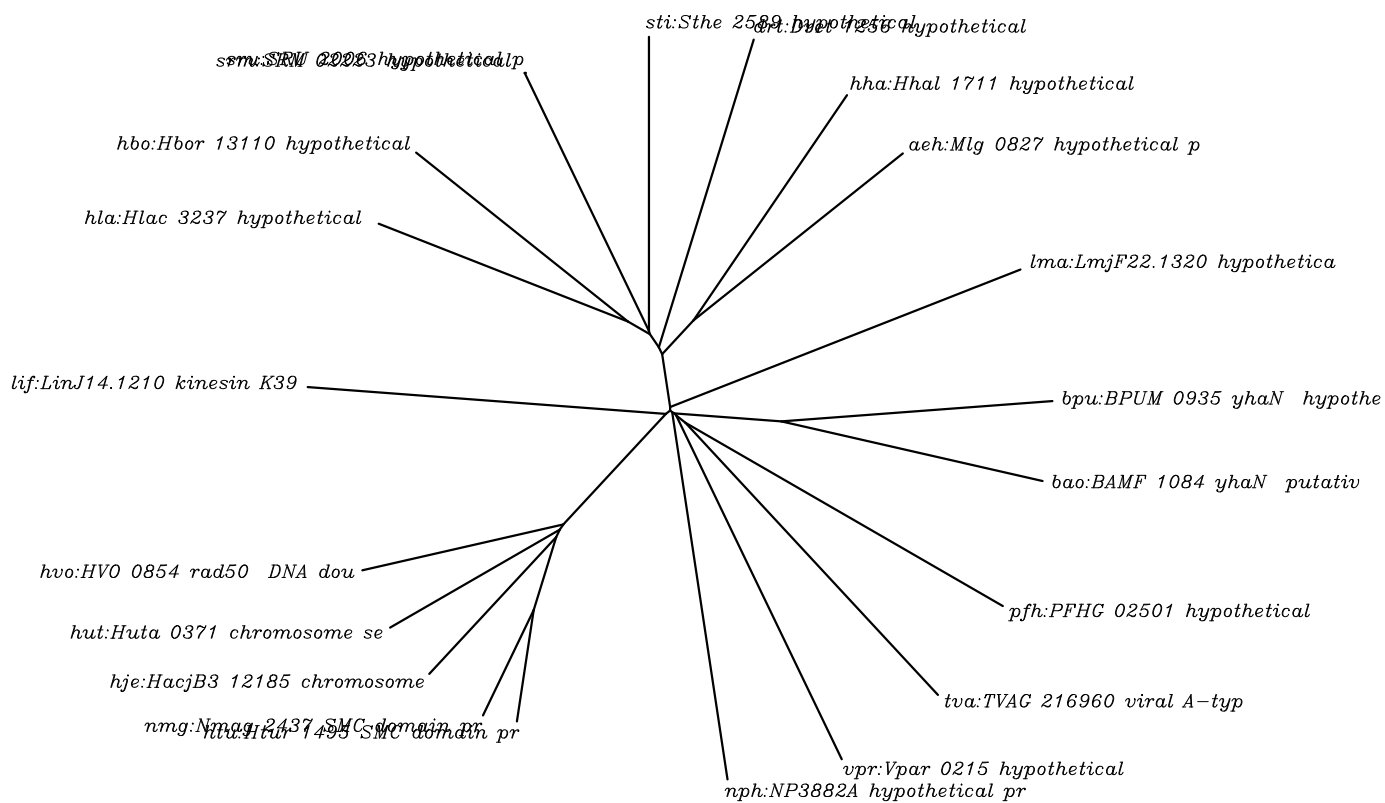

Supplement: Additional file 1 — Collection of phylogenetic trees for Thermoprotei and Halobacteria LGT genes with strong matches. Trees for all LGT genes with BLAST scores greater than 500 in both the Thermoprotei and Halobacteria. The KEGG database three letter genome code is given before the colon and can be found here http://www.genome.jp/kegg/catalog/org_list.html. The corresponding gene locus tags are provided after the colon. [file 1471-2148-11-199-S1.GZ › Trees/Htree48.pdf]

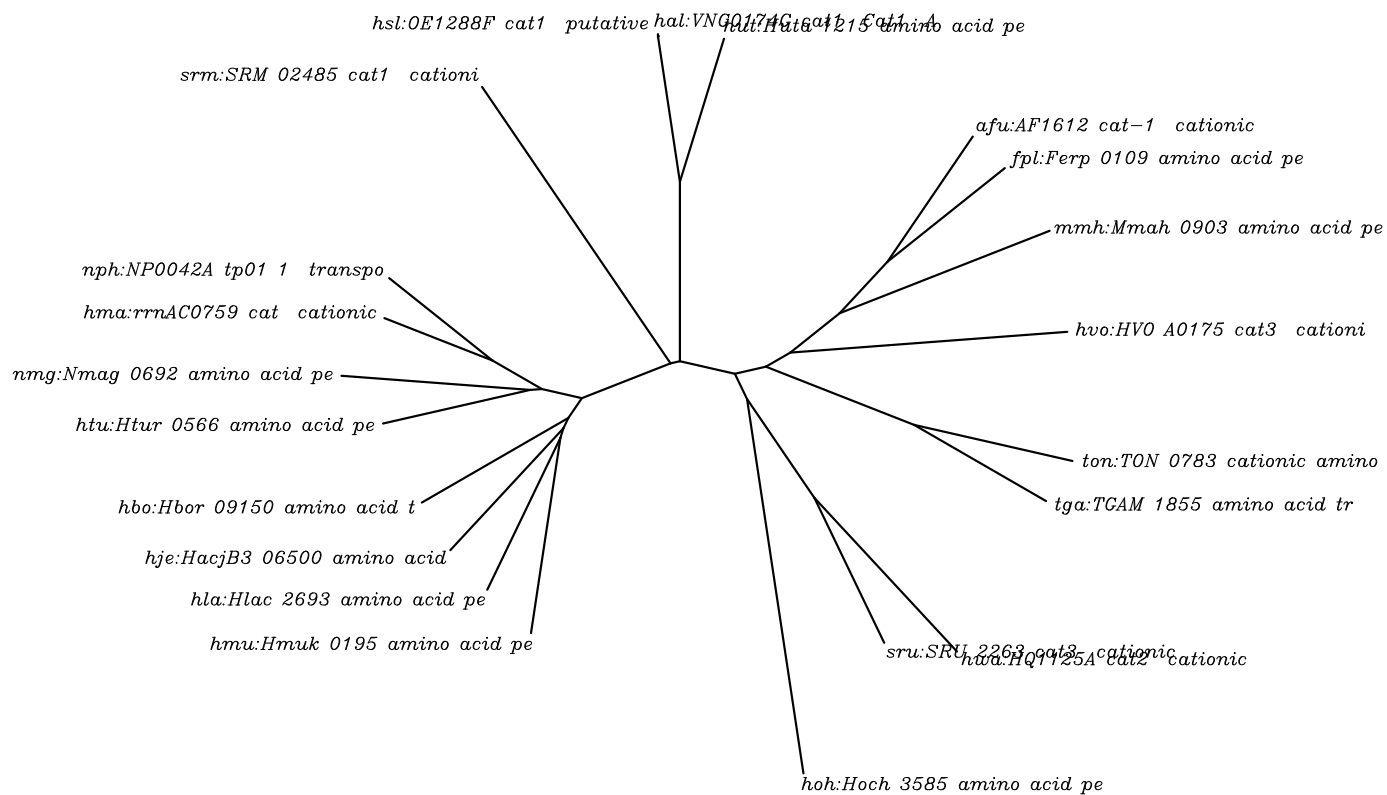

Supplement: Additional file 1 — Collection of phylogenetic trees for Thermoprotei and Halobacteria LGT genes with strong matches. Trees for all LGT genes with BLAST scores greater than 500 in both the Thermoprotei and Halobacteria. The KEGG database three letter genome code is given before the colon and can be found here http://www.genome.jp/kegg/catalog/org_list.html. The corresponding gene locus tags are provided after the colon. [file 1471-2148-11-199-S1.GZ › Trees/Htree49.pdf]

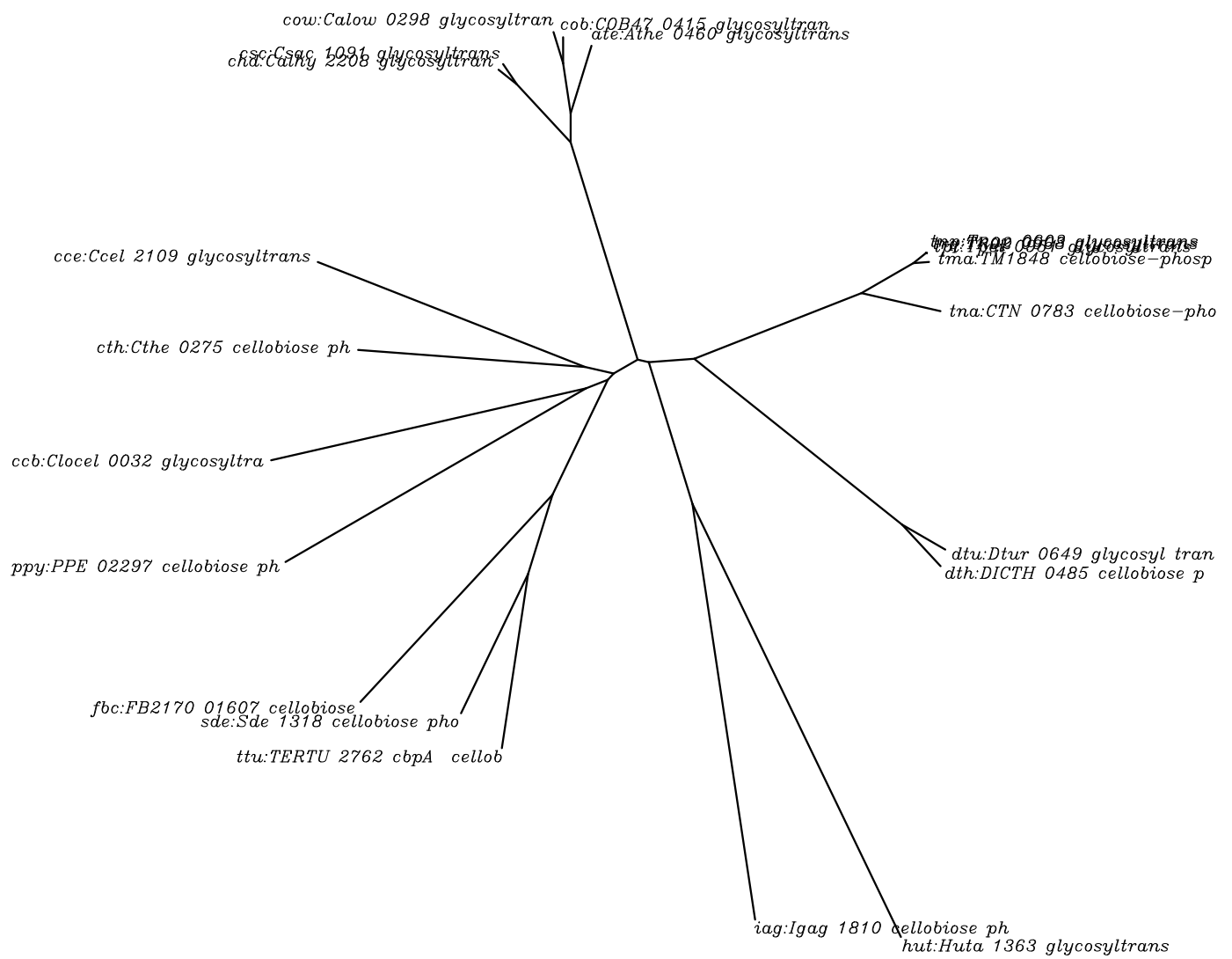

Supplement: Additional file 1 — Collection of phylogenetic trees for Thermoprotei and Halobacteria LGT genes with strong matches. Trees for all LGT genes with BLAST scores greater than 500 in both the Thermoprotei and Halobacteria. The KEGG database three letter genome code is given before the colon and can be found here http://www.genome.jp/kegg/catalog/org_list.html. The corresponding gene locus tags are provided after the colon. [file 1471-2148-11-199-S1.GZ › Trees/Htree5.pdf]

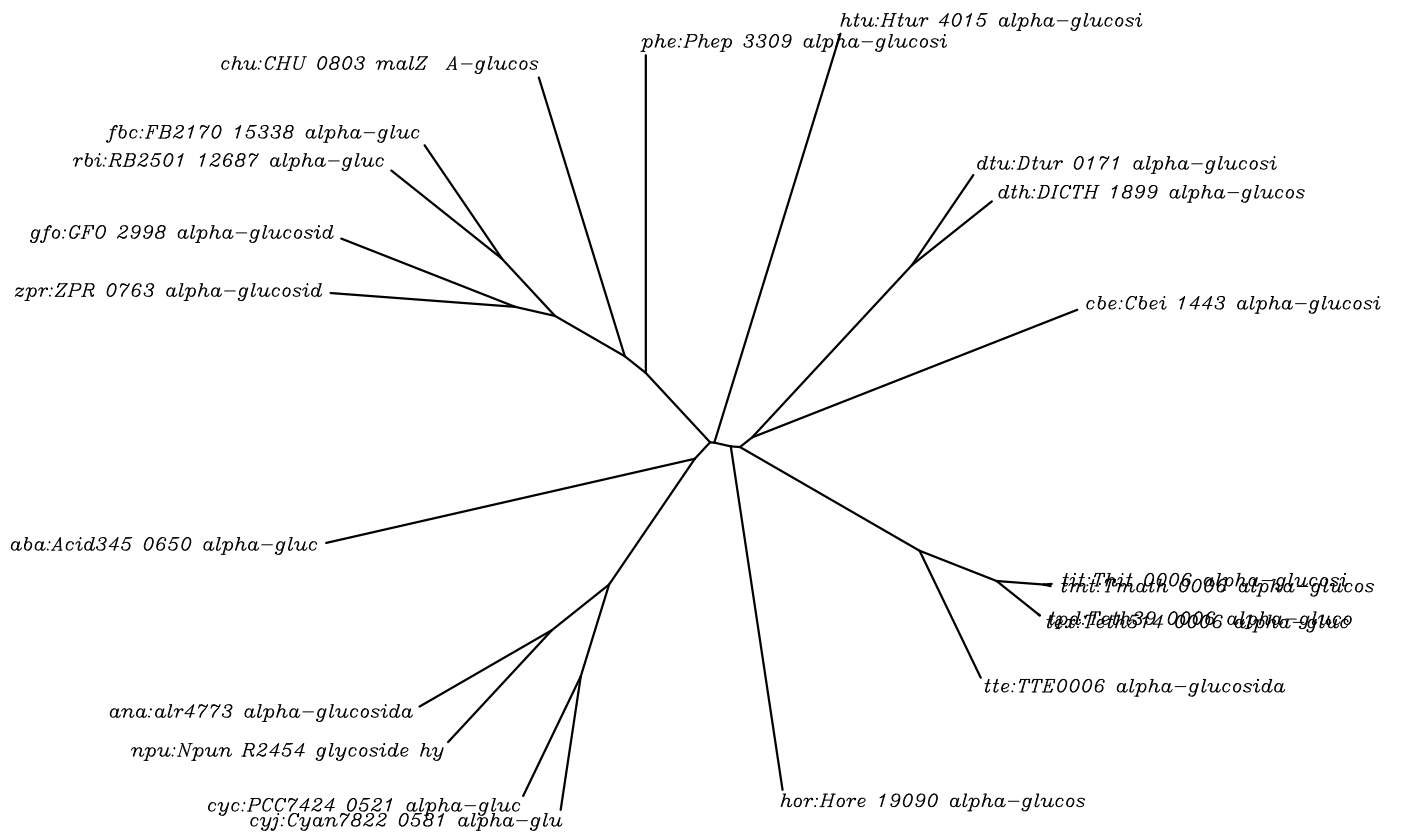

Supplement: Additional file 1 — Collection of phylogenetic trees for Thermoprotei and Halobacteria LGT genes with strong matches. Trees for all LGT genes with BLAST scores greater than 500 in both the Thermoprotei and Halobacteria. The KEGG database three letter genome code is given before the colon and can be found here http://www.genome.jp/kegg/catalog/org_list.html. The corresponding gene locus tags are provided after the colon. [file 1471-2148-11-199-S1.GZ › Trees/Htree50.pdf]

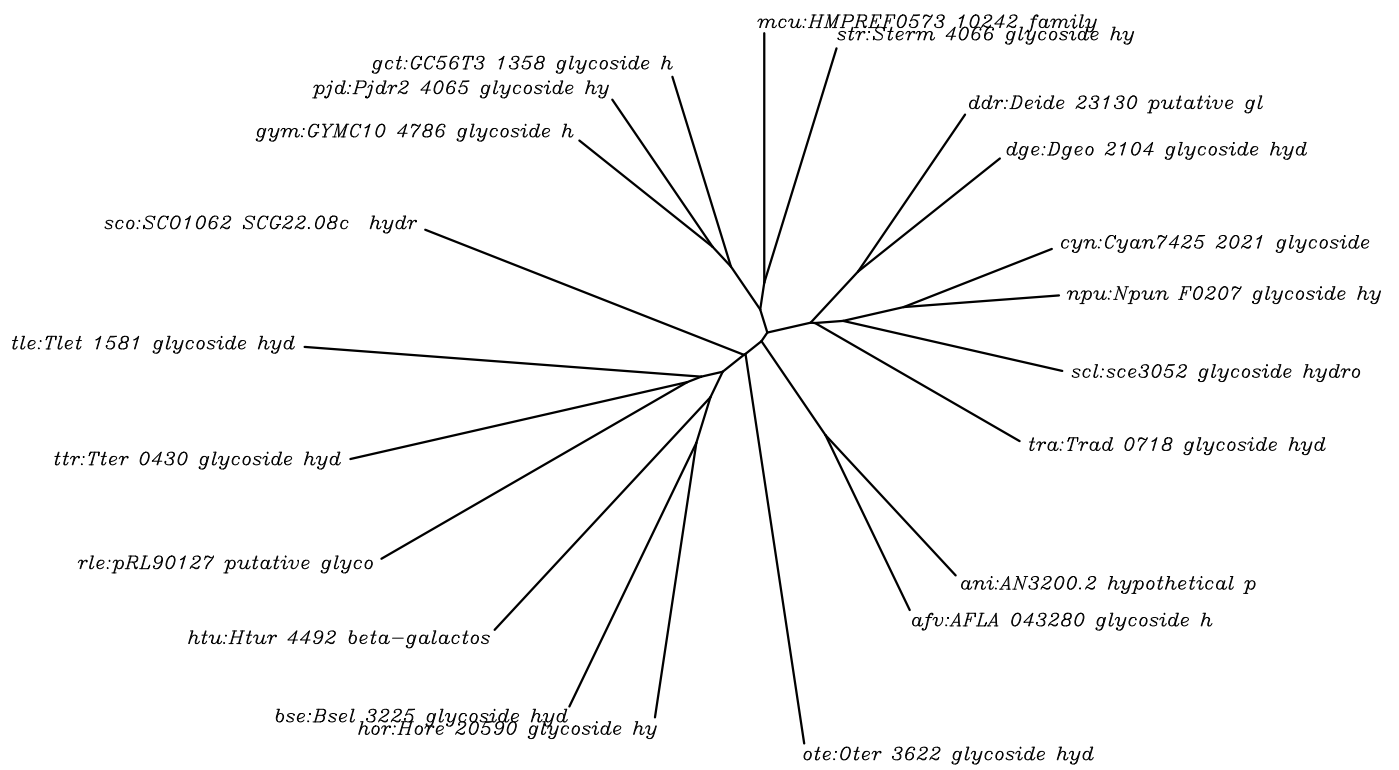

Supplement: Additional file 1 — Collection of phylogenetic trees for Thermoprotei and Halobacteria LGT genes with strong matches. Trees for all LGT genes with BLAST scores greater than 500 in both the Thermoprotei and Halobacteria. The KEGG database three letter genome code is given before the colon and can be found here http://www.genome.jp/kegg/catalog/org_list.html. The corresponding gene locus tags are provided after the colon. [file 1471-2148-11-199-S1.GZ › Trees/Htree51.pdf]

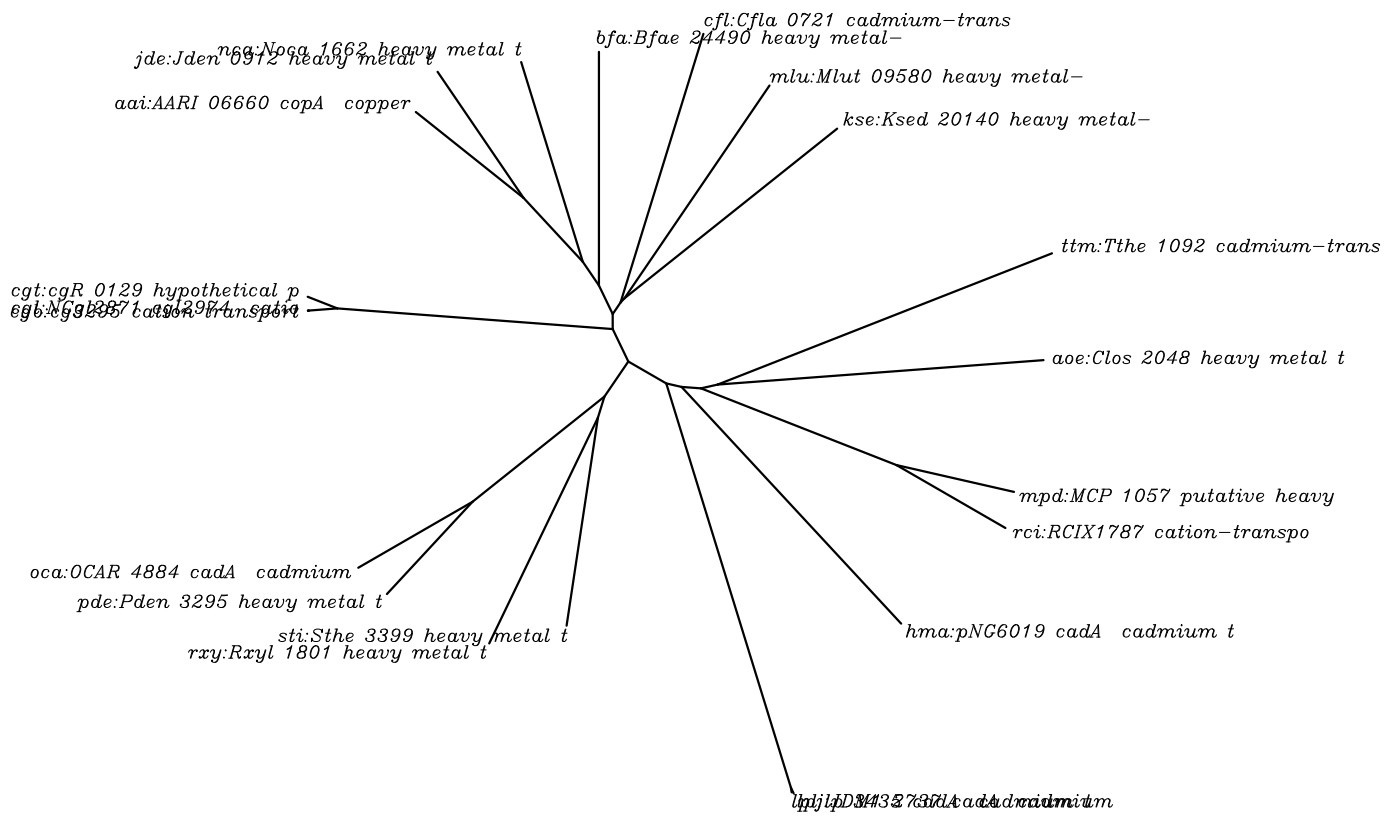

Supplement: Additional file 1 — Collection of phylogenetic trees for Thermoprotei and Halobacteria LGT genes with strong matches. Trees for all LGT genes with BLAST scores greater than 500 in both the Thermoprotei and Halobacteria. The KEGG database three letter genome code is given before the colon and can be found here http://www.genome.jp/kegg/catalog/org_list.html. The corresponding gene locus tags are provided after the colon. [file 1471-2148-11-199-S1.GZ › Trees/Htree52.pdf]

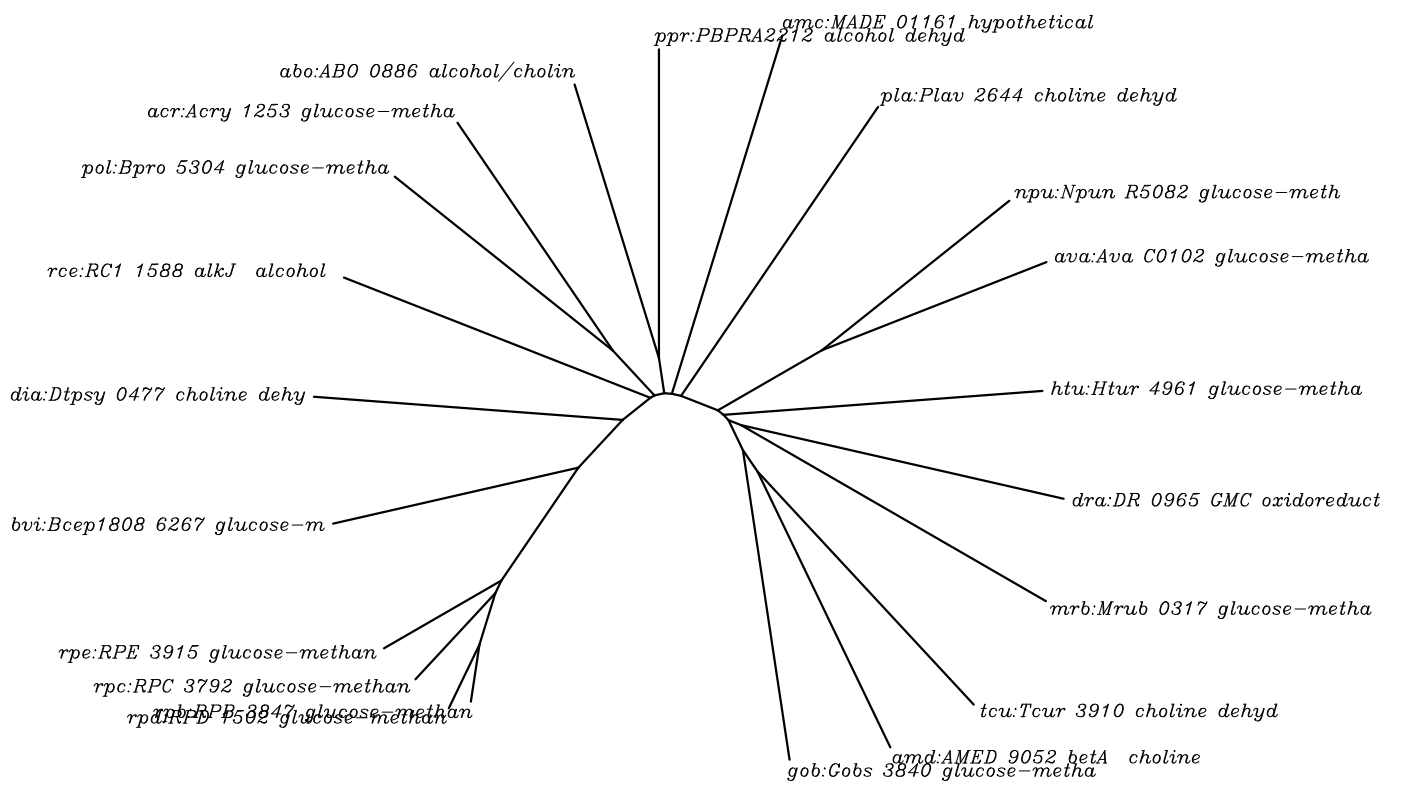

Supplement: Additional file 1 — Collection of phylogenetic trees for Thermoprotei and Halobacteria LGT genes with strong matches. Trees for all LGT genes with BLAST scores greater than 500 in both the Thermoprotei and Halobacteria. The KEGG database three letter genome code is given before the colon and can be found here http://www.genome.jp/kegg/catalog/org_list.html. The corresponding gene locus tags are provided after the colon. [file 1471-2148-11-199-S1.GZ › Trees/Htree53.pdf]

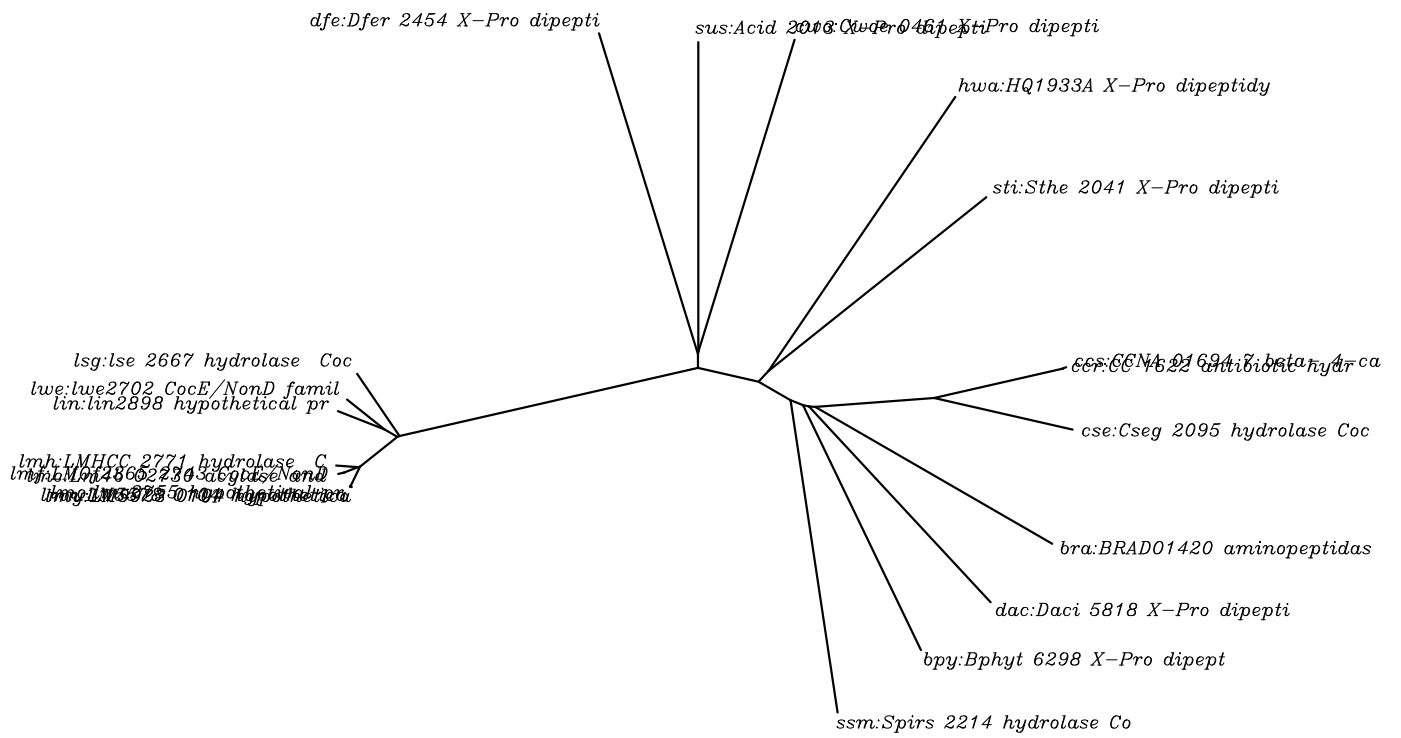

Supplement: Additional file 1 — Collection of phylogenetic trees for Thermoprotei and Halobacteria LGT genes with strong matches. Trees for all LGT genes with BLAST scores greater than 500 in both the Thermoprotei and Halobacteria. The KEGG database three letter genome code is given before the colon and can be found here http://www.genome.jp/kegg/catalog/org_list.html. The corresponding gene locus tags are provided after the colon. [file 1471-2148-11-199-S1.GZ › Trees/Htree54.pdf]

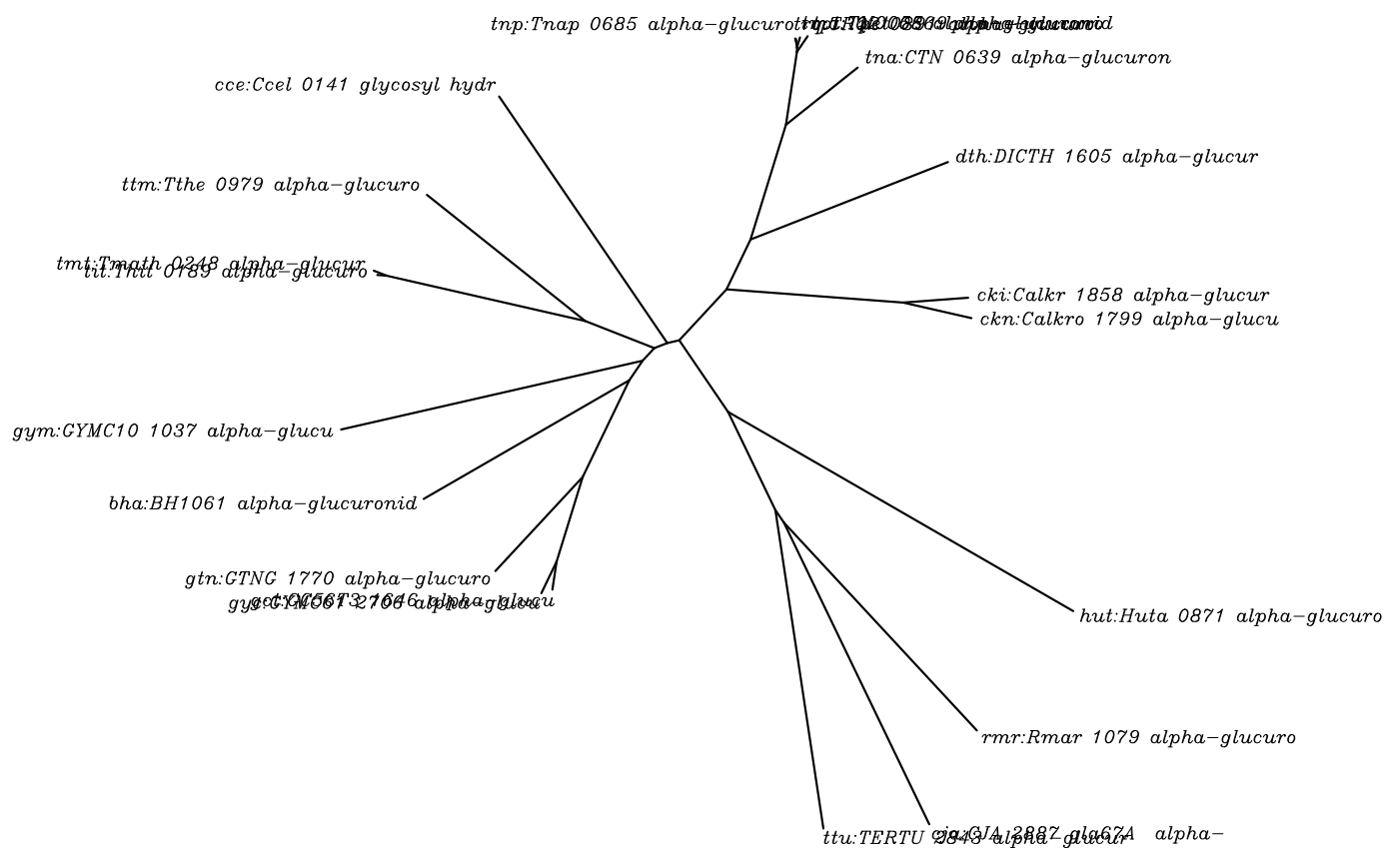

Supplement: Additional file 1 — Collection of phylogenetic trees for Thermoprotei and Halobacteria LGT genes with strong matches. Trees for all LGT genes with BLAST scores greater than 500 in both the Thermoprotei and Halobacteria. The KEGG database three letter genome code is given before the colon and can be found here http://www.genome.jp/kegg/catalog/org_list.html. The corresponding gene locus tags are provided after the colon. [file 1471-2148-11-199-S1.GZ › Trees/Htree55.pdf]

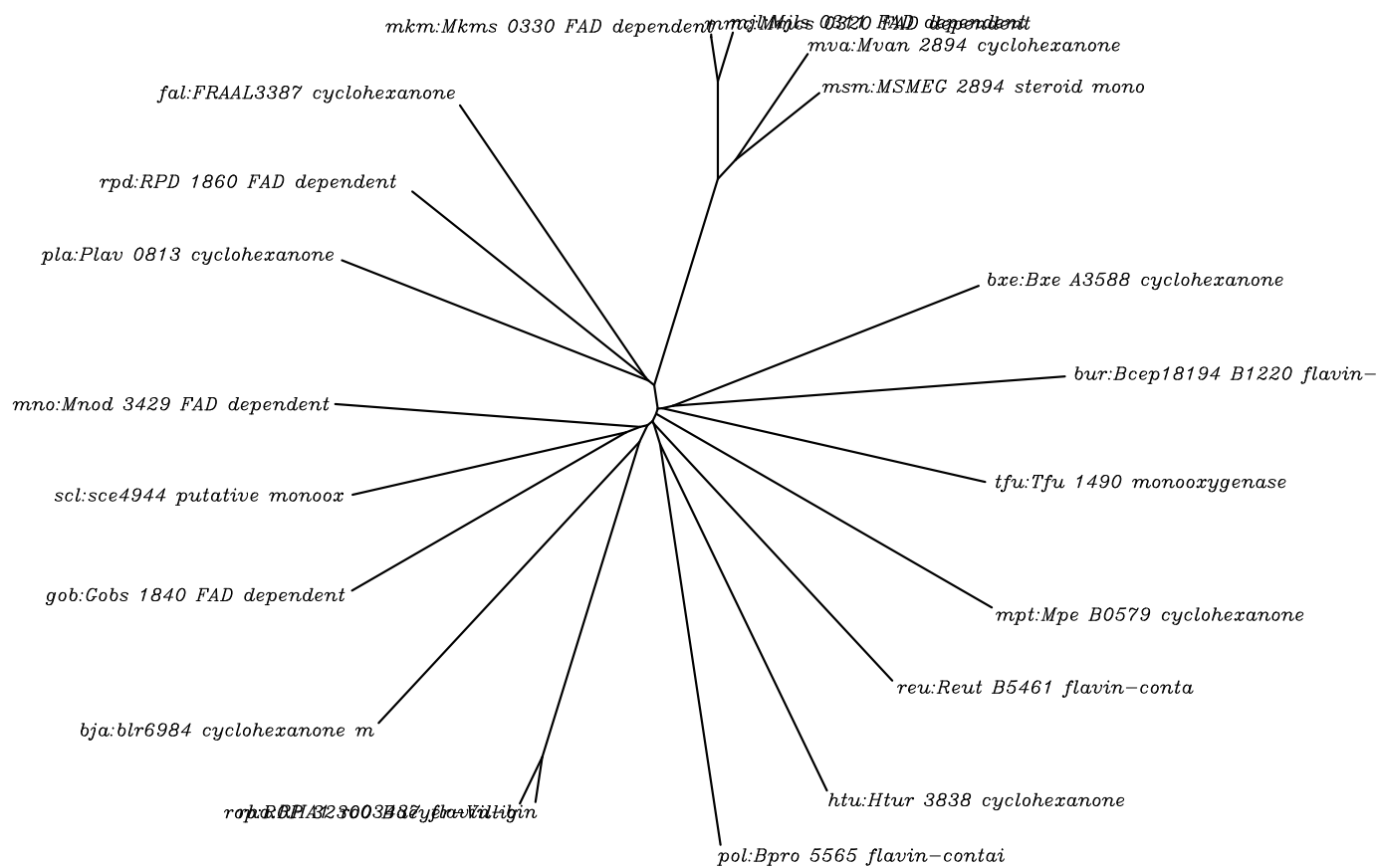

Supplement: Additional file 1 — Collection of phylogenetic trees for Thermoprotei and Halobacteria LGT genes with strong matches. Trees for all LGT genes with BLAST scores greater than 500 in both the Thermoprotei and Halobacteria. The KEGG database three letter genome code is given before the colon and can be found here http://www.genome.jp/kegg/catalog/org_list.html. The corresponding gene locus tags are provided after the colon. [file 1471-2148-11-199-S1.GZ › Trees/Htree56.pdf]

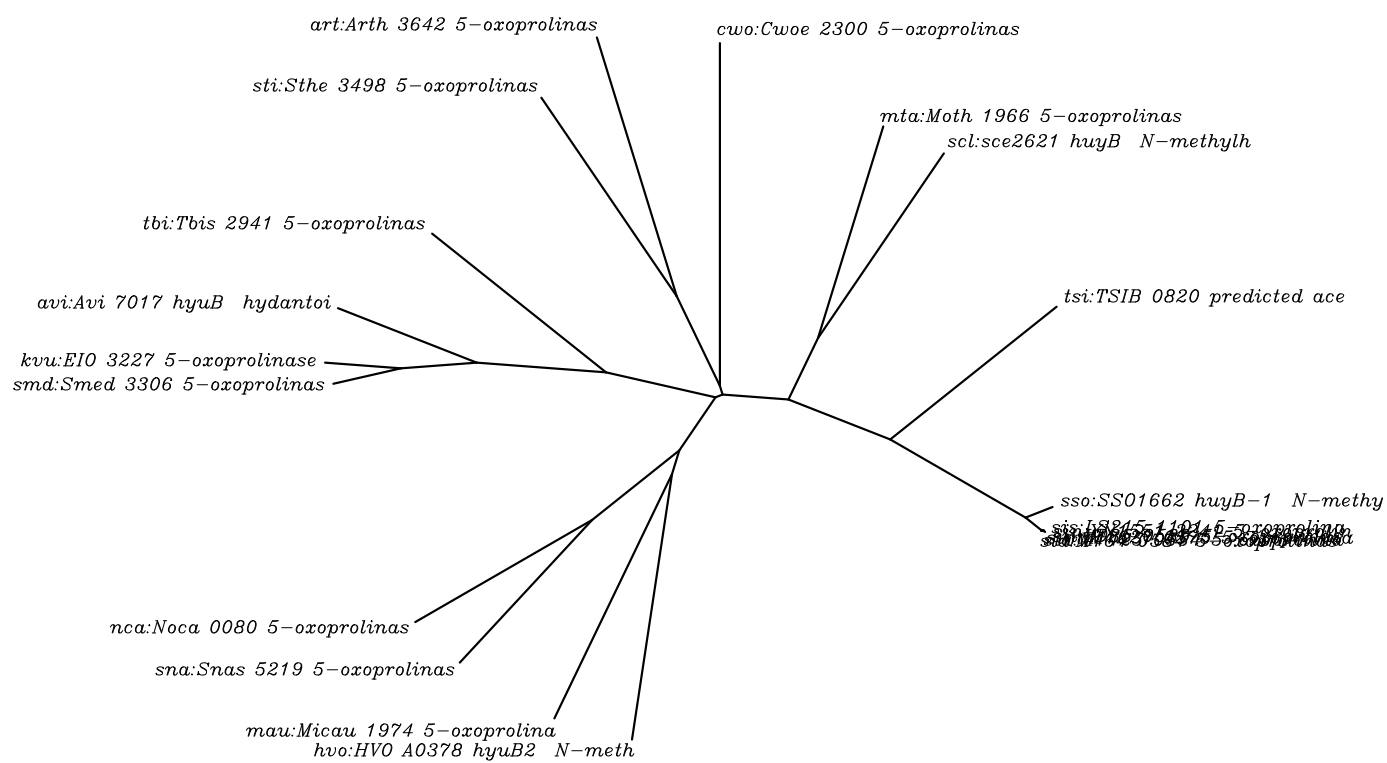

Supplement: Additional file 1 — Collection of phylogenetic trees for Thermoprotei and Halobacteria LGT genes with strong matches. Trees for all LGT genes with BLAST scores greater than 500 in both the Thermoprotei and Halobacteria. The KEGG database three letter genome code is given before the colon and can be found here http://www.genome.jp/kegg/catalog/org_list.html. The corresponding gene locus tags are provided after the colon. [file 1471-2148-11-199-S1.GZ › Trees/Htree57.pdf]

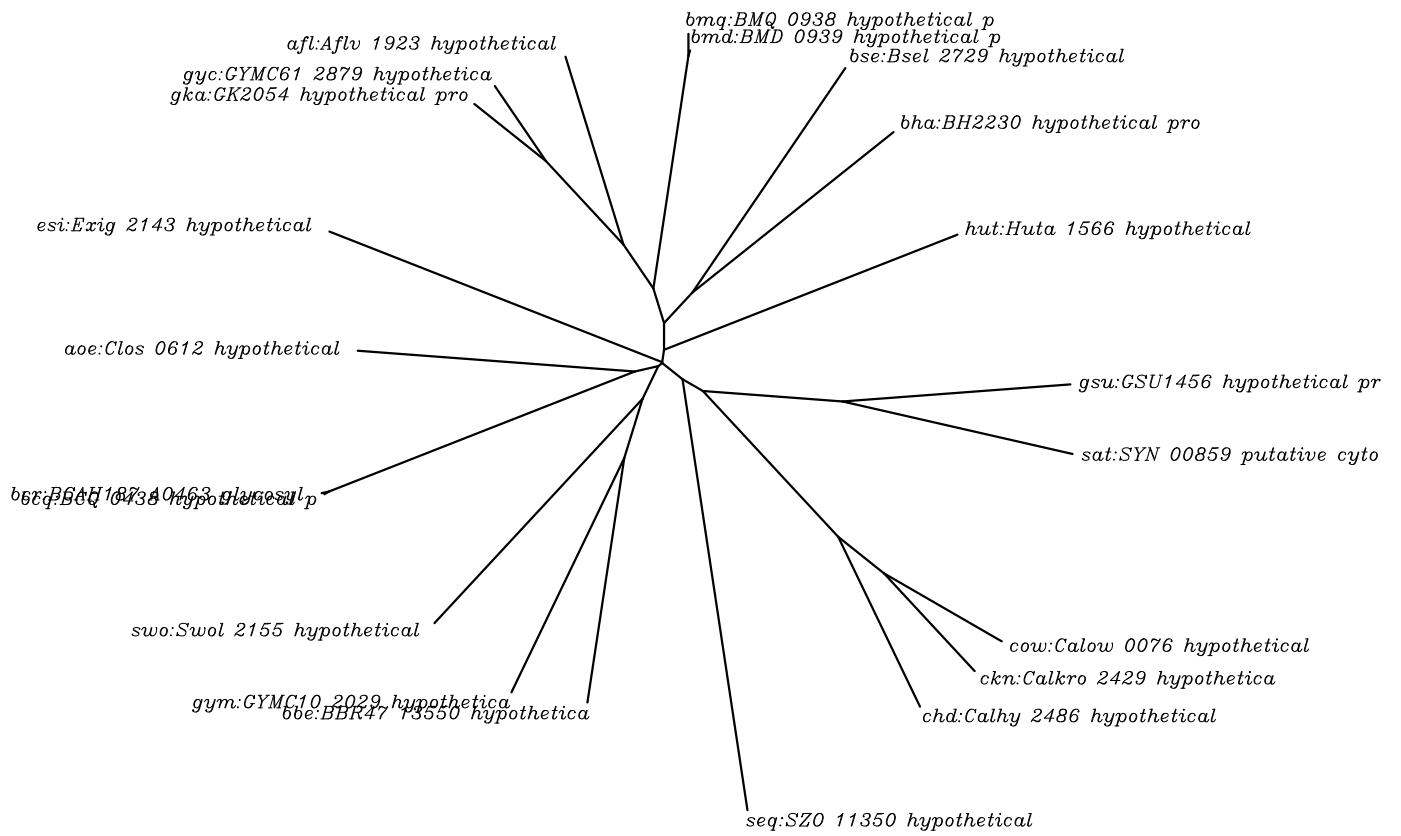

Supplement: Additional file 1 — Collection of phylogenetic trees for Thermoprotei and Halobacteria LGT genes with strong matches. Trees for all LGT genes with BLAST scores greater than 500 in both the Thermoprotei and Halobacteria. The KEGG database three letter genome code is given before the colon and can be found here http://www.genome.jp/kegg/catalog/org_list.html. The corresponding gene locus tags are provided after the colon. [file 1471-2148-11-199-S1.GZ › Trees/Htree58.pdf]

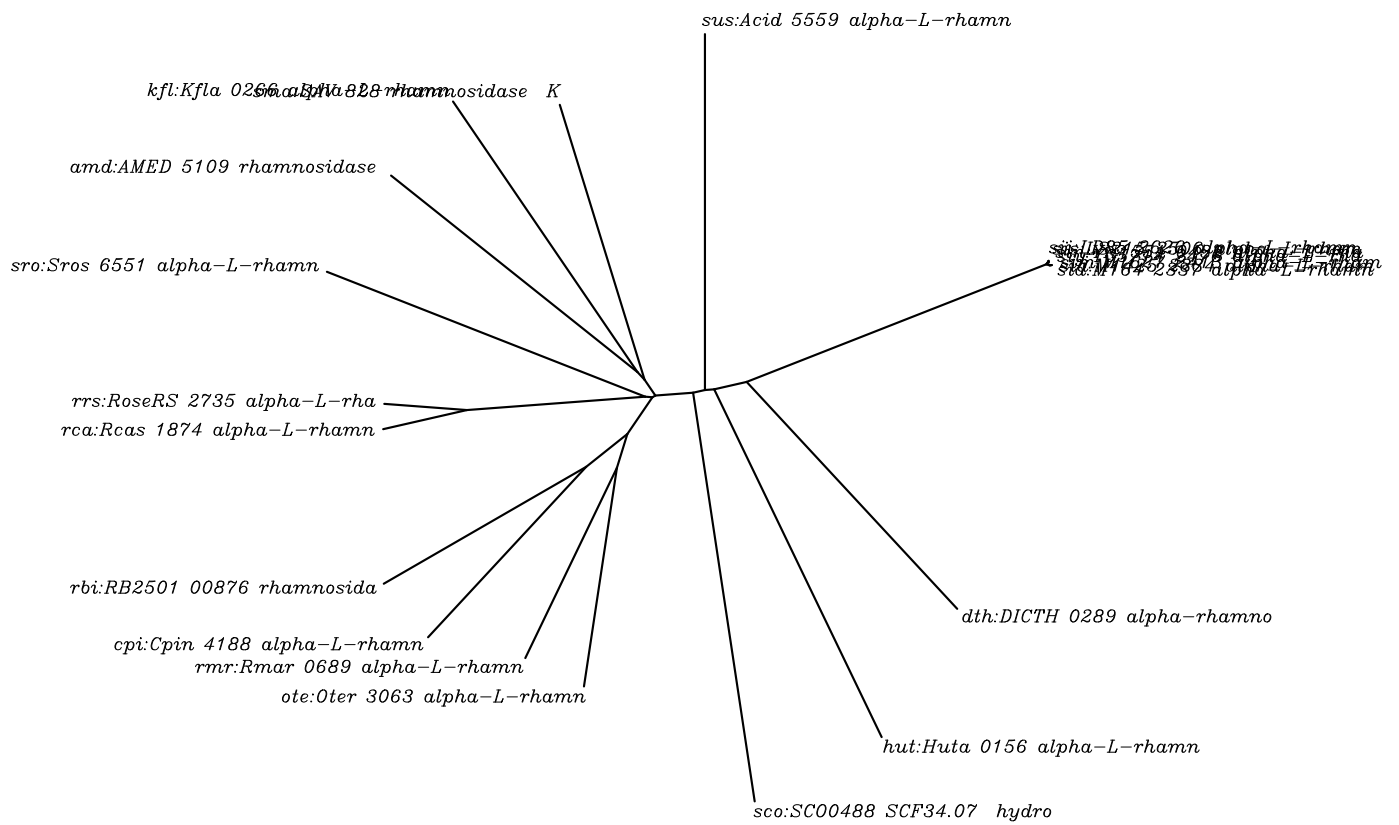

Supplement: Additional file 1 — Collection of phylogenetic trees for Thermoprotei and Halobacteria LGT genes with strong matches. Trees for all LGT genes with BLAST scores greater than 500 in both the Thermoprotei and Halobacteria. The KEGG database three letter genome code is given before the colon and can be found here http://www.genome.jp/kegg/catalog/org_list.html. The corresponding gene locus tags are provided after the colon. [file 1471-2148-11-199-S1.GZ › Trees/Htree59.pdf]

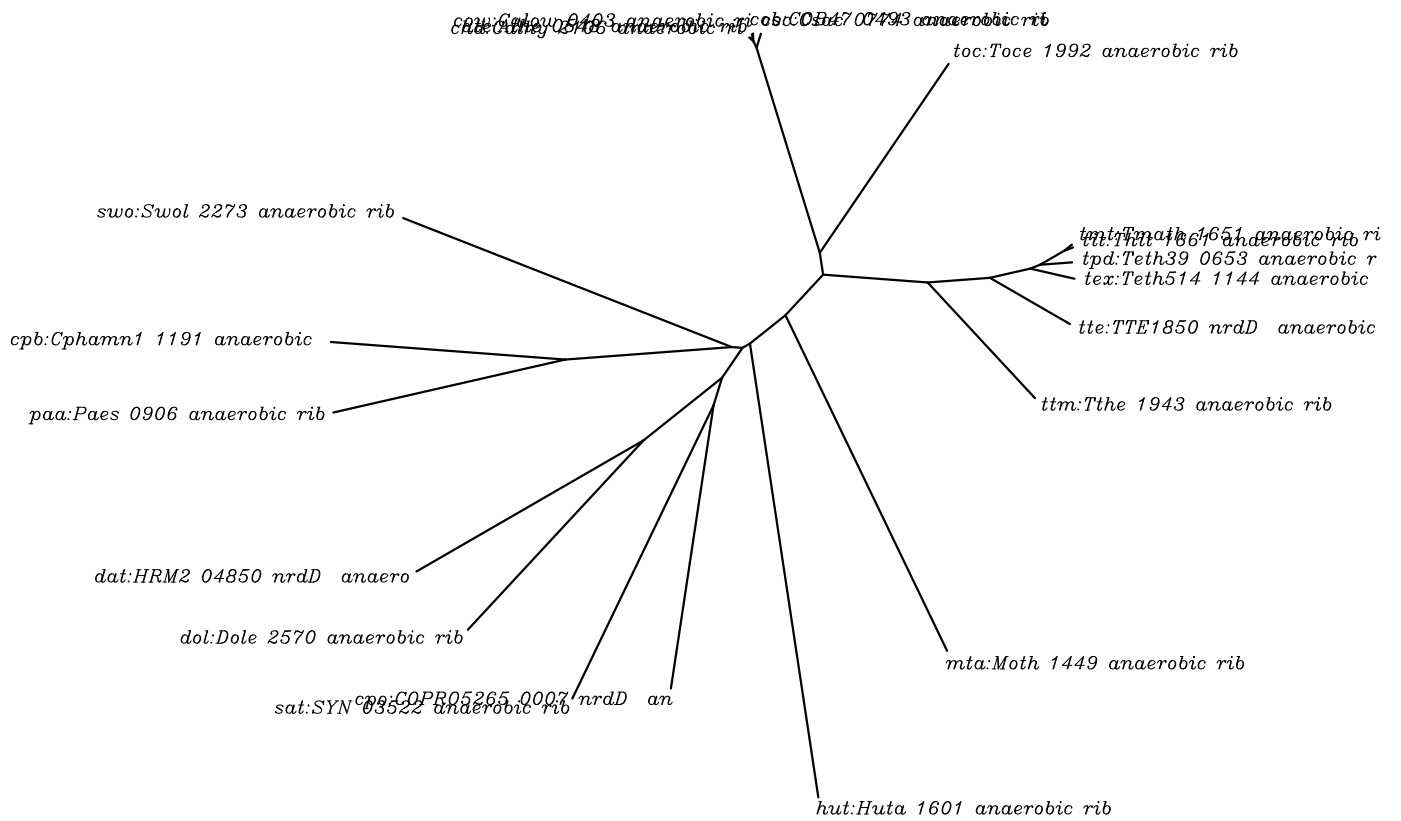

Supplement: Additional file 1 — Collection of phylogenetic trees for Thermoprotei and Halobacteria LGT genes with strong matches. Trees for all LGT genes with BLAST scores greater than 500 in both the Thermoprotei and Halobacteria. The KEGG database three letter genome code is given before the colon and can be found here http://www.genome.jp/kegg/catalog/org_list.html. The corresponding gene locus tags are provided after the colon. [file 1471-2148-11-199-S1.GZ › Trees/Htree6.pdf]

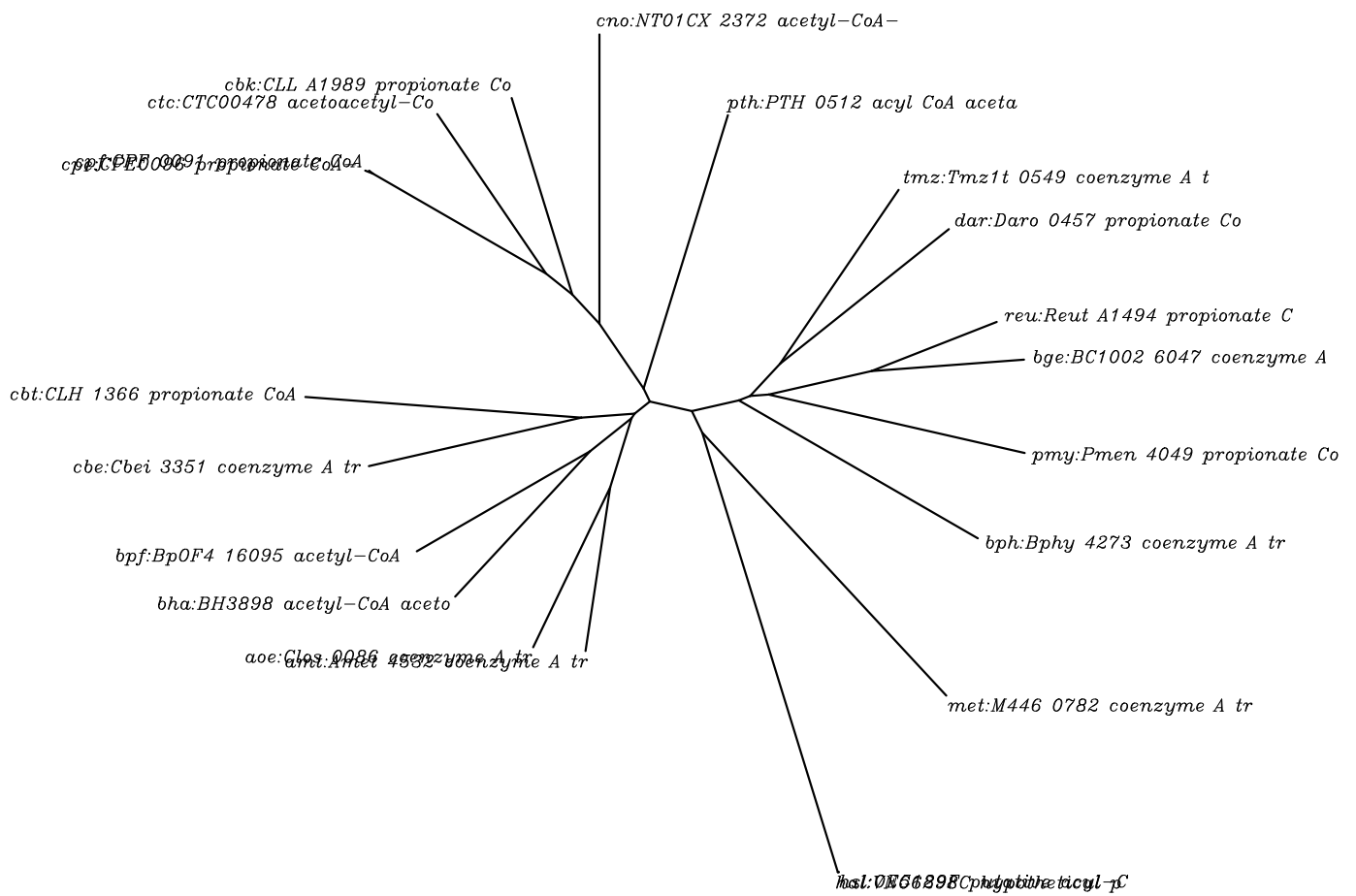

Supplement: Additional file 1 — Collection of phylogenetic trees for Thermoprotei and Halobacteria LGT genes with strong matches. Trees for all LGT genes with BLAST scores greater than 500 in both the Thermoprotei and Halobacteria. The KEGG database three letter genome code is given before the colon and can be found here http://www.genome.jp/kegg/catalog/org_list.html. The corresponding gene locus tags are provided after the colon. [file 1471-2148-11-199-S1.GZ › Trees/Htree60.pdf]

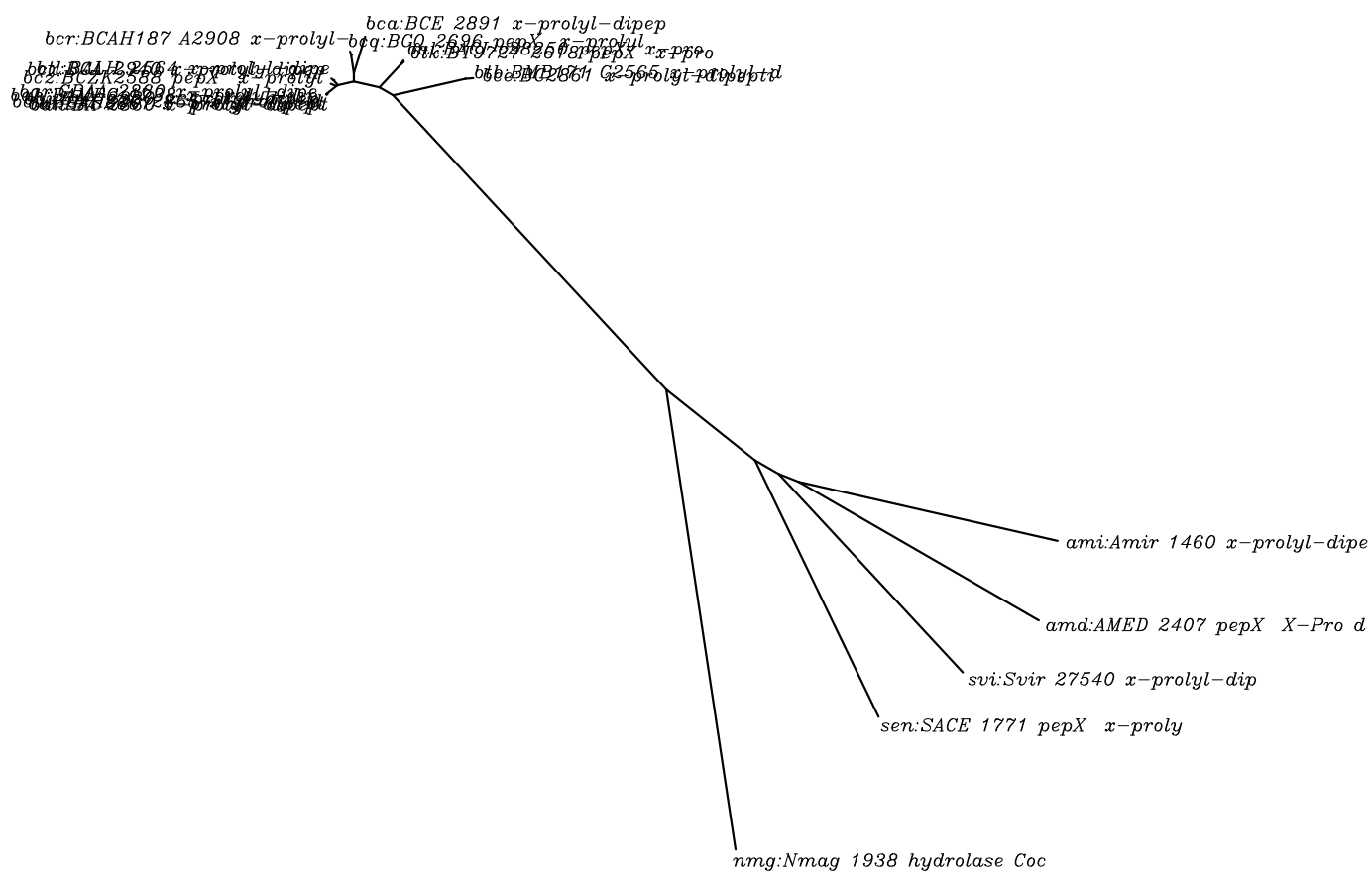

Supplement: Additional file 1 — Collection of phylogenetic trees for Thermoprotei and Halobacteria LGT genes with strong matches. Trees for all LGT genes with BLAST scores greater than 500 in both the Thermoprotei and Halobacteria. The KEGG database three letter genome code is given before the colon and can be found here http://www.genome.jp/kegg/catalog/org_list.html. The corresponding gene locus tags are provided after the colon. [file 1471-2148-11-199-S1.GZ › Trees/Htree61.pdf]

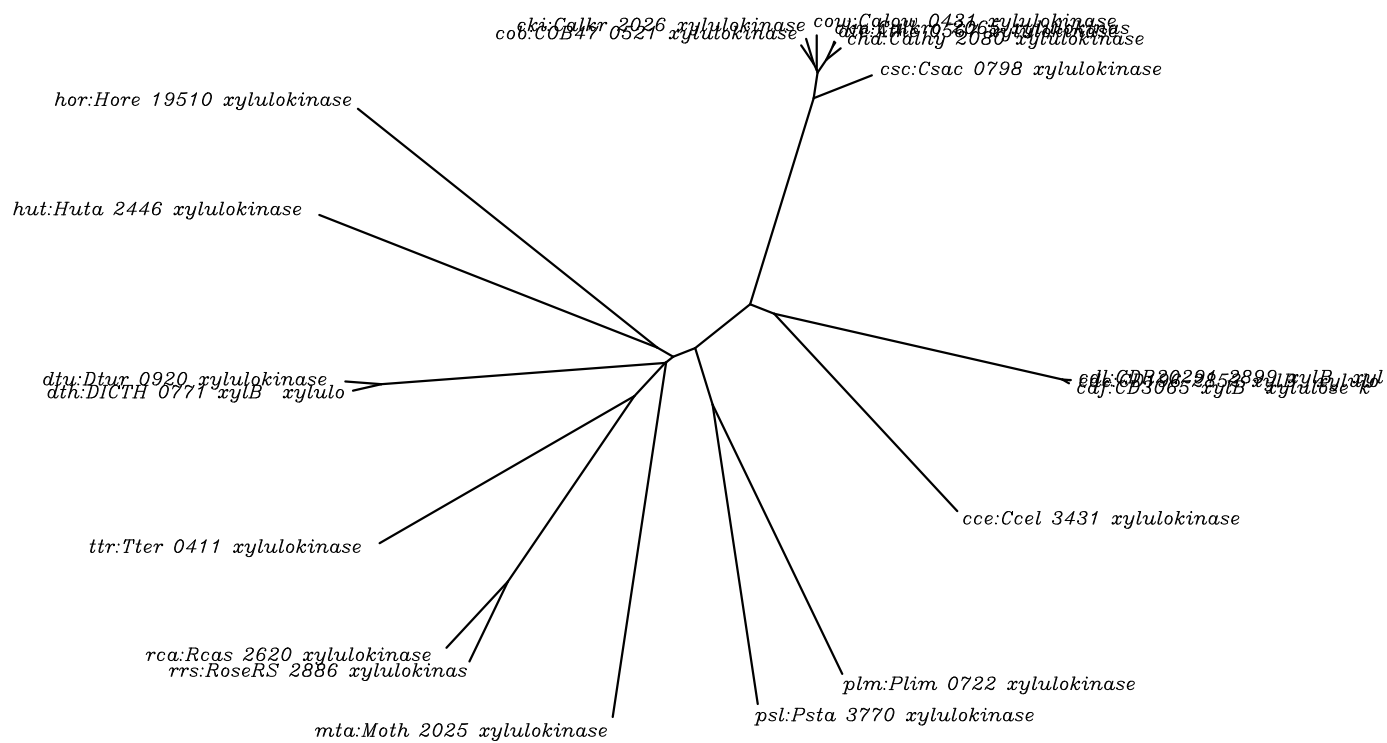

Supplement: Additional file 1 — Collection of phylogenetic trees for Thermoprotei and Halobacteria LGT genes with strong matches. Trees for all LGT genes with BLAST scores greater than 500 in both the Thermoprotei and Halobacteria. The KEGG database three letter genome code is given before the colon and can be found here http://www.genome.jp/kegg/catalog/org_list.html. The corresponding gene locus tags are provided after the colon. [file 1471-2148-11-199-S1.GZ › Trees/Htree62.pdf]

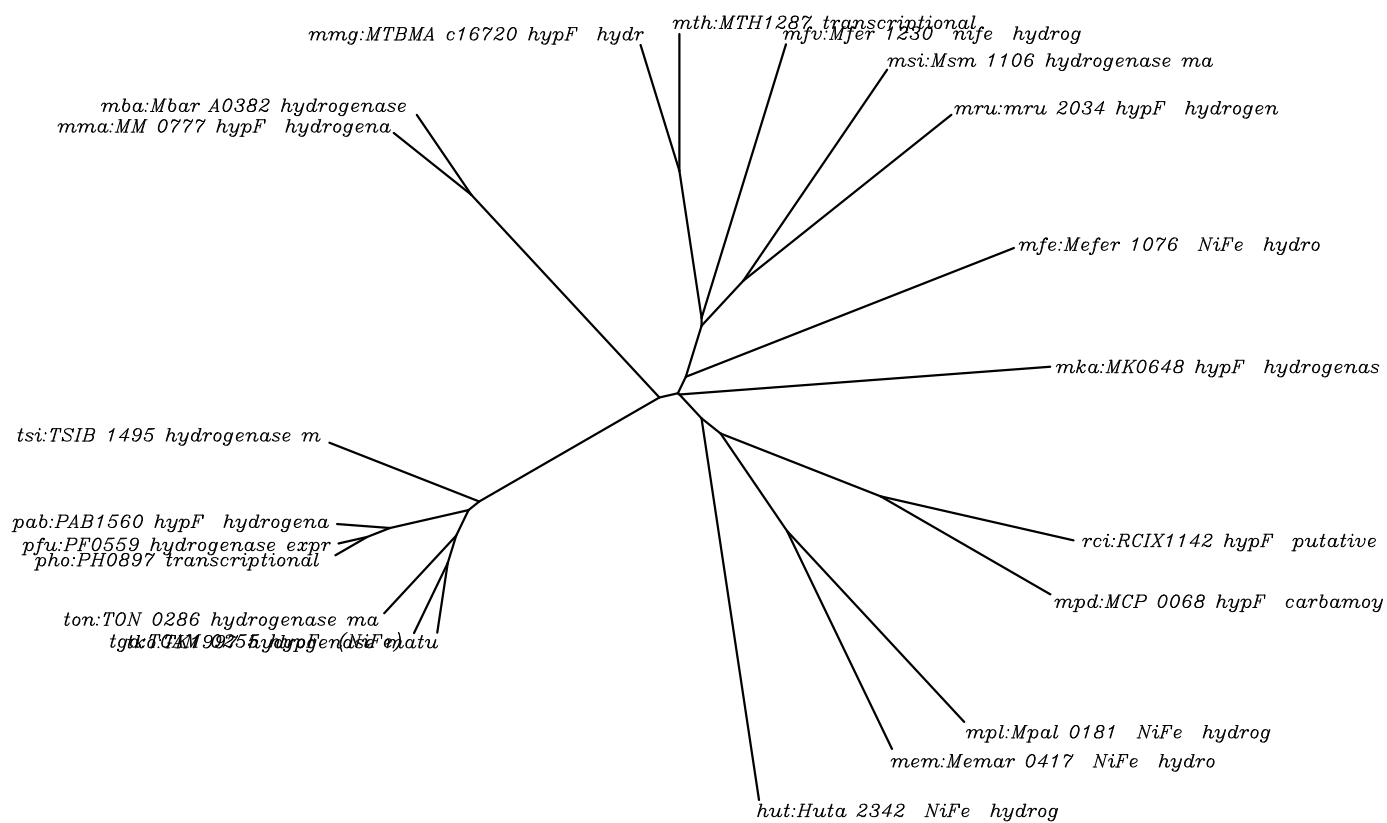

Supplement: Additional file 1 — Collection of phylogenetic trees for Thermoprotei and Halobacteria LGT genes with strong matches. Trees for all LGT genes with BLAST scores greater than 500 in both the Thermoprotei and Halobacteria. The KEGG database three letter genome code is given before the colon and can be found here http://www.genome.jp/kegg/catalog/org_list.html. The corresponding gene locus tags are provided after the colon. [file 1471-2148-11-199-S1.GZ › Trees/Htree63.pdf]

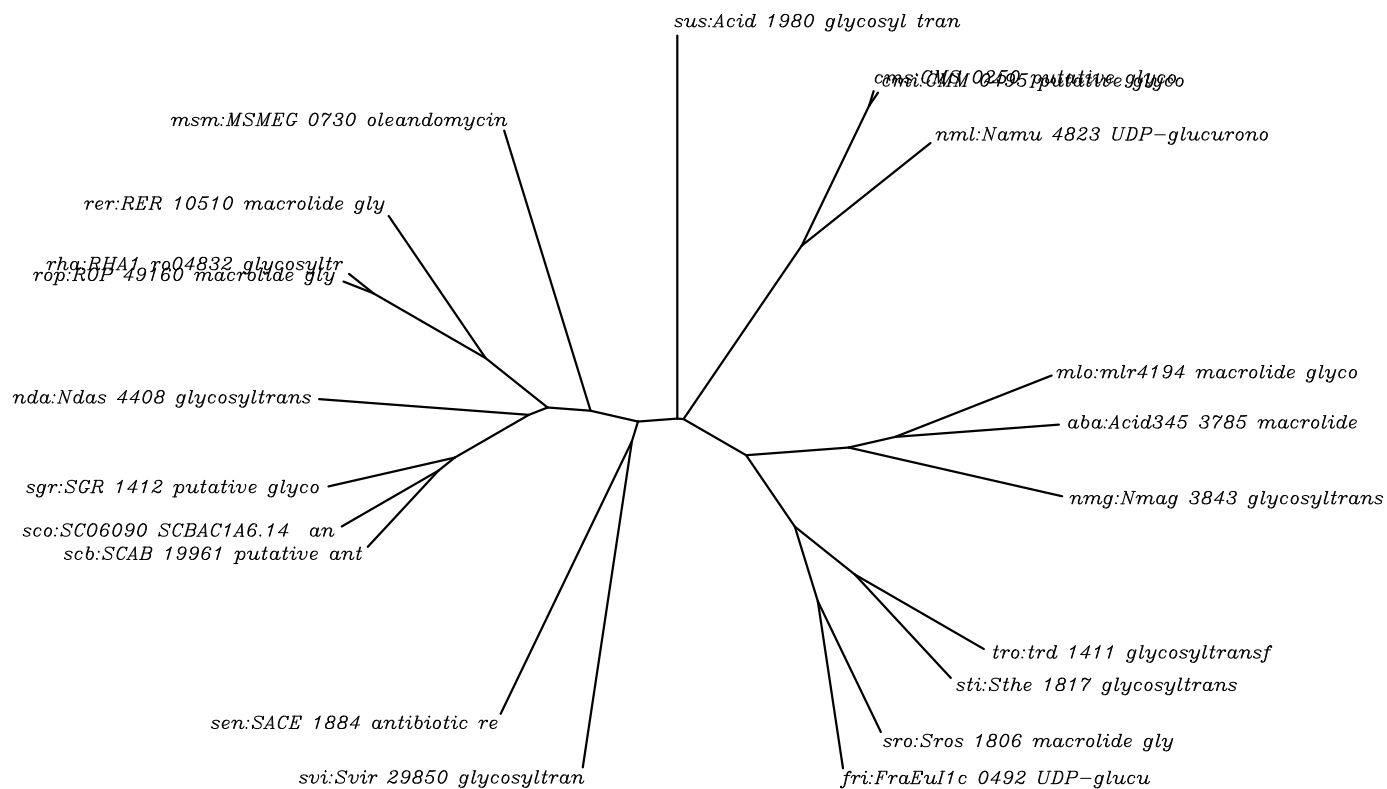

Supplement: Additional file 1 — Collection of phylogenetic trees for Thermoprotei and Halobacteria LGT genes with strong matches. Trees for all LGT genes with BLAST scores greater than 500 in both the Thermoprotei and Halobacteria. The KEGG database three letter genome code is given before the colon and can be found here http://www.genome.jp/kegg/catalog/org_list.html. The corresponding gene locus tags are provided after the colon. [file 1471-2148-11-199-S1.GZ › Trees/Htree64.pdf]

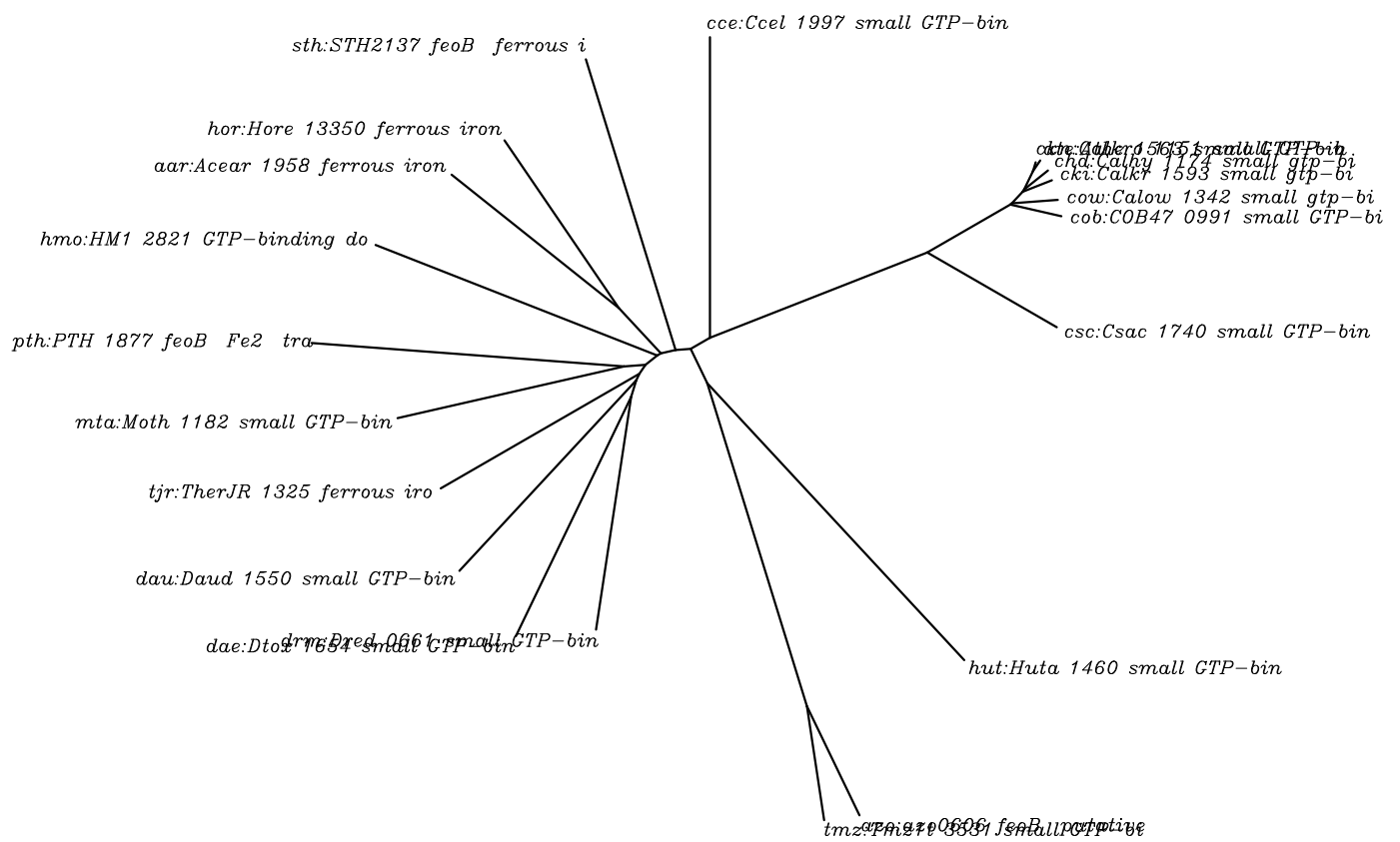

Supplement: Additional file 1 — Collection of phylogenetic trees for Thermoprotei and Halobacteria LGT genes with strong matches. Trees for all LGT genes with BLAST scores greater than 500 in both the Thermoprotei and Halobacteria. The KEGG database three letter genome code is given before the colon and can be found here http://www.genome.jp/kegg/catalog/org_list.html. The corresponding gene locus tags are provided after the colon. [file 1471-2148-11-199-S1.GZ › Trees/Htree65.pdf]

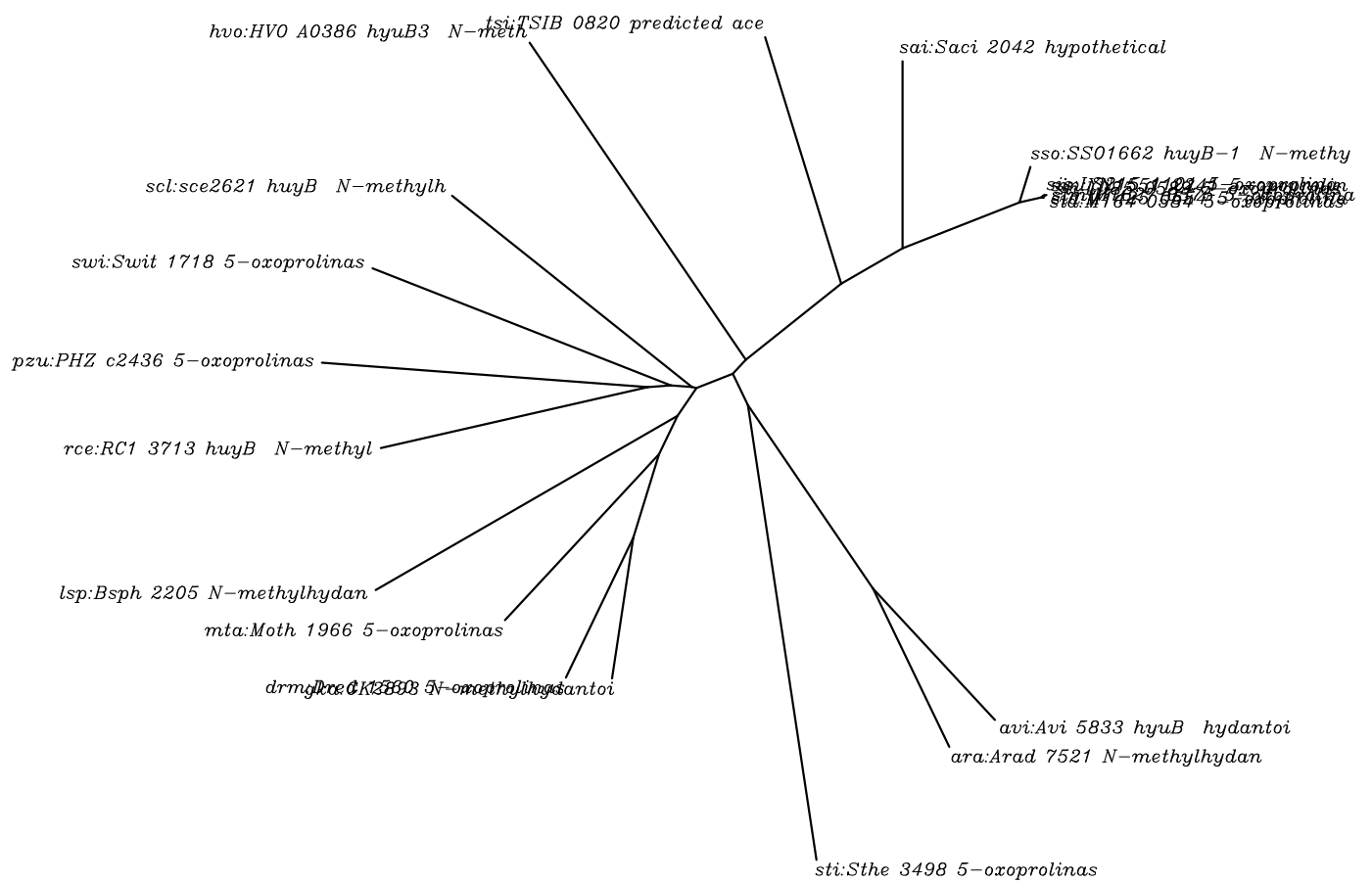

Supplement: Additional file 1 — Collection of phylogenetic trees for Thermoprotei and Halobacteria LGT genes with strong matches. Trees for all LGT genes with BLAST scores greater than 500 in both the Thermoprotei and Halobacteria. The KEGG database three letter genome code is given before the colon and can be found here http://www.genome.jp/kegg/catalog/org_list.html. The corresponding gene locus tags are provided after the colon. [file 1471-2148-11-199-S1.GZ › Trees/Htree66.pdf]

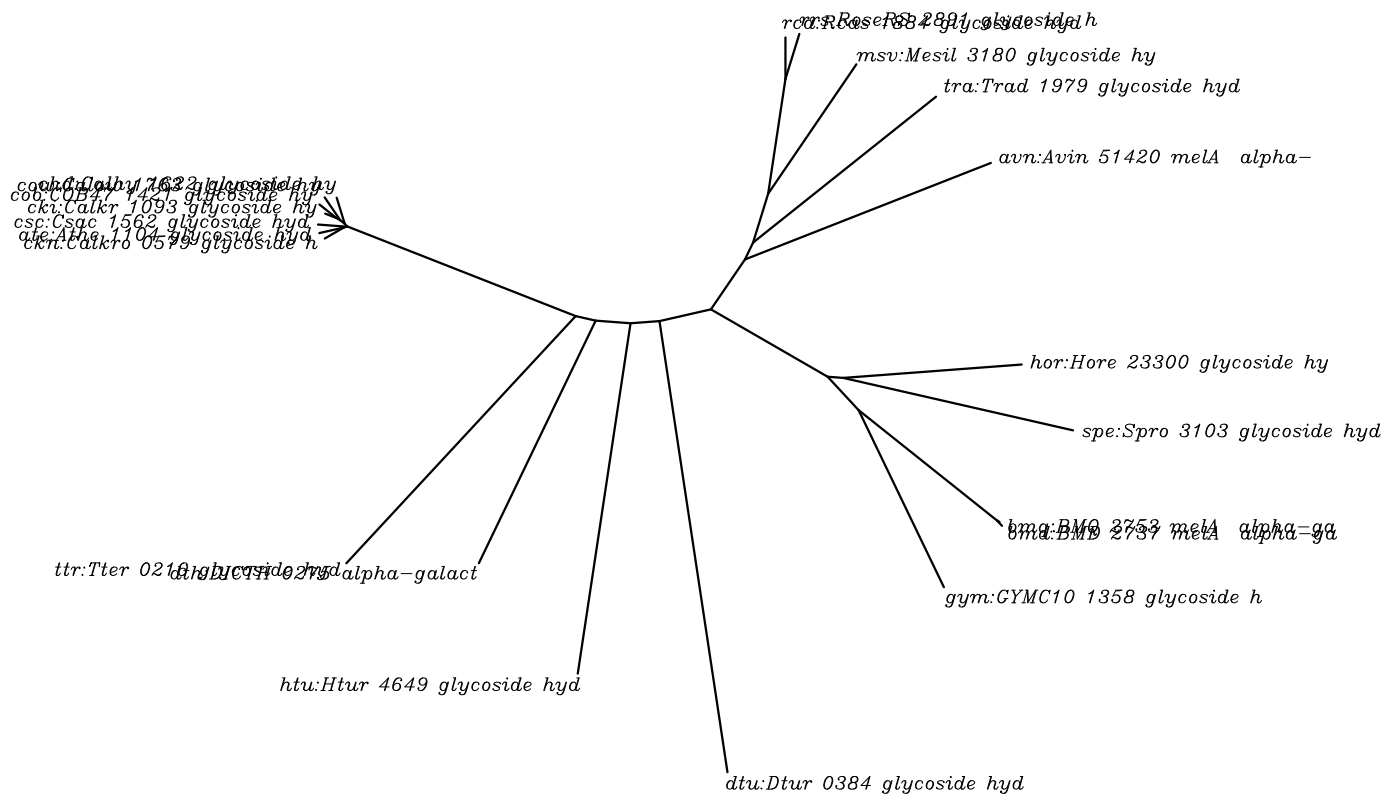

Supplement: Additional file 1 — Collection of phylogenetic trees for Thermoprotei and Halobacteria LGT genes with strong matches. Trees for all LGT genes with BLAST scores greater than 500 in both the Thermoprotei and Halobacteria. The KEGG database three letter genome code is given before the colon and can be found here http://www.genome.jp/kegg/catalog/org_list.html. The corresponding gene locus tags are provided after the colon. [file 1471-2148-11-199-S1.GZ › Trees/Htree67.pdf]

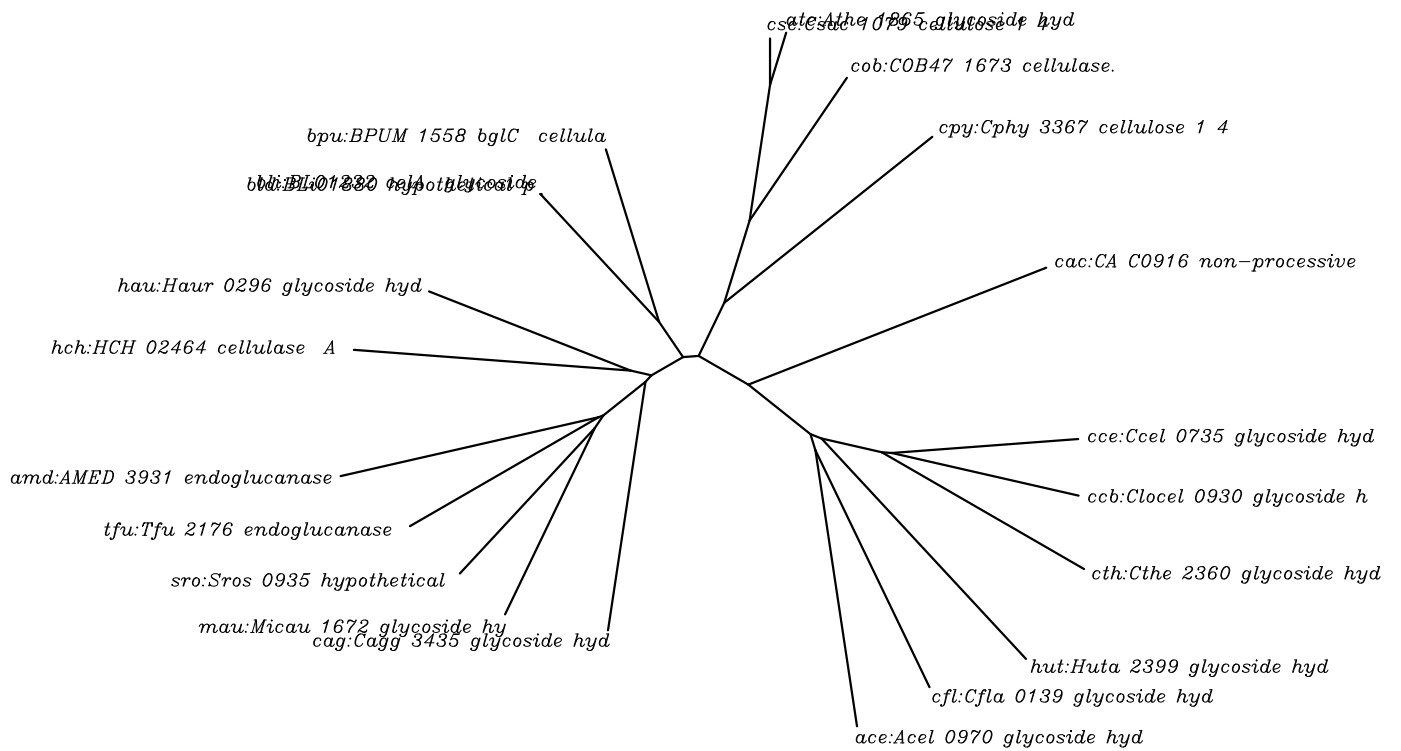

Supplement: Additional file 1 — Collection of phylogenetic trees for Thermoprotei and Halobacteria LGT genes with strong matches. Trees for all LGT genes with BLAST scores greater than 500 in both the Thermoprotei and Halobacteria. The KEGG database three letter genome code is given before the colon and can be found here http://www.genome.jp/kegg/catalog/org_list.html. The corresponding gene locus tags are provided after the colon. [file 1471-2148-11-199-S1.GZ › Trees/Htree7.pdf]

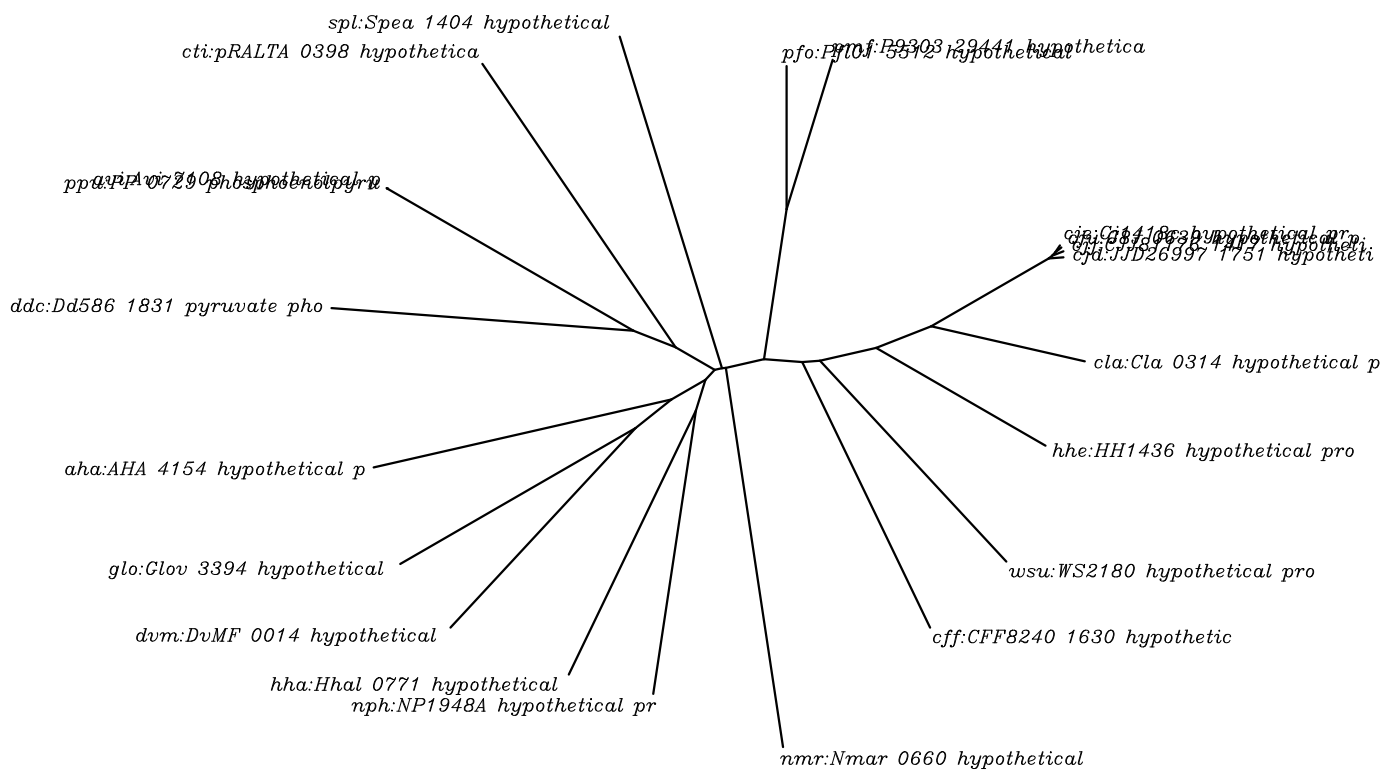

Supplement: Additional file 1 — Collection of phylogenetic trees for Thermoprotei and Halobacteria LGT genes with strong matches. Trees for all LGT genes with BLAST scores greater than 500 in both the Thermoprotei and Halobacteria. The KEGG database three letter genome code is given before the colon and can be found here http://www.genome.jp/kegg/catalog/org_list.html. The corresponding gene locus tags are provided after the colon. [file 1471-2148-11-199-S1.GZ › Trees/Htree8.pdf]

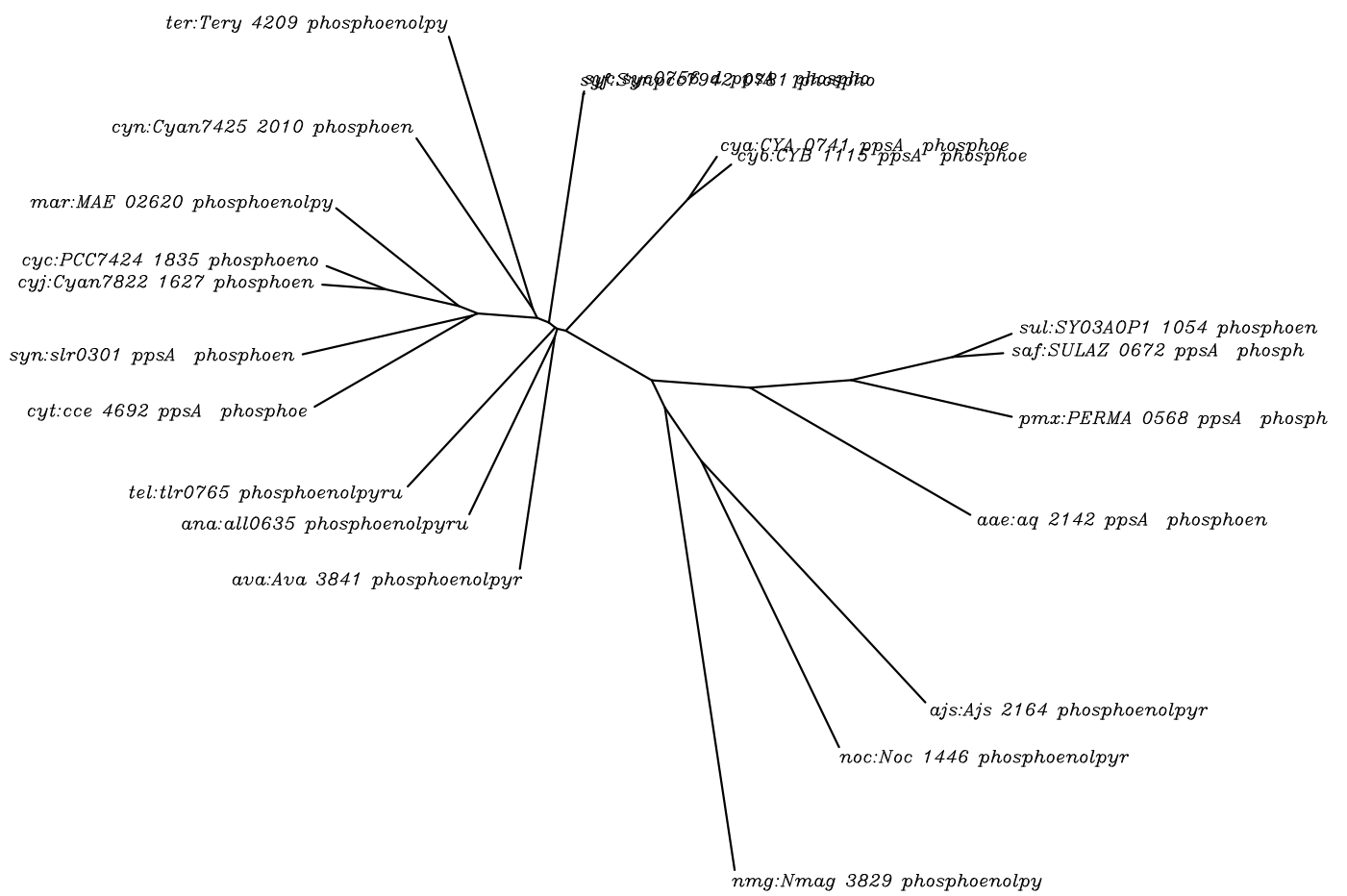

Supplement: Additional file 1 — Collection of phylogenetic trees for Thermoprotei and Halobacteria LGT genes with strong matches. Trees for all LGT genes with BLAST scores greater than 500 in both the Thermoprotei and Halobacteria. The KEGG database three letter genome code is given before the colon and can be found here http://www.genome.jp/kegg/catalog/org_list.html. The corresponding gene locus tags are provided after the colon. [file 1471-2148-11-199-S1.GZ › Trees/Htree9.pdf]

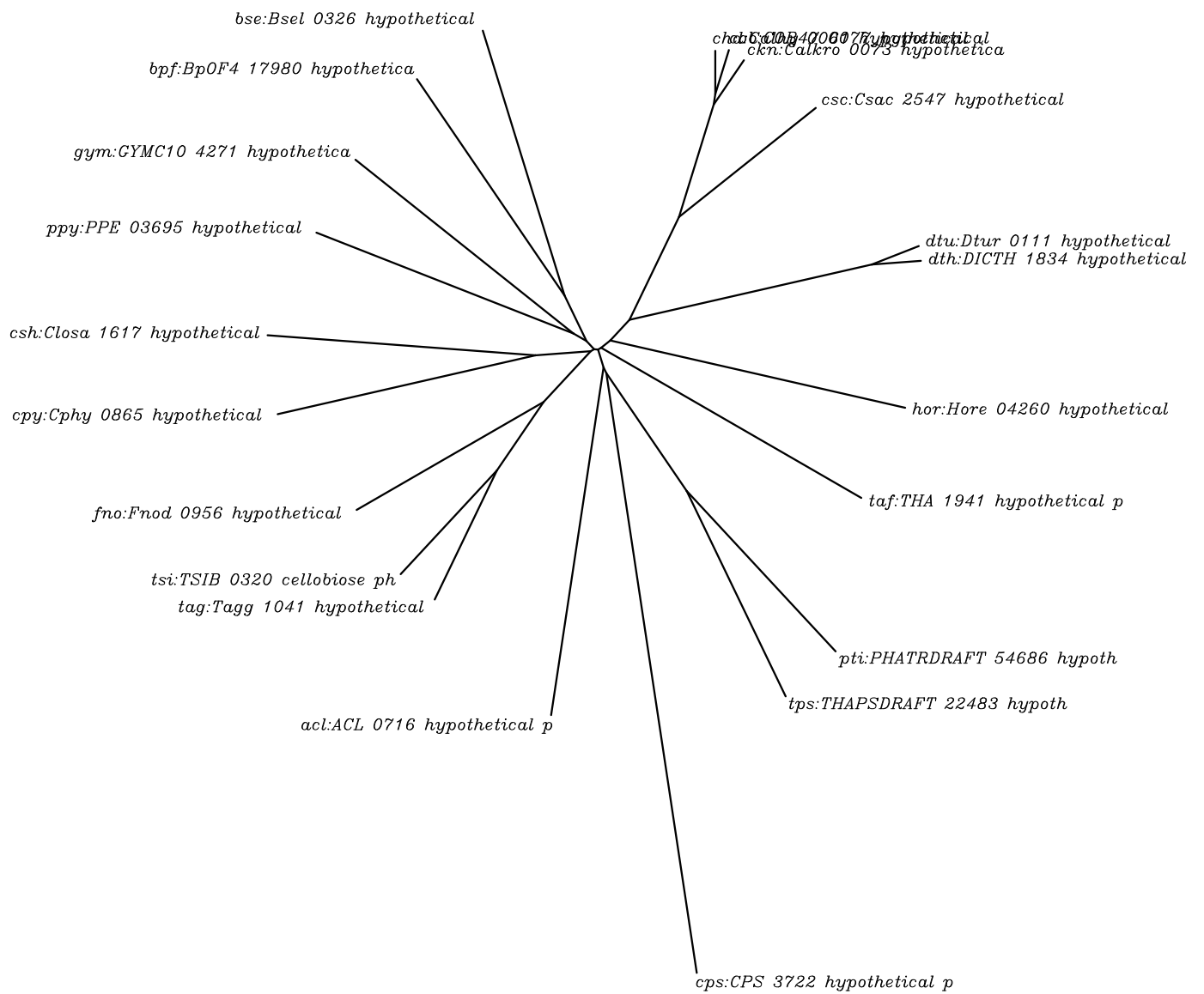

Supplement: Additional file 1 — Collection of phylogenetic trees for Thermoprotei and Halobacteria LGT genes with strong matches. Trees for all LGT genes with BLAST scores greater than 500 in both the Thermoprotei and Halobacteria. The KEGG database three letter genome code is given before the colon and can be found here http://www.genome.jp/kegg/catalog/org_list.html. The corresponding gene locus tags are provided after the colon. [file 1471-2148-11-199-S1.GZ › Trees/Ttree1.pdf]
